# Myosin IIa Promotes Antibody Responses by Regulating B Cell Activation, Acquisition of Antigen, and Proliferation

**DOI:** 10.1016/j.celrep.2018.04.087

**Published:** 2018-05-22

**Authors:** Robbert Hoogeboom, Elizabeth M. Natkanski, Carla R. Nowosad, Dessislava Malinova, Rajesh P. Menon, Antonio Casal, Pavel Tolar

**Affiliations:** 1 Immune Receptor Activation Laboratory, The Francis Crick Institute, London NW1 1AT, UK; 2 Department of Haemato-Oncology, Faculty of Life Sciences and Medicine, King’s College London, London SE5 9NU, UK; 3 Division of Immunology & Inflammation, Department of Medicine, Imperial College London, London SW7 2A2, UK

**Keywords:** B cell response, B cell development, B cell signaling, antigen internalization, antigen presentation, cytoskeleton, non-muscle myosin

## Abstract

B cell responses are regulated by antigen acquisition, processing, and presentation to helper T cells. These functions are thought to depend on contractile activity of non-muscle myosin IIa. Here, we show that B cell-specific deletion of the myosin IIa heavy chain reduced the numbers of bone marrow B cell precursors and splenic marginal zone, peritoneal B1b, and germinal center B cells. In addition, myosin IIa-deficient follicular B cells acquired an activated phenotype and were less efficient in chemokinesis and extraction of membrane-presented antigens. Moreover, myosin IIa was indispensable for cytokinesis. Consequently, mice with myosin IIa-deficient B cells harbored reduced serum immunoglobulin levels and did not mount robust antibody responses when immunized. Altogether, these data indicate that myosin IIa is a negative regulator of B cell activation but a positive regulator of antigen acquisition from antigen-presenting cells and that myosin IIa is essential for B cell development, proliferation, and antibody responses.

## Highlights

• Myosin IIa is important for B cell antigen acquisition from antigen-presenting cells
• Myosin IIa is a negative regulator of B cell activation
• Myosin IIa is essential for B cell cytokinesis
• Myosin IIa is required for efficient B cell responses

## Graphical Abstract

**Figure:**
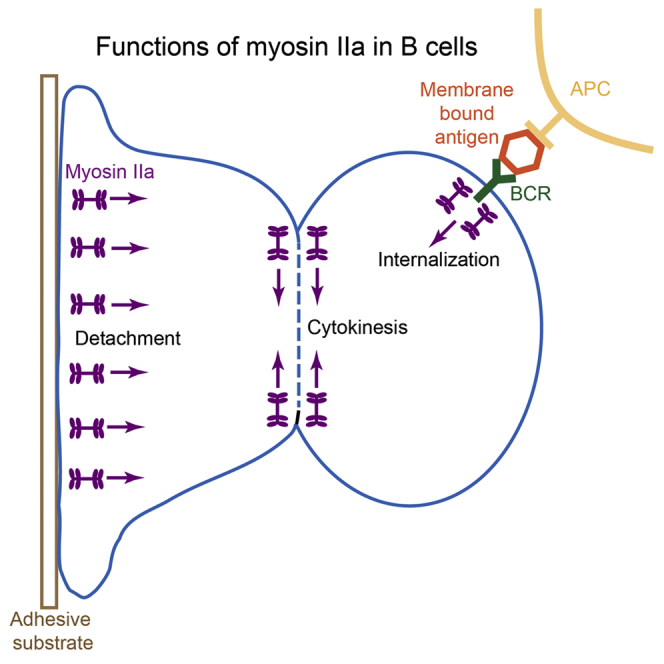

## Teaser

B cell antigen acquisition, processing, and presentation may depend on contractile activity of the actomyosin cytoskeleton. Here, Hoogeboom et al. show that non-muscle myosin IIa positively regulates B cell antigen acquisition from antigen-presenting cells *in vivo*. In addition, myosin IIa negatively regulates B cell activation and is required for B cell cytokinesis.

## Introduction

B cell activation is initiated when B cells bind antigen via their cell-surface B cell antigen receptors (BCRs). This induces signaling and internalization of BCR-antigen complexes. Subsequently, antigen is trafficked along the endosomal pathway, processed into peptides, and loaded on major histocompatibility complex class II (MHC class II) molecules for presentation to T cells. Cognate interaction with T cells results in full activation and proliferation of the B cell and differentiation into high-affinity antibody-secreting cells. Some B cells, e.g., marginal zone (MZ) B cells, mostly encounter small soluble antigens. However, many B cells engage antigen bound to antigen-presenting cells (APCs), such as subcapsular macrophages and follicular dendritic cells (FDCs), which display unprocessed antigen bound to complement or Fc receptors on their cell surfaces (Carrasco and Batista, 2007, Gonzalez et al., 2010, Junt et al., 2007, Phan et al., 2007, Qi et al., 2006). In contrast to acquisition of free-floating soluble antigen, capture of membrane-bound antigens from APCs requires B cells to apply force to overcome the membrane tether. It has been hypothesized that non-muscle myosin IIa generates these forces.

Myosin IIa is a motor protein from the class II family of myosin proteins, which have been implicated in generation of cortical tension (Murrell et al., 2015), separation of the mitotic spindle (Rosenblatt et al., 2004), formation of the cleavage furrow during cytokinesis (Straight et al., 2003), and cellular locomotion (Kolega, 1998). Myosin IIa is the only class II myosin expressed in lymphocytes. In T cells, it regulates maturation of immune synapses (Kumari et al., 2012), de-adhesion from intercellular adhesion molecule-1 (ICAM-1) (Morin et al., 2008), and interstitial migration and lymph node retention (Jacobelli et al., 2010). *In vitro* studies using primary B cells or B cell lines treated with blebbistatin, an inhibitor of class II myosin proteins, revealed a role for myosin IIa in B cell antigen extraction from membrane substrates (Natkanski et al., 2013) and antigen presentation to T cells (Vascotto et al., 2007). However, the role of myosin IIa in B cell functions *in vivo* has not been investigated.

Here, using mice in which myosin IIa was conditionally or inducibly deleted from B cells, we show that myosin IIa is required for B cell development at the pro-B cell stage. Moreover, when we deleted myosin IIa in more mature B cells, development and maintenance of splenic MZ, peritoneal B1b, and steady-state germinal center (GC) B cells was disturbed. Myosin IIa-deficient follicular B cells developed normally; however, these cells acquired an activated phenotype. Culturing myosin IIa-deficient B cells in the presence of various activating stimuli revealed a defect in cytokinesis. In addition, myosin IIa-deficient B cells showed impaired migration and were less efficient in internalizing membrane-tethered antigen, whereas internalization of soluble antigen was unperturbed. We also observed reduced acquisition of antigen from FDCs *in vivo*. Collectively, these defects resulted in reduced steady-state serum antibody levels and diminished antibody responses *in vivo*.

## Results

### Myosin IIa Is Required for Bone Marrow B Cell Development

**Figure 1:**
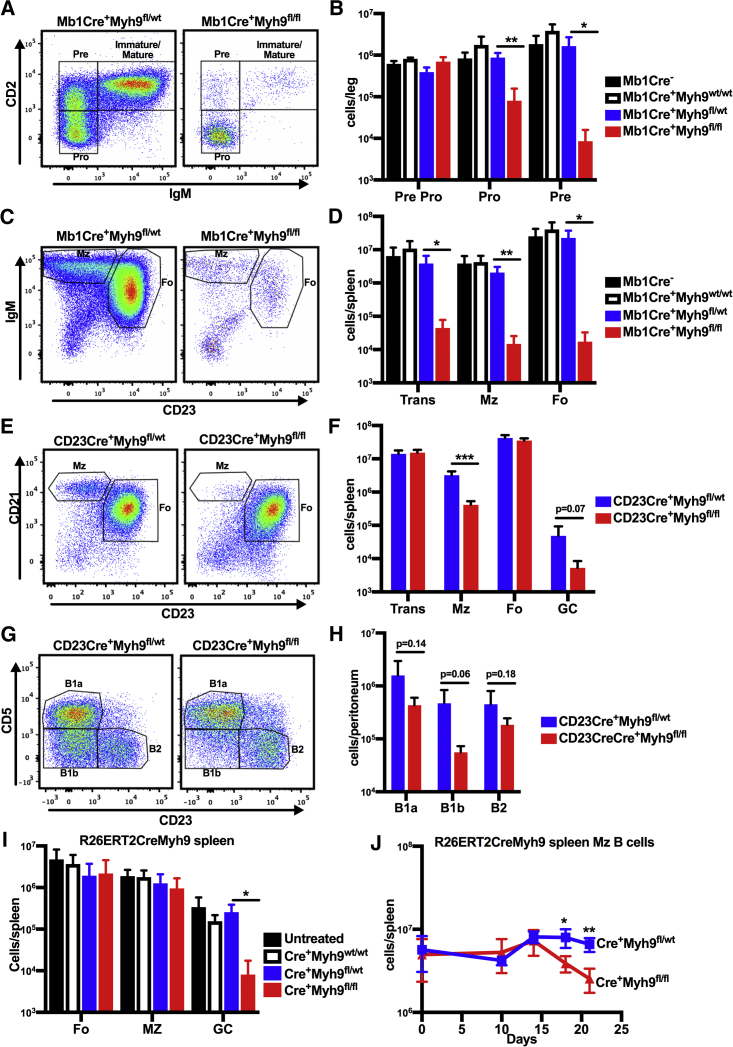
Disturbed B Cell Development and Maintenance after B Cell-Specific Deletion of Myosin IIa (A) Flow cytometric analysis of B220^+^CD19^+^ cells in the bone marrow (BM) of Mb1Cre^+^Myh9^wt/fl^ and Mb1Cre^+^Myh9^fl/fl^ mice. (B) Quantification of precursor B cell subsets in the BM of Mb1Cre^+^Myh9^wt/wt^ (n = 3), Mb1Cre^+^Myh9^wt/fl^ (n = 4), Mb1Cre^+^Myh9^fl/fl^ (n = 4), and Cre-negative littermates (n = 4). (C) Flow cytometric analysis of B220^+^AA4.1^−^ cells in spleens of Mb1Cre^+^Myh9^wt/fl^ and Mb1Cre^+^Myh9^fl/fl^ mice. (D) Quantification of B cell subsets in spleens of Mb1Cre^+^Myh9^wt/wt^ (n = 3), Mb1Cre^+^Myh9^wt/fl^ (n = 4), Mb1Cre^+^Myh9^fl/fl^ (n = 4), and Cre-negative littermates (n = 4). (E) Flow cytometric analysis of B220^+^AA4.1^−^ cells in spleens of CD23Cre^+^Myh9^wt/fl^ and CD23Cre^+^Myh9^fl/fl^ mice. (F) Quantification of B cell subsets in spleens of CD23Cre^+^Myh9^wt/fl^ (n = 6) and CD23Cre^+^Myh9^fl/fl^ (n = 4) mice. (G) Flow cytometric analysis of B220^+^ cells in the peritoneal cavity of CD23Cre^+^Myh9^wt/fl^ and CD23Cre^+^Myh9^fl/fl^ mice. (H) Quantification of B cell subsets in the peritoneal cavity of CD23Cre^+^Myh9^wt/fl^ (n = 6) and CD23Cre^+^Myh9^fl/fl^ (n = 4) mice. (I) Quantification of B cell subsets 14 days after the start of tamoxifen treatment in spleens of R26ERT2Cre^+^Myh9^wt/wt^, R26ERT2Cre^+^Myh9^wt/fl^, R26ERT2Cre^+^Myh9^fl/fl^, and Cre-negative mixed BM chimeras (n = 5). (J) Quantification of MZ B cell numbers in spleens of R26ERT2Cre^+^Myh9^wt/fl^ and R26ERT2Cre^+^Myh9^fl/fl^ mice over time since the start of tamoxifen treatment. Plotted are means ± SD per mouse. ^∗^p < 0.05, ^∗∗^p < 0.01, ^∗∗∗^p < 0.001 (unpaired t test). Trans, transitional B cells; MZ, marginal zone B cells; Fo, follicular B cells; GC, B220^+^Fas^+^CD38^−^ germinal center B cells. See also Figures S1 and S2.

Germline knockout of *Myh9*, encoding the myosin IIa heavy chain, leads to embryonic death (Conti et al., 2004). To study the role of myosin IIa in B cells, we crossed Myh9^fl/fl^ mice, in which exon 3 of *Myh9* is flanked by LoxP sites (Jacobelli et al., 2010), with Cd79aCre (Mb1Cre) and Fcer2Cre (CD23Cre) mice, resulting in mice in which *Myh9* is conditionally deleted from early bone marrow (BM) B cell precursors and more mature splenic transitional B cells, respectively (Hobeika et al., 2006, Kwon et al., 2008). Flow cytometric analysis of the BM and peripheral lymphoid organs of Mb1Cre^+^Myh9^fl/fl^ mice revealed severely reduced numbers of pro-B cells and in all subsequent stages of B cell development compared to Mb1Cre^+^Myh9^wt/fl^, Mb1Cre^+^Myh9^wt/wt^, or Cre-negative littermates (Figures 1A–1D), demonstrating that B cell development is blocked immediately after first expression of *Cd79a*. No differences were found in B cell development or mature B cell numbers between haploinsufficient and myosin IIa-wild-type mice, suggesting that a partial reduction of myosin IIa levels does not impair B cell development or maintenance of mature B cells. We conclude that myosin IIa is essential for early steps of B cell development.

### Myosin IIa Regulates Development and Maintenance of Splenic MZ and Peritoneal B1b B Cells

To investigate the role of myosin IIa in mature B cells, we analyzed CD23Cre^+^Myh9^fl/fl^ mice and found that total mature B cell numbers in the spleen, BM, and lymph nodes (LNs) were normal (Figures 1E, 1F, S1A, and S1B). However, splenic MZ and steady-state GC B cell numbers were reduced (Figures 1E and 1F). In the peritoneal cavity, the numbers of B1b B cells were also reduced, whereas B1a and B2 numbers were similar as in control mice (Figures 1G and 1H). Deletion of myosin IIa in follicular B cells was confirmed by analyzing mRNA expression of *Myh9* exon 3 and myosin IIa protein expression by western blot (Figures S2A and S2B). In addition, reduced myosin IIa protein levels were detected by flow cytometry in splenic CD23-expressing T2 cell and follicular B cell subsets of CD23Cre^+^Myh9^fl/fl^ mice, whereas CD23-negative T1 cells expressed normal levels (Figure S2C). In the peritoneal cavity, we observed reduced myosin IIa protein levels in B1b and B2 cells. However, B1a B cells retained myosin IIa expression (Figure S2D), most likely because these cells derive from fetal liver cells that do not express CD23.

The loss of MZ B cells was B cell intrinsic, because it was recapitulated when BM of CD23Cre^+^Myh9^fl/fl^ was mixed with 4 volumes of CD45.1 BM and transferred into sub-lethally irradiated *Rag1*-knockout (KO) mice (Figure S1C). Competition with wild-type B cells also reduced the numbers of myosin IIa-deficient follicular B cells, although not as severely as the number of MZ B cells.

Splenic MZ and peritoneal B1 B cells may share developmental and maintenance cues (Niiro and Clark, 2002). To investigate whether myosin IIa plays a role in the development or maintenance of MZ B cells, we mixed BM cells from R26ERT2Cre^+^Myh9^fl/fl^ and muMT mice and transferred them into sub-lethally irradiated *Rag1*-KO mice. In the resulting mice, myosin IIa can be acutely deleted specifically in B cells by administration of tamoxifen. Reduced levels of myosin IIa protein were detected in peripheral blood B cells by day 8 after the start of tamoxifen treatment, with maximally reduced levels from day 14 onward (Figure S2E). On day 14 after starting tamoxifen administration, MZ B cell numbers were not significantly different among R26ERT2Cre^+^Myh9^wt/wt^, R26ERT2Cre^+^Myh9^wt/fl^, and R26ERT2Cre^+^Myh9^fl/fl^ chimeras (Figure 1I). However, R26ERT2Cre^+^Myh9^fl/fl^ MZ B cell numbers started to decline 18 days after the start of tamoxifen treatment (Figure 1J). In contrast, steady-state R26ERT2Cre^+^Myh9^fl/fl^ GC B cell numbers were already reduced at day 14 (Figure 1I), indicating that myosin IIa is required for acute maintenance of GC B cells, but not MZ B cells. However, myosin IIa is important for MZ B cell development and long-term maintenance.

Development and maintenance of MZ B cells requires correct localization to the MZ (Lu and Cyster, 2002). To investigate localization of myosin IIa-deficient MZ B cells, we intravenously (i.v.) injected R26ERT2Cre^+^Myh9^fl/fl^ BM chimeras with an anti-Cd19-phycoerythrin (PE) antibody to label cells exposed to blood 14 days after the start of tamoxifen treatment. At this time point, myosin IIa is deleted, but MZ B cell numbers have not yet declined. Five minutes after injection of anti-Cd19-PE antibody, mice were culled and binding of antibody was analyzed by flow cytometry. Labeling of MZ B cells was slightly increased in R26ERT2Cre^+^Myh9^fl/fl^ chimeras (Figure S1D), indicating that myosin IIa-deficient MZ B cells were localized to the MZ or red pulp before their disappearance.

### Myosin IIa-Deficient Follicular B Cells Display Elevated Surface Activation Markers

**Figure 2:**
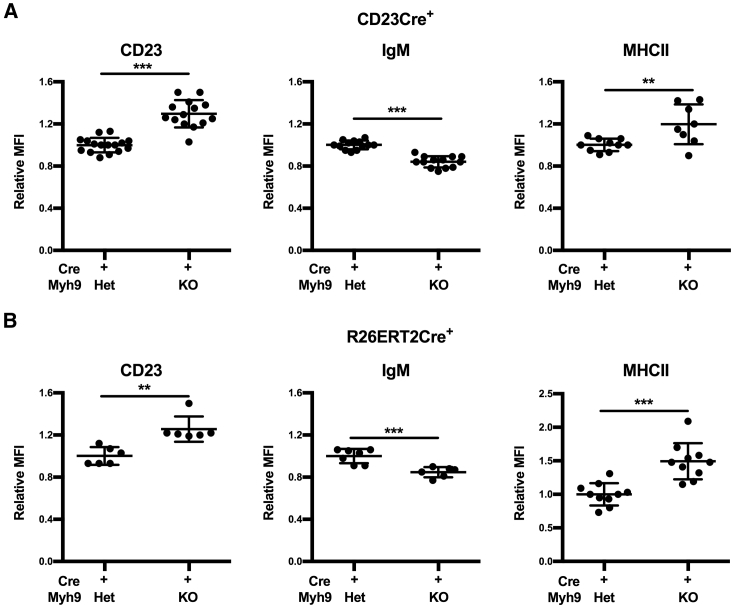
Myosin IIa-Deficient Follicular B Cells Express Altered Levels of Surface Activation Markers (A) Surface expression of CD23, IgM, and MHC class II on follicular B cells of CD23Cre^+^Myh9^wt/fl^ and CD23Cre^+^Myh9^fl/fl^ mice. (B) Relative expression of surface CD23, IgM, and MHC class II on follicular B cells of R26ERT2Cre^+^Myh9^wt/fl^ and R26ERT2Cre^+^Myh9^fl/fl^ mice 18 days after the start of tamoxifen treatment. Each dot represents one mouse. Horizontal bars reflect mean ± SD. Data are normalized on expression levels of Cre^+^Myh9^wt/fl^ littermate follicular B cells. ^∗∗^p < 0.01, ^∗∗∗^p < 0.001 (unpaired t test). See also Figure S3.

Although follicular B cells in CD23Cre^+^Myh9^fl/fl^ mice developed in normal numbers, they expressed higher levels of surface Fcer2 (CD23) and MHC class II and reduced levels of surface immunoglobulin M (IgM) (Figure 2A), indicating an activated phenotype. A similar follicular B cell surface marker phenotype was induced by acute depletion of myosin IIa in R26ERT2Cre^+^Myh9^fl/fl^ mixed BM chimeras (Figure 2B), suggesting that myosin IIa is continuously required to maintain a resting surface marker phenotype of follicular B cells. The surface marker phenotype was B cell intrinsic, because it was also observed in myosin IIa-deficient follicular B cells of 20% CD23Cre^+^Myh9^fl/fl^, 80% CD45.1 mixed BM chimeras (Figure S3A). However, we could not detect significant changes in expression of other activation markers, such as Cd44, Cd69, and Cd86 (Figure S3B). No differences were found in surface marker expression of B cells with wild-type and haplosufficient myosin IIa levels (data not shown), indicating that a partial reduction of myosin IIa levels has no effect on the surface marker phenotype.

To find clues of the signaling pathways that drive these phenotypic changes, we sorted follicular B cells of CD23Cre^+^Myh9^wt/fl^ and CD23Cre^+^Myh9^fl/fl^ mice by flow cytometry and analyzed gene expression by RNA sequencing. In myosin IIa-deficient B cells, 8 genes were significantly upregulated and 32 genes were significantly downregulated compared to haploinsufficient B cells (Table S1). The downregulated genes included the putative p53 target genes *Dusp1*, *Ets2*, *S100a9*, and *Zpf36l2*, in line with a report that myosin IIa post-transcriptionally stabilizes p53 (Schramek et al., 2014). However, the RNA sequencing (RNA-seq) data did not reveal clues as to what signaling pathways may be dysregulated in myosin IIa-deficient B cells.

MZ B cell development requires Adam10-mediated Notch2 cleavage and signaling (Gibb et al., 2010, Hammad et al., 2017), an event that might depend on myosin IIa-mediated forces or tension. To interrogate the role of myosin IIa in Adam10 translocation to the plasma membrane, we stimulated myosin IIa-deficient B cells with soluble anti-IgM and analyzed Adam10 surface expression by flow cytometry. A similar increase in Adam10 surface expression was detected in myosin IIa-deficient and myosin IIa-proficient B cells (Figures S4A and S4B). In agreement, myosin IIa-deficient B cells normally upregulated the Notch2 target genes *Deltex1*, *Hes1*, and *Hes5* when cultured on OP9 cells expressing the Notch ligand Dll1 (Figure S4C). We conclude that myosin IIa is not involved in Notch2 signaling.

### BCR Signaling and Internalization of Soluble Antigen Are Normal in Myosin IIa-Deficient B Cells

A lack of MZ B cell development, upregulation of CD23 and MHC class II, and decreased surface IgM expression have been associated with increased BCR signaling (Goodnow et al., 1988, Pillai and Cariappa, 2009). Thus, we hypothesized that myosin IIa is a negative regulator of BCR signaling. To study the role of myosin IIa in the regulation of BCR signaling, we stimulated myosin IIa-proficient and myosin IIa-deficient B cells with soluble anti-IgM and found that phosphorylation of Syk, Blnk, and Akt and intracellular calcium fluxes were similar (Figures S5A and S5B), suggesting proximal BCR signaling is unaffected by myosin IIa-deletion. In addition, prolonged stimulation of myosin IIa-deficient cells with soluble BCR ligands resulted in normal upregulation of the activation markers Cd69, Cd86, and MHC class II, albeit to slightly lower levels than in CD23Cre^+^Myh9^wt/fl^ cells (Figure S5C), suggesting myosin IIa is also not involved in regulating more distal BCR signaling pathways. Next, we analyzed internalization and processing of soluble antigen using DNA-based antigen degradation sensors as described previously (Nowosad et al., 2016). Myosin IIa-deficient B cells were as efficient in internalizing soluble anti-immunoglobulin κ (Igκ) as myosin IIa-proficient cells (Figure S5D), in agreement with previous reports for B cells treated with blebbistatin (Natkanski et al., 2013). Blebbistatin has also been reported to reduce antigen presentation capability of primary B cells and B cell lymphoma cell lines (Vascotto et al., 2007). However, similar levels of MHC class II-bound Ea peptide were detected at the cell surface of CD23Cre^+^Myh9^wt/fl^ and CD23Cre^+^Myh9^fl/fl^ B cells after stimulation with anti-Igκ and Ea peptide-loaded microbeads (Figure S5E). Altogether, these findings indicate that myosin IIa is not required for the regulation of BCR signaling or for internalization, processing, and presentation of soluble antigen.

### Myosin IIa-Deficient B Cells Are Less Efficient at Extracting Antigen from Membrane Substrates

**Figure 3:**
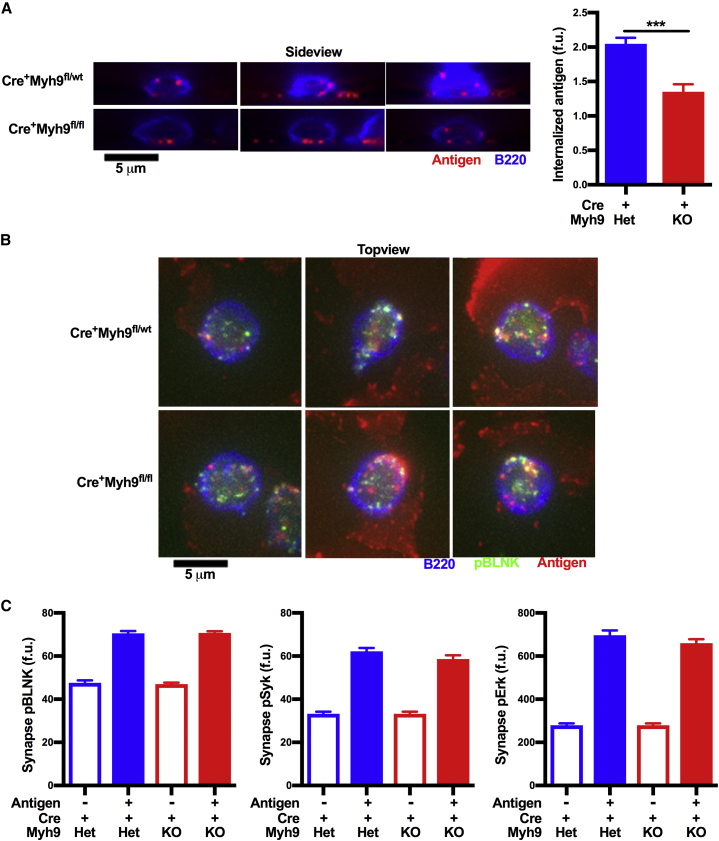
Myosin IIa-Deficient B Cells Are Less Efficient at Internalizing Membrane-Bound Antigen *In Vitro* (A) Side view reconstructions and quantification of internalized antigen of follicular B cells (blue) from CD23Cre^+^Myh9^wt/fl^ and CD23Cre^+^Myh9^fl/fl^ mice after 40 min of interaction with plasma membrane sheet (PMS)-bound antigen (red). (B) Top view of synapse plane showing pBlnk staining (green) in follicular B cells (blue) from CD23Cre^+^Myh9^wt/fl^ and CD23Cre^+^Myh9^fl/fl^ mice after 15 min of interaction with PMS-bound antigen (red). (C) Quantification of synaptic pBlnk (left), pSyk (middle), and pErk (right) after 20, 20, and 15 min of interaction with PMS, respectively. Cells that landed outside of PMS were used as unstimulated controls. Mean ± SEM (n > 192 cells). ^∗∗∗^p < 0.001 (Mann-Whitney U test). Fu, fluorescence units. See also Figure S5.

To investigate the role of myosin IIa in BCR signaling and internalization in response to membrane-presented antigen, we made use of a large-scale imaging approach of primary B cells on antigen-presenting plasma membrane sheets (PMSs) as developed previously (Nowosad et al., 2016). PMSs are flexible membrane substrates that facilitate B cell synapse formation, BCR signaling, and antigen internalization when coated with antigen. After 40 min on anti-Igκ-coated PMSs, myosin IIa-deficient B cells showed significantly less, but not absent, antigen internalization compared to myosin IIa-proficient cells (Figure 3A), showing that myosin IIa is important for efficient acquisition of membrane-bound antigen. To investigate whether the reduced internalization of membrane-bound antigen affects termination of BCR signaling, phosphorylation of BCR signaling pathway components was analyzed in myosin IIa-deficient B cells engaging antigen on PMS. However, we did not observe significant changes in phosphorylation of Blnk, Syk, or Erk (Figures 3B and 3C). Thus, myosin IIa does not regulate BCR signaling under these conditions.

### Myosin IIa Is Required for Efficient Migration and *In Vivo* Trafficking of B Cells

Adhesion to ICAM-1 lowers the threshold for B cell activation by promoting synapse formation (Carrasco et al., 2004). To study the adhesive properties of myosin IIa-deficient B cells, we stimulated cells with anti-IgM in the presence of soluble ICAM-1 and analyzed ICAM-1 binding to the cell surface by flow cytometry. In both CD23Cre^+^Myh9^fl/fl^ and CD23Cre^+^Myh9^fl/wt^ B cells, binding of ICAM-1 was induced to a similar extent (Figure S6A). Adhesion of anti-IgM or MnCl_2_-stimulated B cells to immobilized ICAM-1 was also similar between myosin IIa-deficient and myosin IIa-proficient B cells (Figure S6B).

**Figure 4:**
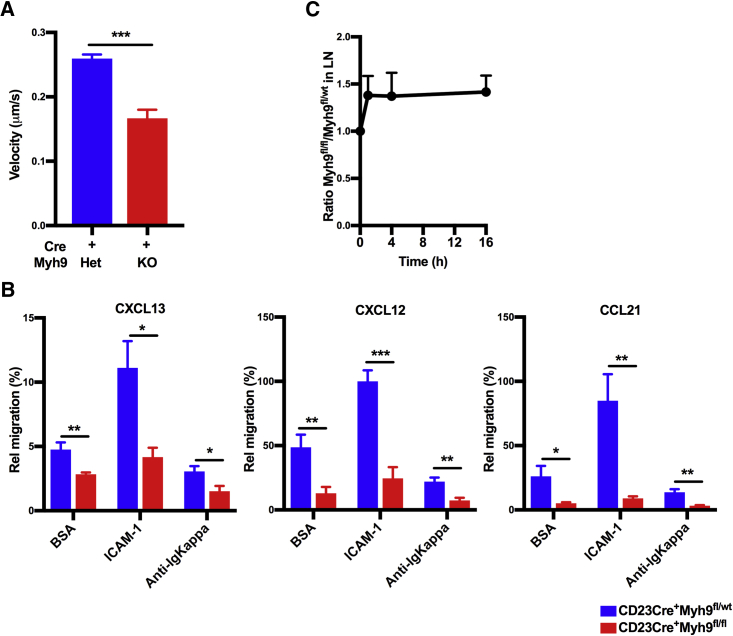
Disturbed Migration and *In Vivo* Trafficking of Myosin IIa-Deficient B Cells (A) Velocity of CXCL13-activated CD23Cre^+^Myh9^wt/fl^ and CD23Cre^+^Myh9^fl/fl^ B cells on an ICAM-1-coated glass surface (n > 48 cells). ^∗∗∗^p < 0.001 (Mann-Whitney U test). (B) Directional migration through BSA, ICAM-1, and anti-Igκ-coated Transwells of CD23Cre^+^Myh9^wt/fl^ and CD23Cre^+^Myh9^fl/fl^ B cells toward the indicated chemokines. Data are normalized on Cxcl12-mediated migration of CD23Cre^+^Myh9^wt/fl^ B cells. Mean ± SEM. ^∗^p < 0.05, ^∗∗^p < 0.01, ^∗∗∗^p < 0.001 (unpaired t test). (C) Ratio of B cells from CD23Cre^+^Myh9^wt/fl^ and CD23Cre^+^Myh9^fl/fl^ mice after i.v. injection into C57BL/6J mice normalized to input ratio (n = 8 mice). Mean ± SD. LN, lymph node. See also Figure S6.

Using time-lapse imaging, we analyzed motility of myosin IIa-deficient B cells on ICAM-1-coated glass in the presence of Cxcl13 and observed reduced crawling speed compared to CD23Cre^+^Myh9^fl/wt^ B cells (Figure 4A; Video S1). A fraction of myosin IIa-deficient cells developed elongated uropods (Video S1). In Transwell assays, myosin IIa-deficient B cells displayed reduced migration toward Cxcl13, Cxcl12, and Ccl21 (Figure 4B). Coating of Transwell membranes with ICAM-1 facilitated robust migration of myosin IIa-proficient B cells, but not of myosin IIa-deficient B cells. In contrast, coating of Transwell membranes with anti-Igκ, a stronger adhesive, severely reduced migration of CD23Cre^+^Myh9^fl/wt^ and nearly abolished migration of CD23Cre^+^Myh9^fl/fl^ B cells. Altogether, these data indicate that B cells require myosin IIa for detachment from adhesive surfaces.

Video S1. Myosin IIa Is Required for Efficient Chemokinesis, Related to Figure 4 CD23Cre^+^Myh9^fl/wt^ (left) and CD23Cre^+^Myh9^fl/fl^ (right) B cells migrating on ICAM-1-coated glass slides in the presence of 1 μg/mL of Cxcl13.

To analyze migration of myosin IIa-deficient B cells *in vivo*, we i.v. injected labeled CD23Cre^+^Myh9^fl/fl^ and CD23Cre^+^Myh9^fl/wt^ B cells into C57BL/6J mice and found an increased ratio of myosin IIa-deficient cells in the LNs after 1, 4, and 16 hr (Figure 4C). This skewed ratio of B cells in the LN was not due to facilitated homing of myosin IIa-deficient cells caused by increased L-selectin (CD62L) expression, because we observed normal CD62L expression on myosin IIa-deficient B cells (Figure S6C). To analyze whether myosin IIa-deficient cells migrate normally within LNs, we assessed the ratio of myosin IIa-deficient and myosin IIa-proficient B cells by immunofluorescence staining of cryopreserved LNs 16 hr after transfer and found that the ratio of myosin IIa-proficient and myosin IIa-deficient cells in B cell follicles was similar to the ratio determined by flow cytometry (Figure S6D).

### Myosin IIa Is Required for Efficient Acquisition of Antigen *In Vivo*

To investigate the role of myosin IIa in antigen acquisition *in vivo*, CD23Cre^+^Myh9^fl/wt^ and CD23Cre^+^Myh9^fl/fl^ mice were crossed with SW_HEL_ mice. B cells of SW_HEL_ mice express a high-affinity anti-hen egg lysozyme (HEL) antibody as a BCR on the cell surface (Phan et al., 2003). In the resulting mice, approximately half of the B cells expressed the HEL-specific BCR, as determined by binding of biotinylated HEL and PE-labeled streptavidin (Figure S7A). A similar percentage of cells could be stained with HEL3x, a modified version of HEL with 10,000-fold lower affinity for the SW_HEL_ BCR (Paus et al., 2006), albeit with lower mean fluorescence intensity (MFI). No difference was found in HEL binding capacity between antigen-specific B cells of CD23Cre^+^Myh9^fl/wt^SW_HEL_ and CD23Cre^+^Myh9^fl/fl^SW_HEL_ mice despite lower surface IgM levels in the latter (Figure S7B), suggesting total surface BCR levels, which also include immunoglobulin D (IgD), were not significantly changed.

To target HEL antigen to FDCs *in vivo*, we adapted a protocol to generate PE-labeled HEL immune complexes in CD45.1 mice by intraperitoneal injection of anti-PE antibody, followed 1 day later by subcutaneous (s.c.) injection of HEL or HEL3x bound to PE-labeled streptavidin near the axillary and inguinal LNs (Phan et al., 2007, Suzuki et al., 2009). Twenty-four hours after generation of immune complexes, when most of the antigen is presented on the surface of FDCs in LNs (Figure S7C), SW_HEL_ B cells were transferred. Fourteen hours after transfer, we isolated B cells from axillary and inguinal LNs and could detect PE-containing immune complexes on approximately 60% of transferred B cells that express the SW_HEL_ BCR. In contrast, only 3% of donor B cells that do not express a HEL-specific BCR were positive for PE (Figure S7D), possibly because of capture of anti-PE-HEL-streptavidin-PE complexes via complement receptor Cr2 (Phan et al., 2007). PE^+^ B cells had upregulated the activation markers Cd69 and Cd86, demonstrating that the HEL uptake is BCR mediated (Figure S7E). Omitting passive immunization with anti-PE antibody resulted in few PE-positive cells, suggesting that the antigen has been taken up from immune complex binding APCs.

**Figure 5:**
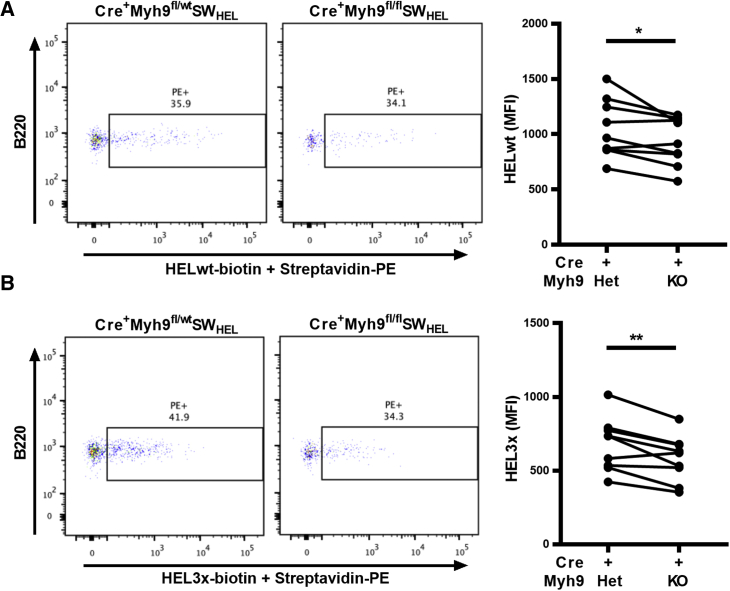
Myosin IIa Is Required for Efficient Acquisition of Low-Affinity Antigen *In Vivo* (A) Flow cytometric analysis and quantification of HEL-streptavidin-PE complex capture by B220^+^ cells from CD23Cre^+^Myh9^fl/wt^SW_HEL_ or CD23Cre^+^Myh9^fl/fl^SW_HEL_ mice 14 hr after transfer into HEL-streptavidin-PE-immunized CD45.1 mice. Plotted is the MFI of PE^+^ cells, paired per mouse. (B) Flow cytometric analysis and quantification of HEL3x-streptavidin-PE complex capture by B220^+^ cells from CD23Cre^+^Myh9^fl/wt^SW_HEL_ or CD23Cre^+^Myh9^fl/fl^SW_HEL_ mice 14 hr after transfer into HEL3x-streptavidin-PE-immunized CD45.1 mice. Plotted is the MFI of PE^+^ cells, paired per mouse. Flow cytometry plots display data of a representative experiment. ^∗^p < 0.05, ^∗∗^p < 0.01 (paired t test). See also Figure S7.

To quantify HEL uptake from APCs *in vivo* in the absence of myosin IIa, B cells from CD23Cre^+^Myh9^fl/wt^SW_HEL_ and CD23Cre^+^Myh9^fl/fl^SW_HEL_ were isolated, labeled, and transferred concomitantly into HEL-immunized mice. After 14 hr, we found a modest but significant reduction in HEL bound to myosin IIa-deficient SW_HEL_ B cells (Figure 5A). A similar reduction in antigen uptake by myosin IIa-deficient SW_HEL_ B cells was observed when the lower-affinity HEL3x was used (Figure 5B). HEL-positive CD23Cre^+^Myh9^fl/wt^SW_HEL_ and CD23Cre^+^Myh9^fl/fl^SW_HEL_ B cells equally upregulated Cd69 and Cd86 (Figure S7E), demonstrating that access to antigen is similar. We conclude that myosin IIa is a positive regulator of antigen acquisition of membrane-bound antigen *in vivo*.

### Myosin IIa-Deficient B Cells Have a Defect in Cytokinesis

**Figure 6:**
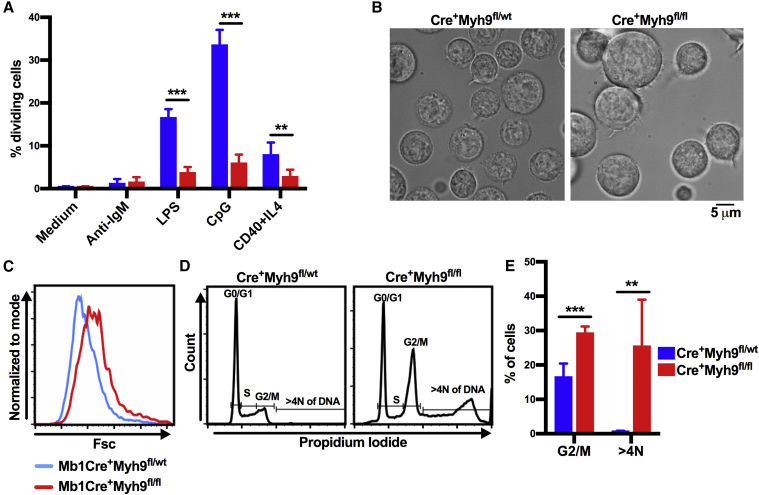
Myosin IIa-Deficient B Cells Have a Defect in Cytokinesis (A) Percentage of CD23Cre^+^Myh9^wt/fl^ and CD23Cre^+^Myh9^fl/fl^ B cells that have divided after 48 hr of cell culture in the presence of indicated stimuli. (B) Light microscopy images of CD23Cre^+^Myh9^wt/fl^ and CD23Cre^+^Myh9^fl/fl^ B cells stimulated with CpG for 48 hr. (C) Size of BM B220^+^CD19^+^CD2^−^IgM^−^ pro-B cells of Mb1Cre^+^Myh9^wt/fl^ and Mb1Cre^+^Myh9^fl/fl^ mice. (D) Flow cytometric analysis of propidium iodide incorporation of CD23Cre^+^Myh9^wt/fl^ and CD23Cre^+^Myh9^fl/fl^ B cells stimulated with CpG for 48 hr. (E) Quantification of cells in G2/M phase and cells with more than 4N of DNA after 48 hr of CpG stimulation. Means ± SD. ^∗∗^p < 0.01, ^∗∗∗^p < 0.001 (unpaired t test).

Past experiments with blebbistatin in HeLa and Cos-7 cells demonstrated that class II myosins cooperate to separate daughter cells during cytokinesis, the final step of the cell cycle (Ma et al., 2012, Straight et al., 2003). Because myosin IIa is the only class II myosin expressed in lymphocytes and both the pro-B cell stage and the GC are sites of extensive B cell proliferation, we hypothesized that myosin IIa-deficient B cells may have a defect in cytokinesis. To investigate proliferation of myosin IIa-deficient B cells, cells were labeled and cultured in the presence of various stimuli. After 48 hr, up to 40% of CD23Cre^+^Myh9^fl/wt^ follicular B cells stimulated with lipopolysaccharide (LPS), CpG, or Cd40lg and interleukin-4 (IL-4) had divided, as determined by dilution of dye (Figure 6A). In contrast, less than 10% of CD23Cre^+^Myh9^fl/fl^ B cells had proliferated regardless of the stimulus. CpG-stimulated myosin IIa-deficient B cells were markedly enlarged (Figure 6B), in agreement with a defect in cytokinesis. Pro-B cells of Mb1Cre^+^Myh9^fl/fl^ mice were larger than myosin IIa-proficient counterparts in Mb1Cre^+^Myh9^fl/wt^ mice (Figure 6C), suggesting these cells may have failed cytokinesis *in vivo*.

When stimulated with CpG for 48 hr *in vitro*, a higher proportion of myosin IIa-deficient B cells were in G2 or M phase, harboring 4N of DNA, as determined by propidium iodide incorporation, and indicating a block in a late phase of the cell cycle (Figures 6D and 6E). Moreover, CpG stimulation of myosin IIa-deficient B cells resulted in a large fraction of cells containing more than 4N of DNA, suggesting these cells failed to complete cytokinesis and entered the cell cycle again. Thus, myosin IIa is essential for B cell proliferation, explaining the block of B cell development and loss of GC cells *in vivo*.

### Myosin IIa Is Essential for Antibody Responses

**Figure 7:**
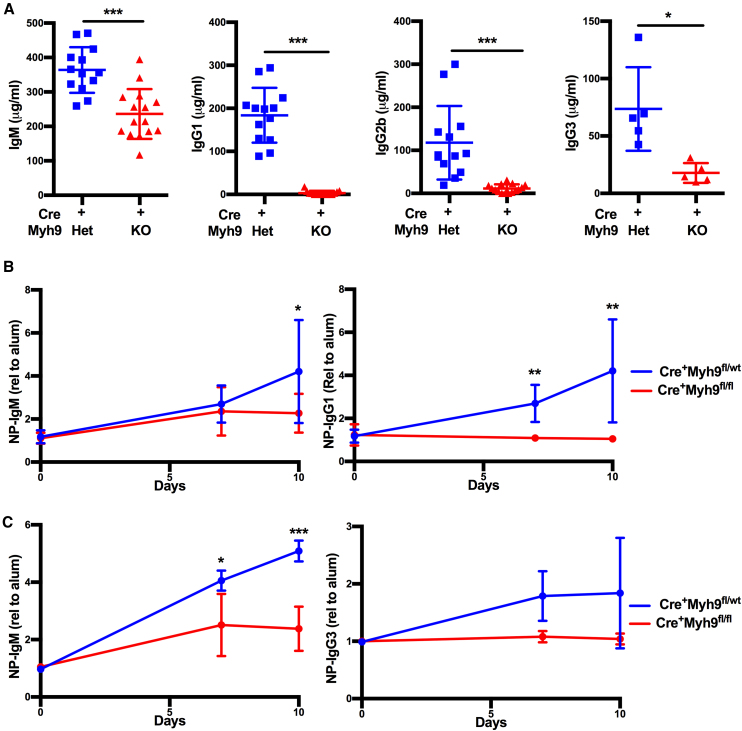
Myosin IIa Is Essential for Antibody Responses (A) Serum immunoglobulin levels of CD23Cre^+^Myh9^fl/wt^ and CD23Cre^+^Myh9^fl/fl^ mice. (B) NP-specific immunoglobulin levels of CD23Cre^+^Myh9^fl/wt^ and CD23Cre^+^Myh9^fl/fl^ BM-reconstituted mice after immunization with the T-dependent antigen NP-CGG (n = 10). (C) NP-specific immunoglobulin levels of CD23Cre^+^Myh9^fl/wt^ and CD23Cre^+^Myh9^fl/fl^ BM-reconstituted mice after immunization with the T-independent antigen NP-Ficoll (n = 5). Dots represent individual mice analyzed in two experiments. Horizontal bars reflect mean ± SD. ^∗^p < 0.05, ^∗∗^p < 0.01, ^∗∗∗^p < 0.001 (unpaired t test).

Collectively, the cytokinesis defect, the reduced levels of MZ and GC B cells, and the reduced capacity to acquire antigen could result in disrupted antibody production. CD23Cre^+^Myh9^fl/fl^ mice had diminished levels of steady-state serum IgM and immunoglobulin G (IgG) 1, IgG2b, and IgG3 (Figure 7A). To study the role of myosin IIa in antibody responses, we transferred BM from CD23Cre^+^Myh9^fl/wt^ and CD23Cre^+^Myh9^fl/fl^ mice into *Rag1*-KO mice. After reconstitution, mice were immunized with the T-dependent antigen 4-hydroxy-3-nitrophenylacetyl-chicken gamma globulin (NP-CGG), and NP-specific antibodies in the serum were analyzed after 7 and 10 days. NP-specific IgG1 antibodies were not detected in CD23Cre^+^Myh9^fl/fl^-reconstituted mice, whereas a clear induction of NP-specific IgG1 antibodies was found in CD23Cre^+^Myh9^fl/wt^-reconstituted mice (Figure 7B). Some NP-specific IgM could be detected in immunized CD23Cre^+^Myh9^fl/fl^-reconstituted mice, although at lower levels than in immunized CD23Cre^+^Myh9^fl/wt^-reconstituted mice. When immunized with the T-independent antigen NP-Ficoll, CD23Cre^+^Myh9^fl/fl^-reconstituted mice did not generate detectable levels of NP-specific IgG3 (Figure 7C). A small induction of NP-specific IgM could be detected but was significantly less than in CD23Cre^+^Myh9^fl/wt^-reconstituted mice. We conclude that myosin IIa is essential for mounting efficient B cell antibody responses.

## Discussion

B cell antigen acquisition from the surface of APCs is increasingly recognized as an important step in B cell responses (Cyster, 2010). Previous *in vitro* genetic and pharmacological perturbations identified myosin IIa contractility as an important factor in antigen extraction and delivery into MHC class II-containing processing compartments (Natkanski et al., 2013, Vascotto et al., 2007). Here, using mice in which myosin IIa was conditionally or inducibly deleted from mature B cells, we demonstrate that myosin IIa is important for antigen acquisition from FDCs *in vivo*. This is consistent with our discovery that FDCs have stiff membranes compared to other APCs, requiring application of strong forces by antigen-extracting B cells (Spillane and Tolar, 2017). We have also found that GC B cells have increased force generation capability, supporting that strong forces are required for antigen extraction in the GC (Nowosad et al., 2016). However, the rapid and complete disappearance of myosin IIa-deficient GC B cells, most likely due to a cytokinesis defect, prevented us from analyzing these cells in this study.

The extraction of membrane-bound antigen was only reduced by approximately 50% after genetic deletion of myosin IIa, in contrast to the 80%–90% reported with blebbistatin inhibition (Natkanski et al., 2013, Nowosad et al., 2016). This milder effect of myosin IIa deletion on antigen uptake prevented us from assessing the potential effects of cellular mechanics on antigen affinity discrimination in this model. It is possible that genetic deletion of myosin IIa can be compensated by other molecular motors or that blebbistatin has off-target effects that further diminish force generation by B cells. Differences between genetic inactivation of myosin IIa and inhibition by blebbistatin have also been observed when studying cytokinesis (Ma et al., 2012). Along these lines, we found that myosin IIa was not required for processing and presentation of soluble antigens, as reported previously (Vascotto et al., 2007). Possibly, alternative compensatory mechanisms take over this process after long-term genetic deletion of myosin IIa. This suggests caution is required when interpreting inhibitor or genetic studies in isolation.

When we analyzed antigen acquisition by myosin IIa-deficient B cells from APCs *in vivo*, we found that it was only modestly reduced, suggesting that substantial antigen extraction can be achieved without specialized myosin IIa-dependent pulling activity in the immune synapse. Again, deletion of myosin IIa may have been compensated by other molecular motors. In addition, the disturbed migration of myosin IIa-deficient B cells may facilitate antigen uptake *in vivo* due to prolonged interaction with the APC. Further studies on the role of cellular motility in B cell antigen acquisition should resolve this question.

Our analysis of myosin IIa-deficient B cells also shows that myosin IIa is important for the development of MZ and B1b B cells and negatively regulates the activation of follicular B cells. A loss of MZ B cells, combined with an activated phenotype of follicular B cells, has previously been reported in mice lacking negative regulators of BCR signaling, such as *Cd22* and *Aiolos* (Cariappa et al., 2001, Nitschke et al., 1997, Sato et al., 1996), and in transgenic mice expressing a self-reactive BCR (Cooke et al., 1994, Goodnow et al., 1988) or overexpressing *Cd19* (Tedder et al., 1997). This suggests that myosin IIa is a negative regulator of BCR signaling.

Sustained BCR signaling may render B cells anergic *in vivo* (Cooke et al., 1994, Goodnow et al., 1988). Although myosin IIa-deficient B cells responded normally to antigen stimulation, we observed a reduced capacity to upregulate Cd86, a feature previously attributed to anergic B cells (Benschop et al., 2001, Rathmell et al., 1998), suggesting myosin IIa-deficient B cells may have become partly anergic. Anergic B cells are short lived (Santulli-Marotto et al., 1998), which may explain the reduced numbers of follicular B cells in competitive BM chimeras. Hyper-responsiveness of follicular B cells has also been described in mice in which other components of the B cell cytoskeleton or BCR internalization or trafficking machinery were deleted, such as *Was*, *Dbnl*, or *Cbl*, often resulting in a mild autoimmune phenotype (Becker-Herman et al., 2011, Kitaura et al., 2007, Seeley-Fallen et al., 2014, Song et al., 2013). However, follicular B cells in these mice did not develop a hyperactivated surface phenotype, suggesting that myosin IIa may regulate B cell activation by a distinct mechanism. Possibly, myosin IIa may regulate BCR signaling under specific conditions, such as signaling induced by antigen presented on APCs or during a distinct developmental step. In support, transgenic expression of antigen on FDCs resulted in reduced IgM and increased MHC class II on antigen-specific naive follicular B cells (Yau et al., 2013), suggesting that BCR signaling induced by FDC-presented ligands in the spleen could lead to an activated phenotype similar to that observed in mice with B cell-specific deletion of myosin IIa. However, the exact nature of the signaling pathway activated in the myosin IIa-deficient B cells remains to be identified, because we did not observe an obvious increase in canonical BCR signaling induced by either soluble or membrane-bound antigens.

Analysis of adhesion *in vitro* showed that myosin IIa-deficient B cells adhered normally to soluble or immobilized ICAM-1. Moreover, when co-stimulated with both membrane-bound antigen and immobilized ICAM-1, BCR signaling was similar in myosin IIa-deficient and myosin IIa-proficient B cells (data not shown), demonstrating that initial attachment to ICAM-1 is normal. However, time-lapse imaging and Transwell experiments demonstrated that B cells require myosin IIa for detachment from adhesive substrates, similar to what has been described for myosin IIa-deficient T cells (Jacobelli et al., 2010, Morin et al., 2008). We speculate that this defect in detachment from adhesive substrates may result in prolonged interaction with antigen on APCs and thus in sustained BCR signaling *in vivo*. *Lsc*-deficient mice, which also have a defect in ICAM-1 detachment, harbor reduced MZ B cell numbers and follicular B cells with a hyperactivated phenotype (Girkontaite et al., 2001, Rubtsov et al., 2005), partly recapitulating the phenotype of mice with myosin IIa-deleted B cells described here.

Although the loss of MZ B cells after myosin IIa deletion is consistent with sustained BCR signaling, at least three other signals that are required for MZ B cell development could be potentially involved in this phenotype (Pillai and Cariappa, 2009). First, MZ B cell development needs Notch2 signaling. Interaction of Notch2 with its ligand Dll1 initiates cleavage of Notch2 by Adam10 (Gibb et al., 2010), an event that could rely on myosin IIa-mediated Adam10 transport, force generation, or cell tension. However, we measured normal Adam10 surface translocation after BCR stimulation and normal upregulation of Notch target genes in myosin IIa-deficient B cells, suggesting myosin IIa does not influence Notch signaling. Second, MZ B cell development depends on canonical nuclear factor κB (NF-κB) signaling, most likely induced by Tnfsf13b (BAFF). However, genetic disruption of canonical NF-κB signaling does not result in upregulation of activation markers. Moreover, *in vitro* survival in the presence of BAFF was normal in myosin IIa-deficient B cells (data not shown), suggesting myosin IIa is also not required for overall regulation of BAFF signaling. Finally, MZ B cell development and maintenance require migration and subsequent retention in the MZ (Lu and Cyster, 2002), which could depend on myosin IIa. However, we found increased labeling of myosin IIa-deficient MZ B cells after injection of anti-Cd19 antibody, indicating that more, not fewer, myosin IIa-deficient MZ B cells are localized in the MZ and red pulp. Possibly, a decrease in myosin IIa-dependent cellular locomotion may result in less shuttling of MZ B cells between the MZ and the follicle.

MZ B cells could also suffer from the observed cytokinesis defect, although proliferation of MZ B cells during steady-state conditions is generally very low and has, to our knowledge, only been reported when BM B cell development is blocked (Hao and Rajewsky, 2001). It is conceivable that declining numbers of MZ B cells at some point induce proliferation of remaining MZ B cells, thereby accelerating loss of MZ B cells through the defect in proliferation. However, loss of myosin IIa-deficient MZ B cells was also observed in the presence of wild-type MZ B cells in competitive BM chimeras, demonstrating that the initial loss of MZ B cells is cell intrinsic.

Overall, our data show that myosin IIa is required for efficient antibody responses, which is likely a result of the combined effects on antigen acquisition and proliferation. It is probable that because of these effects, myosin IIa-deficient B cells were unable to contribute to autoimmune reactions despite their activated phenotype, in contrast to the reported autoimmune syndromes in other mice with disturbed BCR internalization and trafficking (Becker-Herman et al., 2011, Kitaura et al., 2007, Song et al., 2013). Myosin IIa has been identified as a tumor suppressor by cytokinesis regulation or by post-transcriptional regulation of p53 (Conti et al., 2015, Schramek et al., 2014). We found several p53 target genes downregulated in myosin IIa-deficient follicular B cells, suggesting p53 function may also be deregulated in myosin IIa-deficient follicular B cells. However, whereas p53 null mice develop spontaneous lymphomas (Harvey et al., 1995), it is likely that transformation of myosin IIa-deficient B cells is again limited by the proliferation defect described in this paper.

Our data thus establish a critical role of myosin contractility in multiple aspects of B cell biology and should open new avenues to study the role of the cytoskeleton and B cell intrinsic force generation in antibody responses.

## Experimental Procedures

### Mice

Myh9^fl/fl^ (Myh9^tm5Rsad^) mice were crossed with Mb1Cre (Cd79a^tm1(cre)Reth^), CD23Cre (Tg(Fcer2a-cre)5Mbu), and R26ERT2Cre (Gt(ROSA)26Sor^tm1(cre/ERT2)Thl^) mice as described previously (de Luca et al., 2005, Hobeika et al., 2006, Jacobelli et al., 2010, Kwon et al., 2008). CD23CreMyh9^fl/fl^ mice were crossed with SW_HEL_ (Igh^tm1Rbr^-Tg(IgkHyHEL10)1Rbr) mice (Phan et al., 2003). 6–12 week old male and female mice were used for *ex vivo* analysis of cell populations. For *in vitro* experiments, cells isolated from 6–20 week old mice were used.

To generate BM transfer or chimera mice, *Rag1*-deficient mice (Rag1^tm1Mom^) were irradiated with 5 Gy and reconstituted with BM cells by i.v. injection. R26ERT2Cre^+^Myh9 mice received 2 mg/day of tamoxifen (Sigma) suspended in corn oil by intraperitoneal injection for 5 days. When indicated, BM was mixed with 80% muMT (Ighm^tm1Cgn^) or CD45.1 (B6.SJL-Ptprc^a^/Nimr). Mice were bred and kept in accordance with guidelines set by the UK Home Office and the Francis Crick Institute Ethical Review Panel.

### Large-Scale Imaging

Generation of PMS and the large-scale imaging approach have been described previously (Natkanski et al., 2013, Nowosad et al., 2016). A brief description is provided in Supplemental Experimental Procedures.

### Antigen Acquisition

To study *in vivo* antigen acquisition, we adapted a protocol to generate HEL-containing immune complexes as described previously (Phan et al., 2007). CD45.1 mice were passively immunized with a polyclonal anti-PE antibody (Rockland Immunochemicals). The next day, 10 μg of biotinylated HEL or HEL3x protein, produced in house as described (Paus et al., 2006), was complexed to PE-labeled streptavidin (BioLegend) and injected s.c. near the inguinal and axillary LNs under isoflurane anesthesia. The following day, CD23Cre^+^Myh9^fl/wt^SW_HEL_ and CD23Cre^+^Myh9^fl/fl^SW_HEL_ B cells were isolated, fluorescently labeled, mixed, and transferred to immunized recipient mice by i.v. injection. After 14 hr, inguinal and axillary LNs were harvested and analyzed by flow cytometry.

*In vitro* internalization and presentation of soluble antigen are described in Supplemental Experimental Procedures.

### Adhesion and Migration

To investigate *in vivo* homing and migration, B cells were labeled with 5-chloromethylfluorescein diacetate (CMFDA) and CellTrace Far Red (CTFR) and mixed in a 1:1 ratio, and 20 × 10^6^ cells were i.v. injected into C57BL/6J recipient mice. At indicated times, LNs were collected and the ratio of donor cells was determined by flow cytometry or immunofluorescence microscopy of cryosections.

To determine localization of myosin IIa-deficient MZ B cells, R26ERT2Cre^+^Myh9 muMT BM chimeras were treated with tamoxifen as described earlier. 14 days after the first tamoxifen injection, mice received 1 μg anti-Cd19-PE antibody by i.v. injection. Mice were culled 5 min later, splenocytes were harvested on ice, and cells were analyzed by flow cytometry. *In vitro* migration and adhesion experiments are described in Supplemental Experimental Procedures.

### Flow Cytometry, Signaling, Activation, and Proliferation

B cell populations, signaling, activation, proliferation, and gene expression were analyzed using standard techniques described in Supplemental Experimental Procedures.

### Statistical Analysis

Statistical analyses were performed using GraphPad Prism 7 software. For each experiment, the statistical test used, the sample size, and the statistical significance are included in the figure legend.

## Data and Software Availability

The accession number for the data from transcriptomic analysis reported in this paper is GEO: GSE113114.

## Supplemental Information

Document S1. Supplemental Experimental Procedures, Figures S1–S7, and Table S1

Document S2. Article plus Supplemental Information
