# Supplementary material for: Myosin IIa Promotes Antibody Responses by Regulating B Cell Activation, Acquisition of Antigen, and Proliferation
Source: Cell Rep. 2018 May 22;23(8):2342–53. doi: 10.1016/j.celrep.2018.04.087 (PMC5986709; doi:10.1016/j.celrep.2018.04.087)
Supplement: Document S1. Supplemental Experimental Procedures, Figures S1–S7, and Table S1 [file mmc1.pdf]

Cell Reports, Volume *23*

Supplemental Information

Myosin IIa Promotes Antibody Responses by Regulating B Cell Activation, Acquisition of Antigen, and Proliferation

Robbert Hoogeboom, Elizabeth M. Natkanski, Carla R. Nowosad, Dessislava Malinova, Rajesh P. Menon, Antonio Casal, and Pavel Tolar

Supplemental Figures

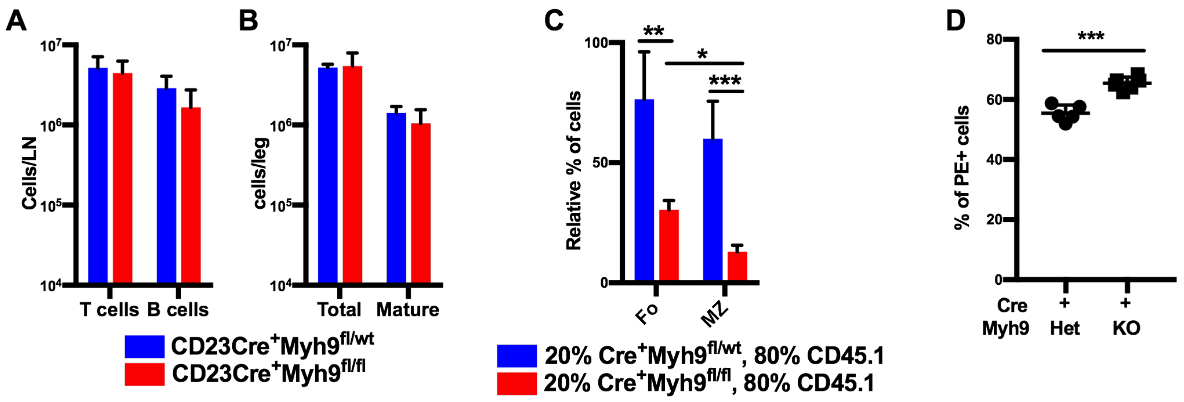

Figure S1. B cell numbers in secondary lymphoid organs of CD23Cre^{+}Myh9$^{fl/fl}$ mice, related to Figure 1.
(A) Quantification of lymph node (LN) T and B cells in CD23Cre^{+}Myh9$^{wt/fl}$ (n=6) and CD23Cre^{+}Myh9$^{fl/fl}$ (n=4) mice.
(B) Quantification of total and mature B cells in the bone marrow (BM) of CD23Cre^{+}Myh9$^{wt/fl}$ (n=6) and CD23Cre^{+}Myh9$^{fl/fl}$ (n=4) mice.
(C) Percentage of CD23Cre^{+}Myh9$^{wt/fl}$ and CD23Cre^{+}Myh9$^{fl/fl}$ Follicular (Fo) and Marginal Zone (MZ) B cells in competitive BM chimeras reconstituted in Rag1-KO mice (n=5 mice). Frequency normalized to percentage of CD23Cre^{+}Myh9$^{wt/fl}$ and CD23Cre^{+}Myh9$^{fl/fl}$-derived T cells.
(D) Percentage of labelled MZ B cells in tamoxifen treated R26ERT2Cre^{+}Myh9$^{wt/fl}$ and R26ERT2Cre^{+}Myh9$^{fl/fl}$ BM chimeras 5 min after injection with anti-CD19-PE antibody.
Mean ± SD. * P<0.05, ** P<0.01, *** P<0.001 (unpaired t-test)

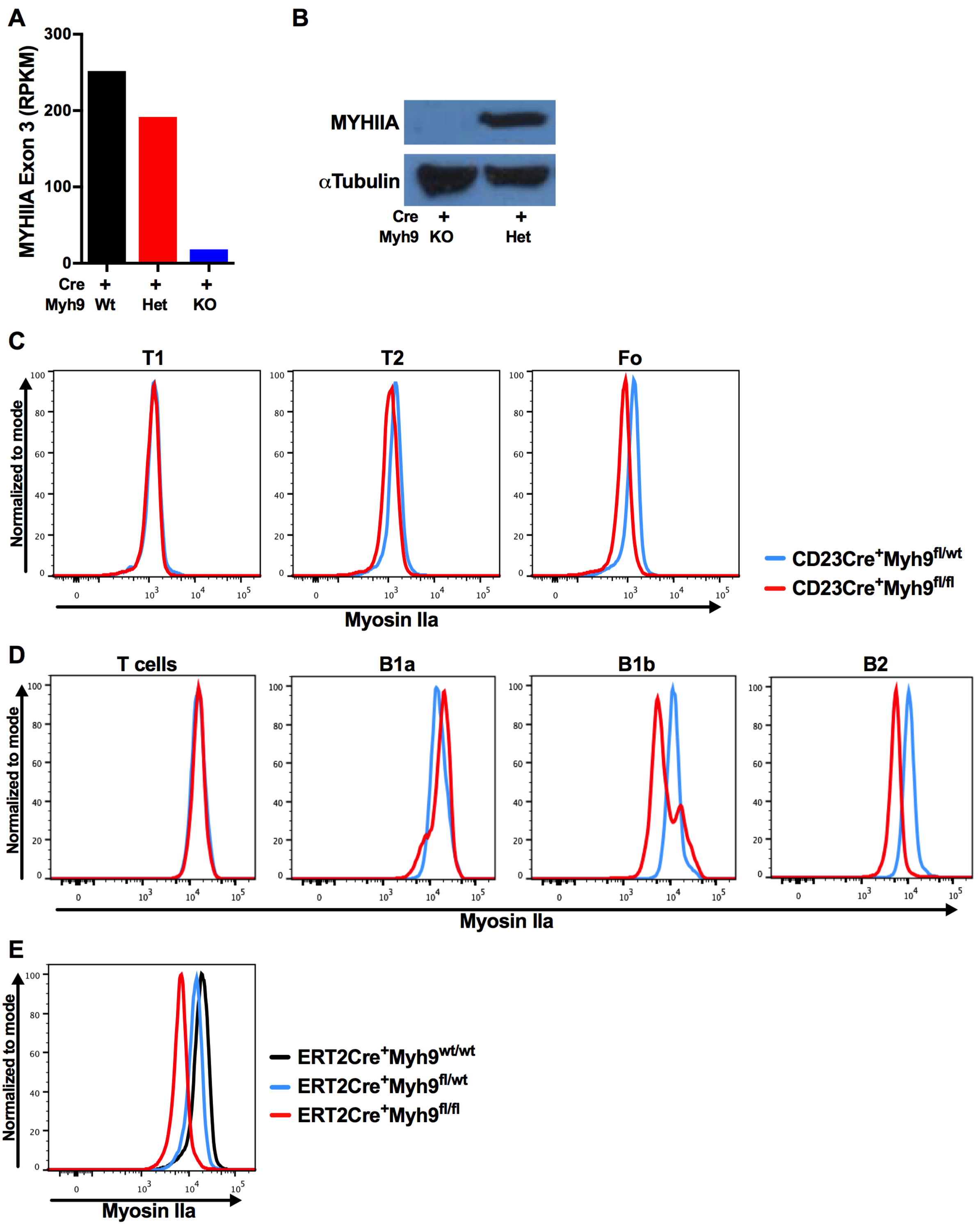

Figure S2. Myosin deletion in B cells of Mb1Cre^{+}Myh9$^{fl/fl}$, CD23Cre^{+}Myh9$^{fl/fl}$ and tamoxifen-treated R26ERT2Cre^{+}Myh9$^{fl/fl}$ mice, related to Figure 1.

(A) Myosin IIa mRNA expression levels of FACS-sorted follicular B cells from CD23Cre^{+}Myh9$^{wt/wt}$, CD23Cre^{+}Myh9$^{wt/fl}$ and CD23Cre^{+}Myh9$^{fl/fl}$ mice (n=3 mice).

(B) Myosin IIa protein expression level of FACS-sorted follicular B cells from CD23Cre^{+}Myh9$^{wt/fl}$ and CD23Cre^{+}Myh9$^{fl/fl}$ mice.

(C) Flow cytometric analysis of myosin IIa protein expression of T1, T2 and follicular (Fo) B cells of CD23Cre^{+}Myh9$^{wt/fl}$ and CD23Cre^{+}Myh9$^{fl/fl}$ mice.

(D) Flow cytometric analysis of myosin IIa protein expression of peritoneal cavity T, B1a, B1b and B2 cells of CD23Cre^{+}Myh9$^{wt/fl}$ and CD23Cre^{+}Myh9$^{fl/fl}$ mice.

(E) Flow cytometric analysis of myosin IIa protein expression of follicular B cells of R26ERT2Cre^{+}Myh9$^{wt/wt}$, R26ERT2Cre^{+}Myh9$^{wt/fl}$ and R26ERT2Cre^{+}Myh9$^{fl/fl}$ mice 14 days after the start of tamoxifen administration.

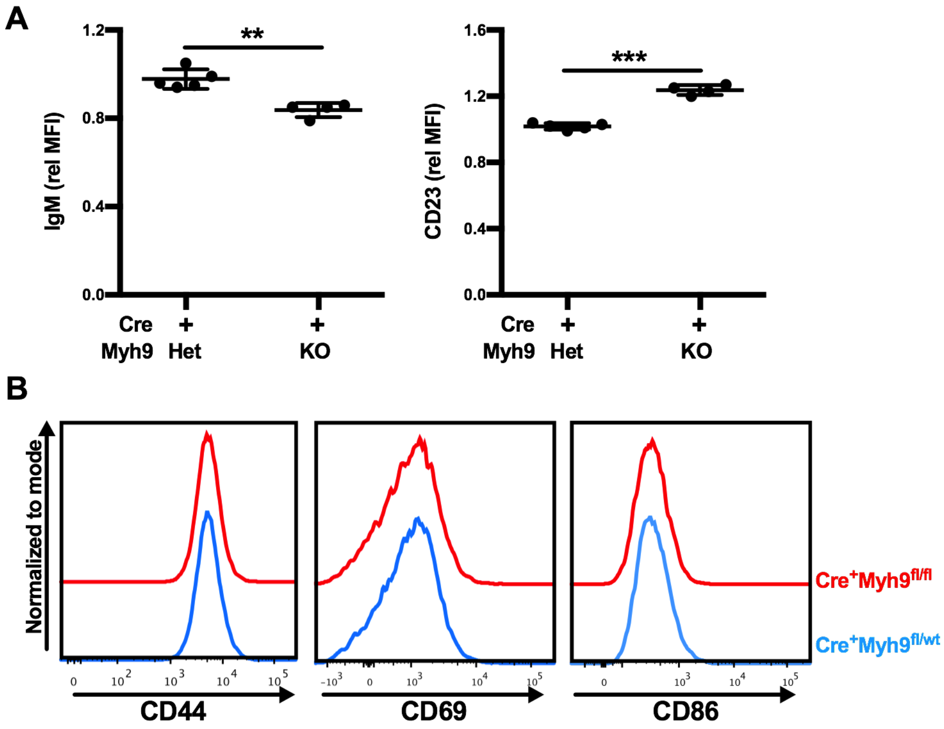

Figure S3. Follicular B cell surface marker expression of CD23Cre^{+}Myh9$^{fl/fl}$ mice, related to Figure 2.
(A) Surface expression of IgM and CD23 on follicular B cells from 20% CD23Cre^{+}Myh9$^{wt/fl}$, 80% CD45.1 and 20% CD23Cre^{+}Myh9$^{fl/fl}$, 80% CD45.1 competitive bone marrow chimeras. Normalized on expression levels of CD45.1-derived follicular B cells.
(B) Surface expression of CD44, CD69 and CD86 on follicular B cells of CD23Cre^{+}Myh9$^{wt/fl}$ and CD23Cre^{+}Myh9$^{fl/fl}$ mice.
Dots represent follicular cells from individual mice. Horizontal bars reflect mean ± SD. ** $P<0.01$, *** $P<0.001$ (Unpaired t-test).

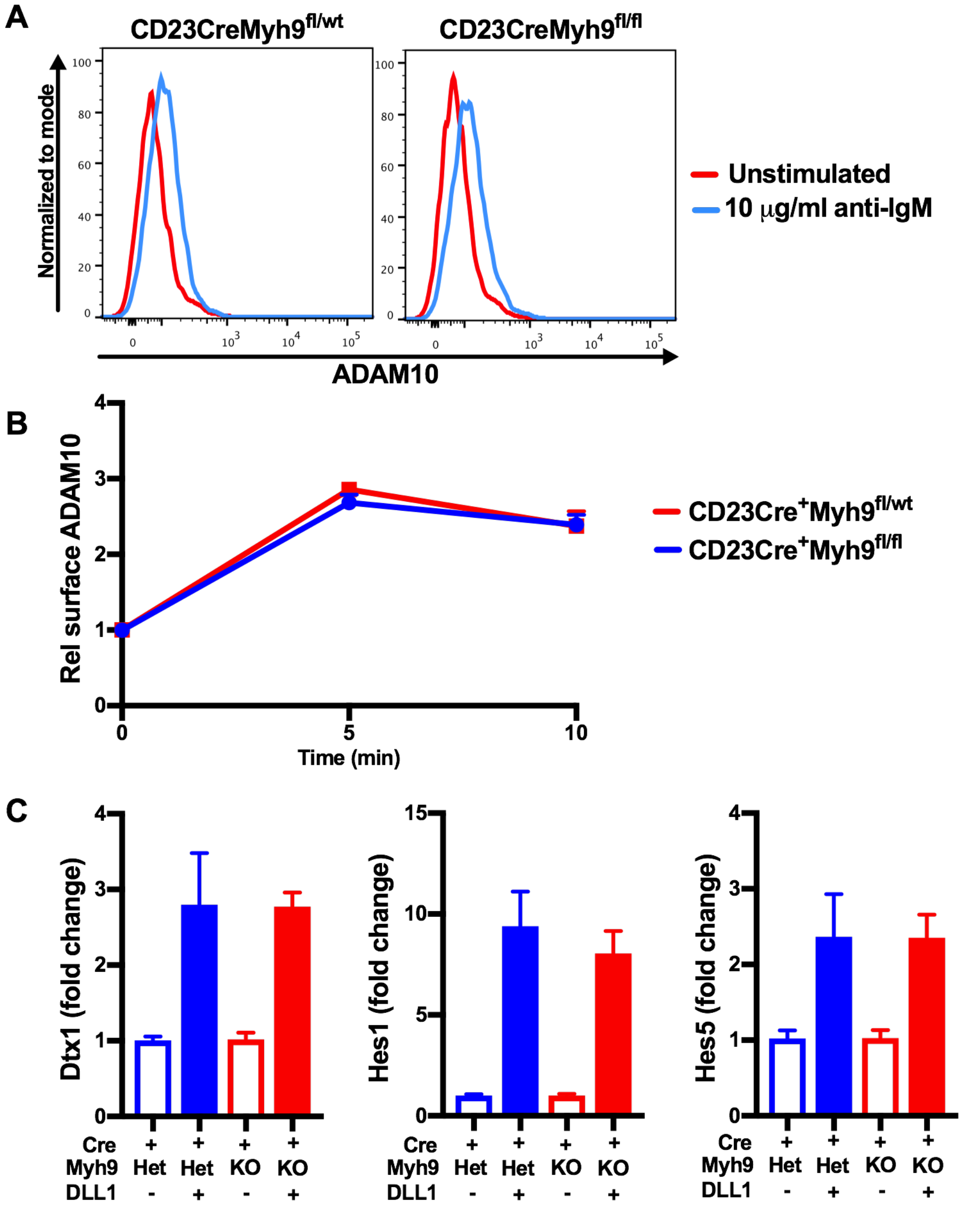

Figure S4. ADAM10 surface translocation and Notch2 signaling in CD23Cre^{+}Myh9$^{fl/fl}$ mice, related to Figure 2.

(A) Flow cytometric analysis of surface ADAM10 expression by B cells of CD23Cre^{+}Myh9$^{wt/fl}$ and CD23Cre^{+}Myh9$^{fl/fl}$ mice stimulated with soluble anti-IgM for 5 minutes.

(B) Quantification of surface ADAM10 expression by B cells of CD23Cre^{+}Myh9$^{wt/fl}$ and CD23Cre^{+}Myh9$^{fl/fl}$ mice stimulated with soluble anti-IgM.

(C) Gene expression level of Notch2 target genes *Dtx1*, *Hes1* and *Hes5* by B cells of CD23Cre^{+}Myh9$^{wt/fl}$ and CD23Cre^{+}Myh9$^{fl/fl}$ mice after 21h co-culture with OP9 that express or do not express Delta-like ligand1 (DLL1). Data normalized on expression levels on OP9 cells not expressing DLL1.

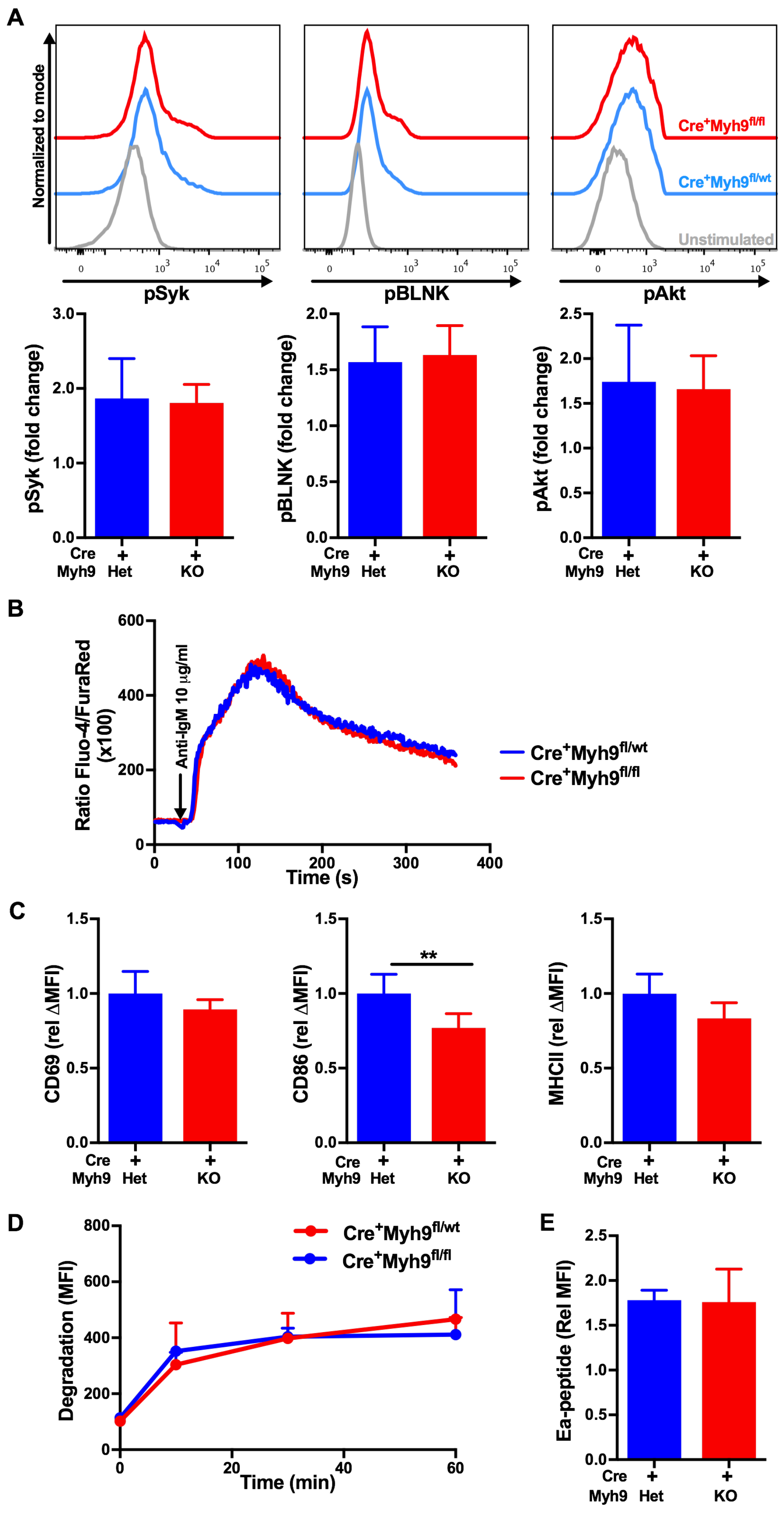
A
Normalized to mode
Cre+Myh9fl/fl
Cre+Myh9fl/wt
Unstimulated
pSyk
pBLNK
pAkt
pSyk (fold change)
pBLNK (fold change)
pAkt (fold change)
Cre + +
Myh9 Het KO
B
Ratio Fluo-4/FuraRed (x100)
Anti-IgM 10 μg/ml
Time (s)
Cre+Myh9fl/wt
Cre+Myh9fl/fl
C
CD69 (rel ΔMFI)
CD86 (rel ΔMFI)
MHCII (rel ΔMFI)
**
D
Degradation (MFI)
Time (min)
Cre+Myh9fl/wt
Cre+Myh9fl/fl
E
Ea-peptide (Rel MFI)

Figure S5. Normal levels of BCR signaling and antigen internalization of myosin IIa-deficient B cells in response to soluble antigen, related to Figure 3.

(A) Phosphorylation of Syk, BLNK and Akt in B cells of $CD23Cre^{+}Myh9^{wt/fl}$ and $CD23Cre^{+}Myh9^{fl/fl}$ mice stimulated with soluble anti-IgM for 5 (Syk, BLNK) or 15 min (Akt) and quantification (n=5 mice). Phosphorylation levels normalized on unstimulated cells.

(B) Intracellular Ca^{2+} fluxes of B cells of $CD23Cre^{+}Myh9^{wt/fl}$ and $CD23Cre^{+}Myh9^{fl/fl}$ mice after the addition of soluble anti-IgM (arrow).

(C) Upregulation of CD69, CD86 and MHCII in B cells of $CD23Cre^{+}Myh9^{wt/fl}$ and $CD23Cre^{+}Myh9^{fl/fl}$ mice after stimulation with soluble anti-IgM (n=5 mice). Data normalized on follicular B cells of $Cre^{+}Myh9^{wt/fl}$ littermates.

(D) Degradation of a DNA sensor attached to soluble anti-Igκ by B cells from $CD23Cre^{+}Myh9^{wt/fl}$ and $CD23Cre^{+}Myh9^{fl/fl}$ mice (representative for 2 experiments).

(E) Antigen presentation of $CD23Cre^{+}Myh9^{wt/fl}$ and $CD23Cre^{+}Myh9^{fl/fl}$ B cells as measured by MHCII-bound Ea peptide 5 hours after incubation with Ea peptide and anti-Igκ loaded microbeads (representative for 2 experiments). Data normalized on cells incubated with anti-Igκ loaded microbeads without Ea peptide.

Mean ± SD. ** P<0.01 (unpaired t-test).

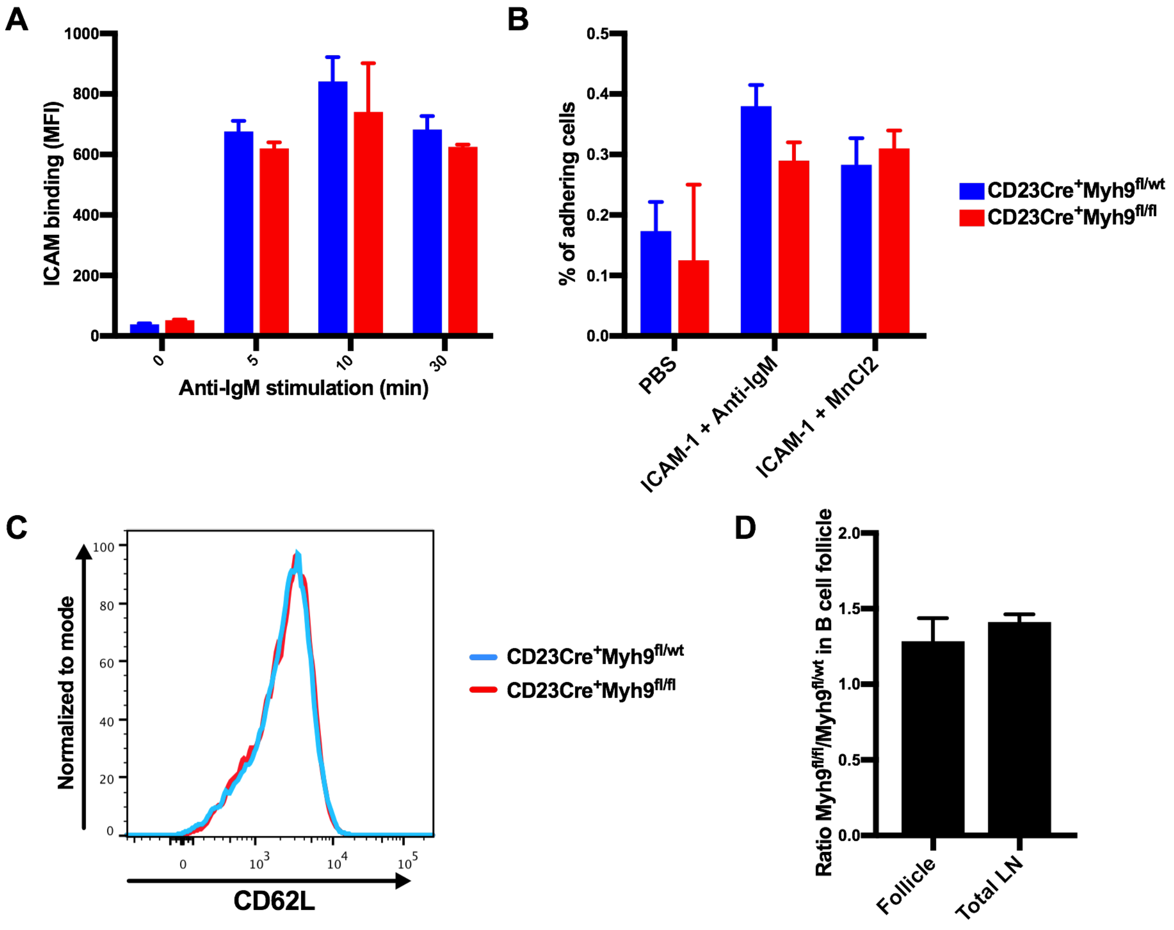

Figure S6: Initial adhesion to ICAM-1 is normal in myosin IIa-deficient B cells, related to Figure 4.
(A) Binding of soluble ICAM-1 to anti-IgM-stimulated B cells from CD23Cre^{+}Myh9$^{wt/fl}$ and CD23Cre^{+}Myh9$^{fl/fl}$ mice. Mean ± SD.
(B) Adhesion of anti-IgM and MnCL$_2$-stimulated B cells from CD23Cre^{+}Myh9$^{wt/fl}$ and CD23Cre^{+}Myh9$^{fl/fl}$ mice to immobilized ICAM-1. Mean ± SD.
(C) Surface expression of CD62L in CD23Cre^{+}Myh9$^{wt/fl}$ and CD23Cre^{+}Myh9$^{fl/fl}$ B cells.
(D) Ratio of B cells from CD23Cre^{+}Myh9$^{fl/fl}$ and CD23Cre^{+}Myh9$^{wt/fl}$ in B cell follicles 16h after i.v. injection in to C57BL/6J mice determined by immunofluorescence staining of LN cryosections and in the entire LN as assessed by flow cytometry. Ratios normalized to input ratio.

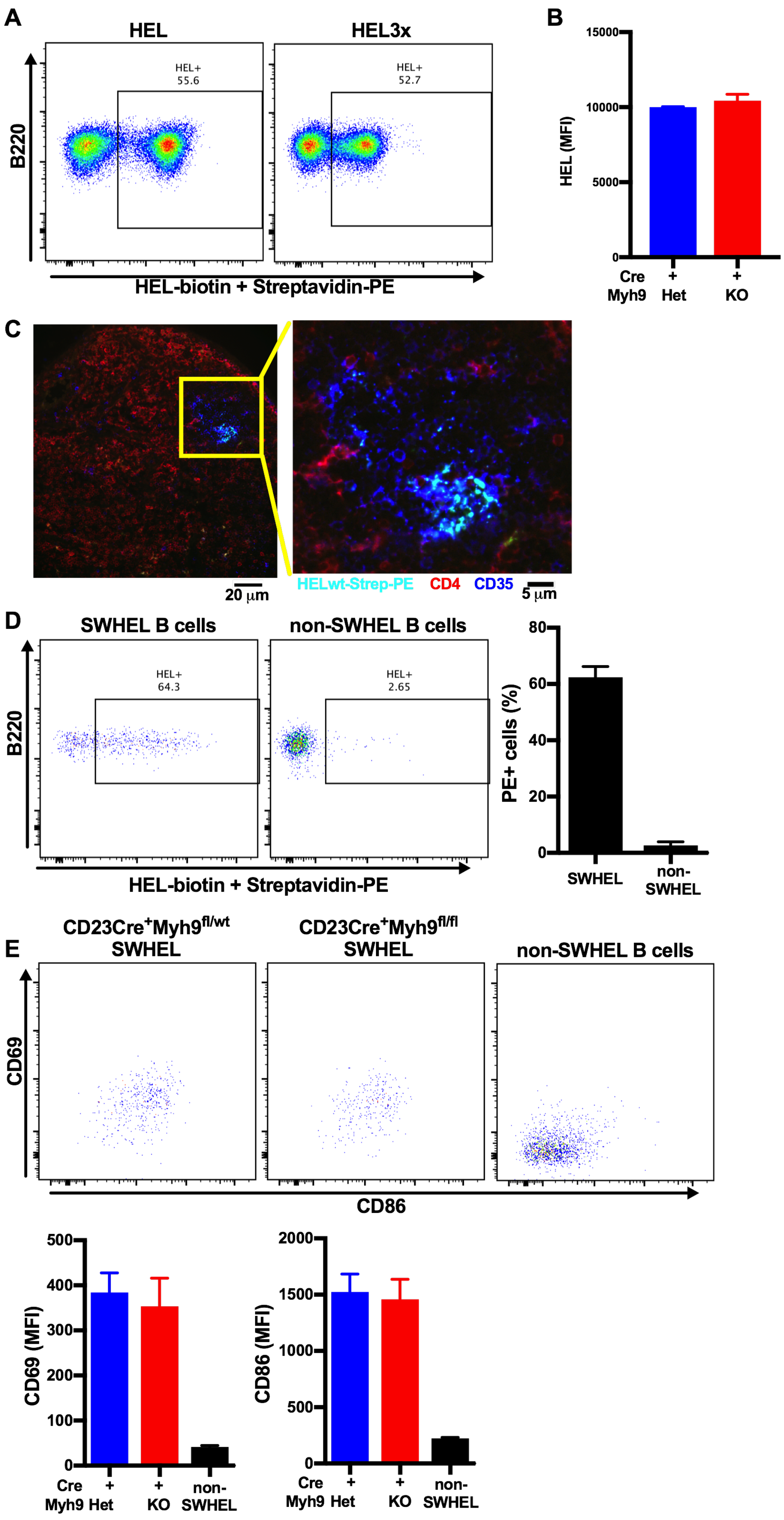

A
HEL
HEL3x
HEL+
55.6
HEL+
52.7
B220
HEL-biotin + Streptavidin-PE
B
15000
10000
5000
0
HEL (MFI)
Cre + +
Myh9 Het KO
C
20 µm
HELwt-Strep-PE CD4 CD35
5 µm
D
SWHEL B cells
non-SWHEL B cells
HEL+
64.3
HEL+
2.65
B220
HEL-biotin + Streptavidin-PE
80
60
40
20
0
PE+ cells (%)
SWHEL
non-SWHEL
E
CD23Cre+Myh9fl/wt SWHEL
CD23Cre+Myh9fl/fl SWHEL
non-SWHEL B cells
CD69
CD86
500
400
300
200
100
0
CD69 (MFI)
Cre + +
Myh9 Het KO non-SWHEL
2000
1500
1000
500
0
CD86 (MFI)
Cre + +
Myh9 Het KO non-SWHEL

Figure S7. *In vivo* antigen acquisition, related to Figure 5.
(A) Binding of HEL and HEL3x by CD23Cre^{+}Myh9$^{fl/wt}$SW$_{HEL}$ and CD23Cre^{+}Myh9$^{fl/fl}$SW$_{HEL}$ B cells *in vitro*.
(B) Quantification of HEL binding to CD23Cre^{+}Myh9$^{fl/wt}$SW$_{HEL}$ and CD23Cre^{+}Myh9$^{fl/fl}$SW$_{HEL}$ follicular B cells in vitro.
(C) HEL-Streptavidin-PE complexes in sections of lymph nodes of immunized mice.
(D) Flow cytometric analysis and quantification of HEL-Streptavidin-PE complex capture by B cells that express the SW$_{HEL}$ BCR and non-SW$_{HEL}$ expressing B cells.
(E) Flow cytometric analysis and quantification of CD69 and CD86 expression by PE-positive CD23Cre^{+}Myh9$^{fl/wt}$SW$_{HEL}$ and CD23Cre^{+}Myh9$^{fl/fl}$SW$_{HEL}$ or non-SW$_{HEL}$ expressing B cells 14 hours after transfer into HEL-Streptavidin-PE immunized CD45.1 mice.
Mean ± SD.

Table S1. Gene expression analysis of FACS-sorted follicular B cells from CD23Cre^{+}Myh9$^{wt/fl}$ and CD23Cre^{+}Myh9$^{fl/fl}$ mice (n=3), related to Figure 2

Significant: p<0.05, q<0.05.

**Downregulated**				
**Gene name**	**CD23Cre^{+}Myh9$^{wt/fl}$ (RPKM)**	**CD23Cre^{+}Myh9$^{fl/fl}$ (RPKM)**	**log2(fold_change)**	**significant**
Lcn2	12.5893	1.20524	-3.38481	yes
Ngp	13.4001	1.29461	-3.37165	yes
Igj	5.39508	0.617053	-3.12818	yes
Beta-s	51.9653	7.08096	-2.87553	yes
Hbb-b1, Hbb-b2	19.2241	3.22646	-2.57489	yes
S100a8	145.112	25.7663	-2.49361	yes
S100a9	174.91	34.5411	-2.34023	yes
Hsph1	128.76	40.3277	-1.67484	yes
Cxcr4	113.087	39.7103	-1.50985	yes
Spns2	12.775	4.99355	-1.35518	yes
Ddit4	22.5883	8.9473	-1.33605	yes
Hspa4l	13.2209	5.34704	-1.30601	yes
Myh9	50.6481	21.1252	-1.26154	yes
P4ha1	8.67193	3.66499	-1.24254	yes
Wfdc17	52.5784	22.504	-1.22429	yes
Pde3b	20.0566	9.11876	-1.13717	yes
Sik1	19.2062	8.89217	-1.11097	yes
Slc5a3	5.66703	2.7072	-1.06579	yes
Fcrl5	12.7514	6.16063	-1.0495	yes
Chordc1	81.7667	40.4015	-1.01711	yes
Zfp36l2	172.465	86.872	-0.989344	yes
Gpr171	163.275	84.7899	-0.945336	yes
Hepacam2	9.81395	5.09649	-0.945329	yes
A530032D15Rik	27.1144	14.1881	-0.934384	yes
Dusp1	15.2936	8.1218	-0.913059	yes
Stip1	54.3244	29.034	-0.903856	yes
Apoe	81.8546	44.7237	-0.872025	yes
Zfp53	12.4342	7.01072	-0.826674	yes
Mavs	25.3436	14.3302	-0.822559	yes
Ets2	16.6178	9.42273	-0.818515	yes
Plac8	137.563	82.0615	-0.745315	yes
Ahsa1	95.2149	57.763	-0.721043	yes
**Upregulated**				
**Gene name**	**CD23Cre^{+}Myh9$^{wt/fl}$ (RPKM)**	**CD23Cre^{+}Myh9$^{fl/fl}$ (RPKM)**	**log2(fold_change)**	**significant**
C2cd5	3.88898	12.1419	1.64253	yes
Crisp3	6.58979	14.0753	1.09486	yes
Cirbp	30.4702	60.7195	0.99476	yes
Il12a	18.6664	36.8476	0.98113	yes
Hrsp12	16.7785	32.1925	0.940112	yes
Slamf1	23.8185	40.2892	0.758312	yes
Slc14a1	17.166	28.51	0.731918	yes
Tnf	12.3634	22.3179	0.852123	yes

Supplemental experimental procedures

Antibodies and flow cytometry

For flow cytometric analysis, erythrocyte-lysed single cell suspensions were stained with appropriate antibodies for 20 min on ice, followed by fixation with 2% paraformaldehyde (PFA). Antibodies against the following proteins were used to identify mouse B cell subsets and stain for activation markers: B220 (RA3-6B2), CD2 (RM2-5), CD69 (H1.3F2), CD86 (GL1) and CD95/Fas (Jo2) from BD Biosciences; CD5 (53-7.3), CD19 (1D3), CD23 (B3B4), CD38 (90), CD93 (AA4.1), IgM (II/41) and MHCII (M5/114.15.2) from eBioscience; CD21 (7E9), CD45.1 (A20) and CD45.2 (104) from BioLegend; ADAM10 (139712) from Novus Biologicals. Live/Dead fixable near-IR Dead Cell stain (ThermoFisher) was used to exclude dead cells. Cells were analyzed on BD FACSCanto II or Fortessa analyzers and sorted on Beckman Coulter MoFlo XDP cell sorters. Data was analyzed using FlowJo 10.0.7 (TreeStar).

Signaling, activation and proliferation

For signaling, activation, imaging and cell culture experiments, cell suspensions were enriched for B cells by negative selection using anti-CD43 beads (Miltenyi) on an autoMACS cell separator (Miltenyi).

To analyze phosphorylation of BCR signaling pathway components, B cells were stimulated with 10 µg/ml anti-IgM (Jackson ImmunoResearch), fixed with 2% PFA, permeabilized with the Foxp3 fixation/permeabilization kit (eBioscience) and stained with phosphorylation-specific antibodies for Syk (C87C1), Erk (D13.14.4E), Akt (193H12) and BLNK (J117-1278), all from BD Biosciences. To investigate intracellular Ca^{2+} fluxes, B cells were labeled with 1 µM Fluo-4 and 1 µM FuraRed (both from ThermoFisher), B220 antibody (RA3-6B2) and live/dead fixable near-IR Dead Cell stain (ThermoFisher). Baseline fluorescence of the dyes was analyzed for 25 seconds by flow cytometry. Then, 10 µg/ml of anti-IgM was added (Jackson ImmunoResearch) and fluorescence of the dyes was acquired for a further 300 seconds. To analyze activation, B cells were stimulated overnight with 10 µg/ml anti-IgM, fixed in 2% PFA and stained for surface activation markers.

To study proliferation, B cells were labeled with 1 µM 5-chloromethylfluorescein diacetate (CMFDA, ThermoFisher) and cultured for 48 hours in DMEM supplemented with 10% fetal calf serum (BioSera), 100 µM non-essential amino acids, 20 mM HEPES, 2 mM glutamine, 50 µM beta-mercaptoethanol, 100 U/ml penicillin and 100 µg/ml streptomycin. When indicated cells were stimulated with 10 µg/ml anti-IgM (Jackson ImmunoResearch), 10 µg/ml LPS (Sigma), 100 µM CpG (Sigma), 1 µg/ml CD40L (R&D Systems) or 100 ng/ml IL-4 (Peprotech). Dilution of CMFDA was analyzed by flow cytometry. To analyze cell cycle progression, cells were stimulated with 100 µM CpG for 48 hours, fixed in PFA, stained for surface markers and permeabilized with the FoxP3 permeabilization kit. Then, DNA was stained with propidium iodide (Sigma) and analyzed by flow cytometry.

In vitro antigen internalization and presentation

Internalization of soluble antigen was analyzed using DNA-based degradation sensors as described (Nowosad et al., 2016). In short, 100 nM of complementary DNA oligomers, one strain labeled with Atto647N and one with quencher Iowa Black RQ, were annealed and attached to anti-Igκ (Southern Biotech) and incubated with purified B cells on ice. After washing, cells were incubated at 37ºC and unquenching of Atto647N fluorescence was analyzed by flow cytometry.

To detect antigen presentation, B cells were incubated with Ea-peptide (Biotin-GSGFAKFASFEAQGALANIAVDKA-COOH) and anti-Igκ-loaded microbeads (Bangs laboratories) for 5 hours at 37ºC. Cells were fixed with 2% PFA and stained with anti-MHCII-Ea antibody (M5/114.15.2, eBioscience) followed by a fluorescently labeled anti-rat IgG2b antibody (ThermoFisher).

Large scale imaging

PMS were prepared by shearing off HEK293 cells seeded in poly-L-lysine coated Lab-Tek imaging chambers (ThermoFisher) by sonication, as described (Natkanski et al., 2013). Subsequently, remaining membrane patches were blocked with 1% bovine serum albumin (BSA) and decorated with an in-house biotinylated and Cy3-labeled goat anti-mouse Igκ $F(ab')_2$ antibody using Annexin V-biotin (BioVision) and streptavidin (Sigma). B cells were labeled with either Cell Trace Violet (CTV) or Cell Trace Far Red (CTFR) (both from ThermoFisher) and mixed with unlabeled control cells or vice versa. Cells were resuspended in warm 0.1% BSA in Hanks-buffered salt solution (HBSS) and incubated in pre-warmed imaging chambers for indicated times. Incubation was stopped by adding PFA. After blocking with normal mouse serum (Jackson ImmunoResearch) cells were stained for B220, followed by permeabilization with the Foxp3 fixation/permeabilization kit and staining for phosphorylated BCR signaling pathway components.

Automated image acquisition was performed by a motorized IX81 microscope with a 100x objective (Olympus) and motorized stage with integrated piezo Z-drive, both controlled by Metamorph software (Molecular Devices). Illumination was supplied by 405 nm (Changchun New Industries), 488 nm, 514 nm (both from LS300, Dynamic

Lasers) and 640 nm lasers (Blue Sky Research). Up to 900 fields of view were acquired per imaging chamber. Images were processed and analyzed using Matlab (Mathworks)-based algorithms developed as described (Nowosad et al., 2016). In short, interactive modules were programmed for (1) determination and correction of background, flatfield and spectral bleedtrough; (2) processing and analysis of three-dimensional multichannel images and identification of cells; (3) display and gating of data. Algorithms are available on request.

Migration and adhesion

To analyze binding of soluble ICAM-1, B cells were stimulated with 10 µg/ml anti-IgM or 10 mM $MnCl_2$ in the presence of soluble ICAM-1-Fc chimera protein (R&D systems) complexed to fluorescently-labeled anti-IgG. Cells were fixed with PFA after 5, 10 and 30 minutes and binding of ICAM-1 was analyzed by flow cytometry. To determine adhesion to immobilized ICAM-1, 96-well plates were coated with 5 µg/ml ICAM-1-Fc and blocked with 2% BSA in PBS. Purified B cells were labeled with CTV, resuspended in 0.1% BSA in HBSS and allowed to adhere in the presence of 10 µg/ml anti-IgM or 0.5 mM $MnCl_2$ for 30 minutes at 37ºC. Adherence of cells was analyzed by measuring CTV fluorescence.

To study migration by timelapse imaging, Lab-Tek imaging chambers (ThermoFisher) were coated with ICAM-1-Fc chimera protein and blocked with 2% BSA in PBS. B cells were activated with 1 µg/ml CXCL13 (R&D systems) for 15 minutes and added to warmed ICAM-1-coated chambers containing 1 µg/ml CXCL13 in 0.1% BSA in HBSS. Cells were allowed to adhere for 10 minutes and then brightfield images were acquired every 5 seconds for 40 minutes. TrackMate was used to identify and track cells (Tinevez et al., 2017).

Transwell migration assays were performed using 96 well Transwell inserts with 5 µm polycarbonate membranes (Sigma-Aldrich). Top compartments were coated with 0.5 µg/ml ICAM-1 or 1 µg/ml anti-Igκ $F(ab')_2$ in PBS, followed by blocking with 2% BSA in PBS. 80.000 cells were added to top compartments and allowed to migrate towards 1 µg/ml CXCL13, 0.5 µg/ml CXCL12 or 1 µg/ml CCL21 (all from R&D systems) in 0.1% FBS in RPMI in the bottom compartment for 3 hours. Migrated B cells were quantified using flow cytometry.

Gene expression analysis

RNA was isolated from sorted follicular B cells using TriZol (ThermoFisher) and further purified using a RNEasy mini kit (Qiagen). After RNA amplification (Ovation RNA-Seq system V2, NuGen) stranded polyA-enriched libraries were prepared using a TruSeq Nano DNA kit (Illumina), followed by sequencing on a HiSeq 2500 (Illumina). Reads were aligned to C57BL/6J mouse reference genome mm10, GRCm38 and quantified using Tophat and Cufflinks, as described elsewhere (Roberts et al., 2012).

To analyze expression of Notch target genes, RNA was extracted and reverse transcribed using the RNEasy mini and Quantitect Reverse Transcription kits, respectively. Gene expression was analyzed using Taqman technology, probes Mm00492297_m1 (*Deltex1*), Mm01342805_m1 (*Hes1*) and Mm00439311_g1 (*Hes5)* on a Quantstudio 3 Real-Time PCR machine (Applied Biosystems). Data was normalized on expression of *HPRT* (probe Mm00446968).

Immunizations and ELISA

Mice were immunized by i.p. injection of 50 µg NP-Ficoll or NP-CGG (BioSearch) suspended in alum (ThermoFisher). Serum blood samples were withdrawn before and 7 and 10 days after immunization. To detect NP-specific antibodies by ELISA, nunc polysorb plates were coated with 5 µg/ml NP_{13}-BSA (BioSearch), blocked with 3% BSA in PBS and incubated with serial dilutions of serum, followed by incubation with HRP-labeled anti-IgM, anti-IgG1 or anti-IgG3 antibodies (Southern Biotech). Bound HRP was analyzed using 3,3',5,5'-tetra-methylbenzidine (TMB). Steady-state serum immunoglobulin levels were determined using the SBA Clonotyping System-C57BL/6-HRP kit (Southern Biotech).

References

Natkanski, E., Lee, W.-Y., Mistry, B., Casal, A., Molloy, J.E., Tolar, P., 2013. B Cells Use Mechanical Energy to Discriminate Antigen Affinities. Science 340, 1587–1590.

Nowosad, C.R., Spillane, K.M., Tolar, P., 2016. Germinal center B cells recognize antigen through a specialized immune synapse architecture. Nat Immunol 17, 870–877.

Roberts, A., Goff, L., Pertea, G., Kim, D., Kelley, D.R., Pimentel, H., Salzberg, S.L., Rinn, J.L., Pachter, L., Trapnell, C., 2012. Differential gene and transcript expression analysis of RNA-seq experiments with TopHat and Cufflinks. Nature Protocols 7, 562–578.

Tinevez, J.-Y., Perry, N., Schindelin, J., Hoopes, G.M., Reynolds, G.D., Laplantine, E., Bednarek, S.Y., Shorte, S.L., Eliceiri, K.W., 2017. TrackMate: An open and extensible platform for single-particle tracking. Methods 115, 80–90.
