# Supplementary material for: Myosin IIa Promotes Antibody Responses by Regulating B Cell Activation, Acquisition of Antigen, and Proliferation
Source: Cell Rep. 2018 May 22;23(8):2342–53. doi: 10.1016/j.celrep.2018.04.087 (PMC5986709; doi:10.1016/j.celrep.2018.04.087)
Supplement: Document S2. Article plus Supplemental Information [file mmc3.pdf]

Cell Reports

Article

Myosin IIa Promotes Antibody Responses by Regulating B Cell Activation, Acquisition of Antigen, and Proliferation

Graphical Abstract

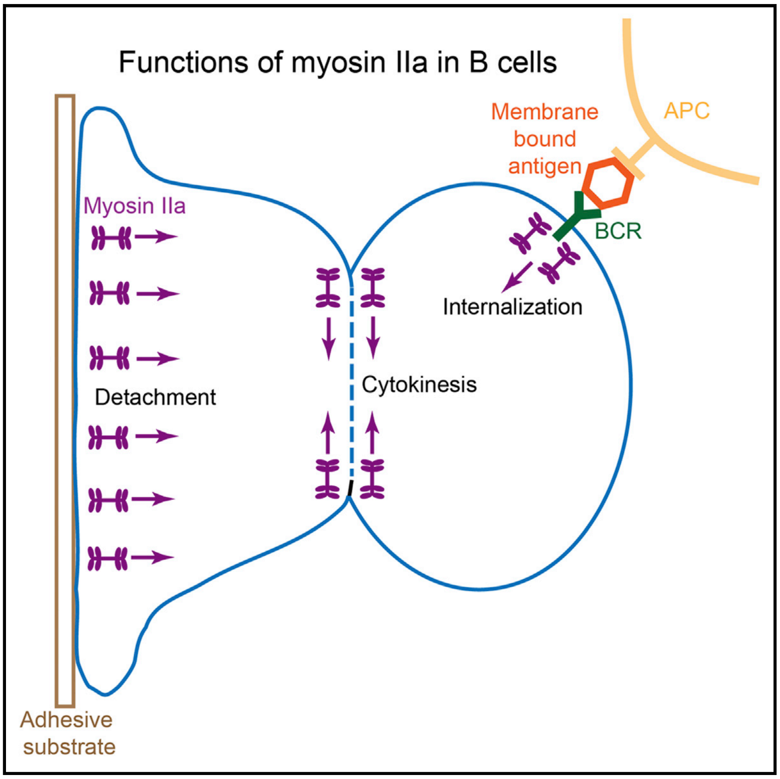

Highlights

- Myosin IIa is important for B cell antigen acquisition from antigen-presenting cells
- Myosin IIa is a negative regulator of B cell activation
- Myosin IIa is essential for B cell cytokinesis
- Myosin IIa is required for efficient B cell responses

Authors

Robbert Hoogeboom, Elizabeth M. Natkanski, Carla R. Nowosad, Dessislava Malinova, Rajesh P. Menon, Antonio Casal, Pavel Tolar

Correspondence

pavel.tolar@crick.ac.uk

In Brief

B cell antigen acquisition, processing, and presentation may depend on contractile activity of the actomyosin cytoskeleton. Here, Hoogeboom et al. show that non-muscle myosin IIa positively regulates B cell antigen acquisition from antigen-presenting cells *in vivo*. In addition, myosin IIa negatively regulates B cell activation and is required for B cell cytokinesis.

Data and Software Availability

GSE113114

Hoogeboom et al., 2018, Cell Reports *23*, 2342–2353
May 22, 2018 © 2018 The Author(s).
https://doi.org/10.1016/j.celrep.2018.04.087

OPEN ACCESS
CellPress

Cell Reports
Article

Myosin IIa Promotes Antibody Responses by Regulating B Cell Activation, Acquisition of Antigen, and Proliferation

Robbert Hoogeboom,[1,2] Elizabeth M. Natkanski,[1] Carla R. Nowosad,[1] Dessislava Malinova,[1,3] Rajesh P. Menon,[1] Antonio Casal,[1] and Pavel Tolar[1,3,4,*]
[1]Immune Receptor Activation Laboratory, The Francis Crick Institute, London NW1 1AT, UK
[2]Department of Haemato-Oncology, Faculty of Life Sciences and Medicine, King's College London, London SE5 9NU, UK
[3]Division of Immunology & Inflammation, Department of Medicine, Imperial College London, London SW7 2A2, UK
[4]Lead Contact
*Correspondence: pavel.tolar@crick.ac.uk
https://doi.org/10.1016/j.celrep.2018.04.087

SUMMARY

B cell responses are regulated by antigen acquisition, processing, and presentation to helper T cells. These functions are thought to depend on contractile activity of non-muscle myosin IIa. Here, we show that B cell-specific deletion of the myosin IIa heavy chain reduced the numbers of bone marrow B cell precursors and splenic marginal zone, peritoneal B1b, and germinal center B cells. In addition, myosin IIa-deficient follicular B cells acquired an activated phenotype and were less efficient in chemokinesis and extraction of membrane-presented antigens. Moreover, myosin IIa was indispensable for cytokinesis. Consequently, mice with myosin IIa-deficient B cells harbored reduced serum immunoglobulin levels and did not mount robust antibody responses when immunized. Altogether, these data indicate that myosin IIa is a negative regulator of B cell activation but a positive regulator of antigen acquisition from antigen-presenting cells and that myosin IIa is essential for B cell development, proliferation, and antibody responses.

INTRODUCTION

B cell activation is initiated when B cells bind antigen via their cell-surface B cell antigen receptors (BCRs). This induces signaling and internalization of BCR-antigen complexes. Subsequently, antigen is trafficked along the endosomal pathway, processed into peptides, and loaded on major histocompatibility complex class II (MHC class II) molecules for presentation to T cells. Cognate interaction with T cells results in full activation and proliferation of the B cell and differentiation into high-affinity antibody-secreting cells. Some B cells, e.g., marginal zone (MZ) B cells, mostly encounter small soluble antigens. However, many B cells engage antigen bound to antigen-presenting cells (APCs), such as subcapsular macrophages and follicular dendritic cells (FDCs), which display unprocessed antigen bound to complement or Fc receptors on their cell surfaces (Carrasco and Batista, 2007; Gonzalez et al., 2010; Junt et al., 2007; Phan et al., 2007; Qi et al., 2006). In contrast to acquisition of free-floating soluble antigen, capture of membrane-bound antigens from APCs requires B cells to apply force to overcome the membrane tether. It has been hypothesized that non-muscle myosin IIa generates these forces.

Myosin IIa is a motor protein from the class II family of myosin proteins, which have been implicated in generation of cortical tension (Murrell et al., 2015), separation of the mitotic spindle (Rosenblatt et al., 2004), formation of the cleavage furrow during cytokinesis (Straight et al., 2003), and cellular locomotion (Kolega, 1998). Myosin IIa is the only class II myosin expressed in lymphocytes. In T cells, it regulates maturation of immune synapses (Kumari et al., 2012), de-adhesion from intercellular adhesion molecule-1 (ICAM-1) (Morin et al., 2008), and interstitial migration and lymph node retention (Jacobelli et al., 2010). *In vitro* studies using primary B cells or B cell lines treated with blebbistatin, an inhibitor of class II myosin proteins, revealed a role for myosin IIa in B cell antigen extraction from membrane substrates (Natkanski et al., 2013) and antigen presentation to T cells (Vascotto et al., 2007). However, the role of myosin IIa in B cell functions *in vivo* has not been investigated.

Here, using mice in which myosin IIa was conditionally or inducibly deleted from B cells, we show that myosin IIa is required for B cell development at the pro-B cell stage. Moreover, when we deleted myosin IIa in more mature B cells, development and maintenance of splenic MZ, peritoneal B1b, and steady-state germinal center (GC) B cells was disturbed. Myosin IIa-deficient follicular B cells developed normally; however, these cells acquired an activated phenotype. Culturing myosin IIa-deficient B cells in the presence of various activating stimuli revealed a defect in cytokinesis. In addition, myosin IIa-deficient B cells showed impaired migration and were less efficient in internalizing membrane-tethered antigen, whereas internalization of soluble antigen was unperturbed. We also observed reduced acquisition of antigen from FDCs *in vivo*. Collectively, these defects resulted in reduced steady-state serum antibody levels and diminished antibody responses *in vivo*.

 © 2018 The Author(s).
This is an open access article under the CC BY license (http://creativecommons.org/licenses/by/4.0/).

OPEN
ACCESS
CellPress

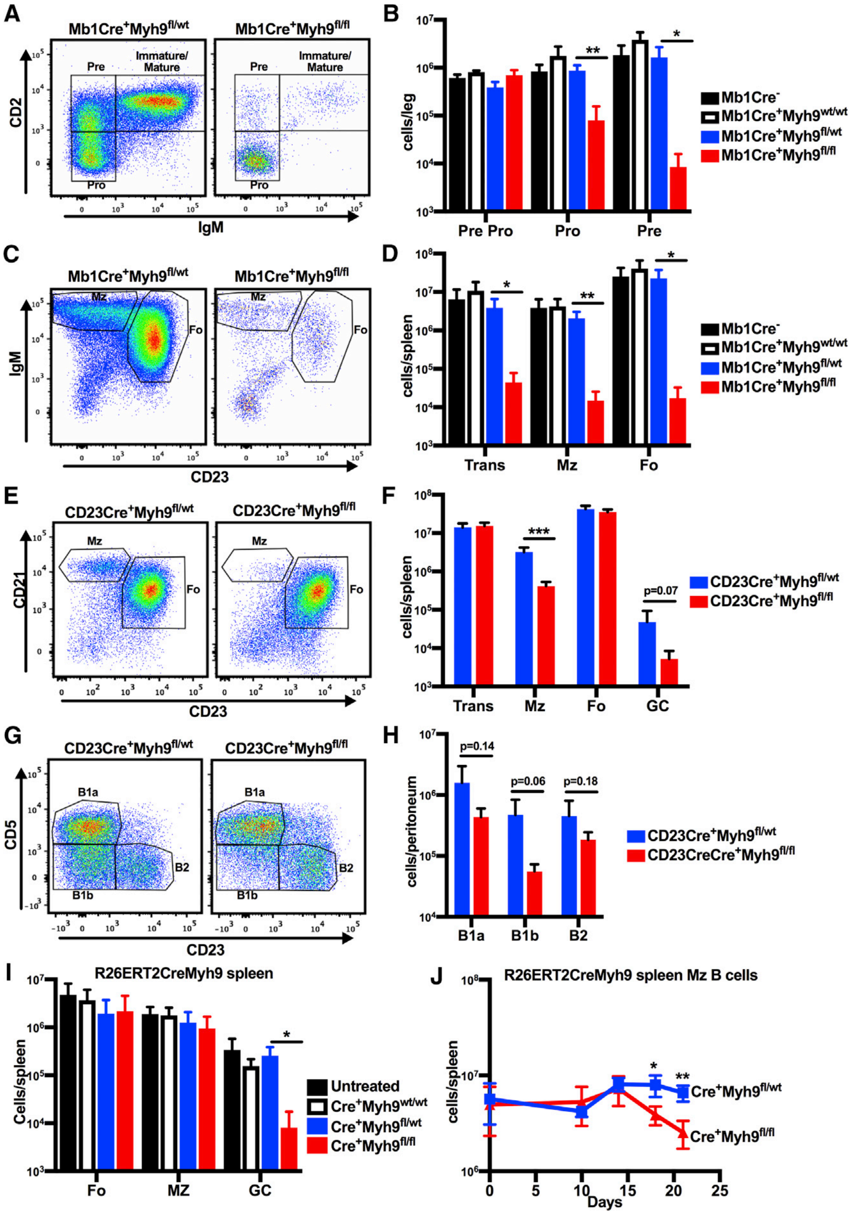

Figure 1. Disturbed B Cell Development and Maintenance after B Cell-Specific Deletion of Myosin IIa

(A) Flow cytometric analysis of B220$^+$CD19$^+$ cells in the bone marrow (BM) of Mb1Cre$^+$Myh9$^{wt/fl}$ and Mb1Cre$^+$Myh9$^{fl/fl}$ mice.

(B) Quantification of precursor B cell subsets in the BM of Mb1Cre$^+$Myh9$^{wt/wt}$ (n = 3), Mb1Cre$^+$Myh9$^{wt/fl}$ (n = 4), Mb1Cre$^+$Myh9$^{fl/fl}$ (n = 4), and Cre-negative littermates (n = 4).

(C) Flow cytometric analysis of B220$^+$AA4.1$^-$ cells in spleens of Mb1Cre$^+$Myh9$^{wt/fl}$ and Mb1Cre$^+$Myh9$^{fl/fl}$ mice.

(D) Quantification of B cell subsets in spleens of Mb1Cre$^+$Myh9$^{wt/wt}$ (n = 3), Mb1Cre$^+$Myh9$^{wt/fl}$ (n = 4), Mb1Cre$^+$Myh9$^{fl/fl}$ (n = 4), and Cre-negative littermates (n = 4).

(E) Flow cytometric analysis of B220$^+$AA4.1$^-$ cells in spleens of CD23Cre$^+$Myh9$^{wt/fl}$ and CD23Cre$^+$Myh9$^{fl/fl}$ mice.

(F) Quantification of B cell subsets in spleens of CD23Cre$^+$Myh9$^{wt/fl}$ (n = 6) and CD23Cre$^+$Myh9$^{fl/fl}$ (n = 4) mice.

(G) Flow cytometric analysis of B220$^+$ cells in the peritoneal cavity of CD23Cre$^+$Myh9$^{wt/fl}$ and CD23Cre$^+$Myh9$^{fl/fl}$ mice.

(H) Quantification of B cell subsets in the peritoneal cavity of CD23Cre$^+$Myh9$^{wt/fl}$ (n = 6) and CD23Cre$^+$Myh9$^{fl/fl}$ (n = 4) mice.

(I) Quantification of B cell subsets 14 days after the start of tamoxifen treatment in spleens of R26ERT2Cre$^+$Myh9$^{wt/wt}$, R26ERT2Cre$^+$Myh9$^{wt/fl}$, R26ERT2Cre$^+$Myh9$^{fl/fl}$, and Cre-negative mixed BM chimeras (n = 5).

(J) Quantification of MZ B cell numbers in spleens of R26ERT2Cre$^+$Myh9$^{wt/fl}$ and R26ERT2Cre$^+$Myh9$^{fl/fl}$ mice over time since the start of tamoxifen treatment.

Plotted are means ± SD per mouse. *$p < 0.05$, **$p < 0.01$, ***$p < 0.001$ (unpaired t test). Trans, transitional B cells; MZ, marginal zone B cells; Fo, follicular B cells; GC, B220$^+$Fas$^+$CD38$^-$ germinal center B cells. See also Figures S1 and S2.

RESULTS

Myosin IIa Is Required for Bone Marrow B Cell Development

Germline knockout of *Myh9*, encoding the myosin IIa heavy chain, leads to embryonic death (Conti et al., 2004). To study the role of myosin IIa in B cells, we crossed Myh9$^{fl/fl}$ mice, in which exon 3 of *Myh9* is flanked by LoxP sites (Jacobelli et al., 2010), with Cd79aCre (Mb1Cre) and Fcer2Cre (CD23Cre) mice, resulting in mice in which *Myh9* is conditionally deleted from early bone marrow (BM) B cell precursors and more mature splenic transitional B cells, respectively (Hobeika et al., 2006; Kwon et al., 2008). Flow cytometric analysis of the BM and peripheral lymphoid organs of Mb1Cre$^+$Myh9$^{fl/fl}$ mice revealed severely reduced numbers of pro-B cells and in all subsequent stages of B cell development compared to Mb1Cre$^+$Myh9$^{wt/fl}$, Mb1Cre$^+$Myh9$^{wt/wt}$, or Cre-negative littermates (Figures 1A–1D), demonstrating that B cell development is blocked immediately after first expression of *Cd79a*. No differences were found in B cell development or mature B cell numbers between haploinsufficient and myosin IIa-wild-type mice, suggesting that a partial reduction of myosin IIa levels does not impair B cell development or maintenance of mature B cells. We conclude that myosin IIa is essential for early steps of B cell development.

Myosin IIa Regulates Development and Maintenance of Splenic MZ and Peritoneal B1b B Cells

To investigate the role of myosin IIa in mature B cells, we analyzed CD23Cre$^+$Myh9$^{fl/fl}$ mice and found that total mature B cell numbers in the spleen, BM, and lymph nodes (LNs) were

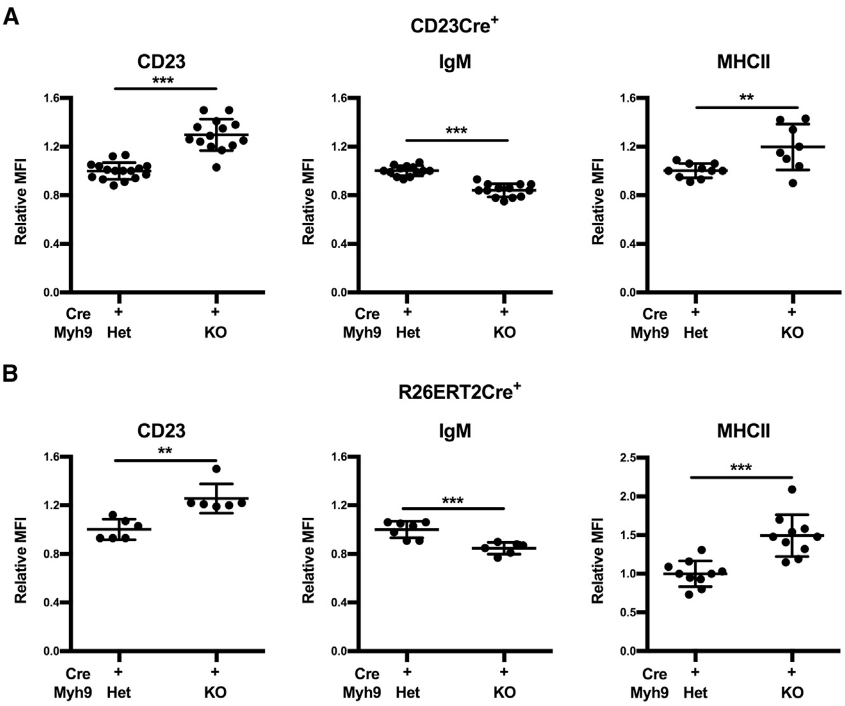

Figure 2. Myosin IIa-Deficient Follicular B Cells Express Altered Levels of Surface Activation Markers

(A) Surface expression of CD23, IgM, and MHC class II on follicular B cells of CD23Cre^{+}Myh9$^{wt/fl}$ and CD23Cre^{+}Myh9$^{fl/fl}$ mice.

(B) Relative expression of surface CD23, IgM, and MHC class II on follicular B cells of R26ERT2Cre^{+-}Myh9$^{wt/fl}$ and R26ERT2Cre^{+}Myh9$^{fl/fl}$ mice 18 days after the start of tamoxifen treatment.

Each dot represents one mouse. Horizontal bars reflect mean ± SD. Data are normalized on expression levels of Cre^{+}Myh9$^{wt/fl}$ littermate follicular B cells. $^{**}p < 0.01$, $^{***}p < 0.001$ (unpaired t test). See also Figure S3.

normal (Figures 1E, 1F, S1A, and S1B). However, splenic MZ and steady-state GC B cell numbers were reduced (Figures 1E and 1F). In the peritoneal cavity, the numbers of B1b B cells were also reduced, whereas B1a and B2 numbers were similar as in control mice (Figures 1G and 1H). Deletion of myosin IIa in follicular B cells was confirmed by analyzing mRNA expression of *Myh9* exon 3 and myosin IIa protein expression by western blot (Figures S2A and S2B). In addition, reduced myosin IIa protein levels were detected by flow cytometry in splenic CD23-expressing T2 cell and follicular B cell subsets of CD23Cre^{+}Myh9$^{fl/fl}$ mice, whereas CD23-negative T1 cells expressed normal levels (Figure S2C). In the peritoneal cavity, we observed reduced myosin IIa protein levels in B1b and B2 cells. However, B1a B cells retained myosin IIa expression (Figure S2D), most likely because these cells derive from fetal liver cells that do not express CD23.

The loss of MZ B cells was B cell intrinsic, because it was recapitulated when BM of CD23Cre^{+}Myh9$^{fl/fl}$ was mixed with 4 volumes of CD45.1 BM and transferred into sub-lethally irradiated *Rag1*-knockout (KO) mice (Figure S1C). Competition with wild-type B cells also reduced the numbers of myosin IIa-deficient follicular B cells, although not as severely as the number of MZ B cells.

Splenic MZ and peritoneal B1 B cells may share developmental and maintenance cues (Niiro and Clark, 2002). To investigate whether myosin IIa plays a role in the development or maintenance of MZ B cells, we mixed BM cells from R26ERT2Cre^{+}Myh9$^{fl/fl}$ and muMT mice and transferred them into sub-lethally irradiated *Rag1*-KO mice. In the resulting mice, myosin IIa can be acutely deleted specifically in B cells by administration of tamoxifen. Reduced levels of myosin IIa protein were detected in peripheral blood B cells by day 8 after the start of tamoxifen treatment, with maximally reduced levels from day 14 onward (Figure S2E). On day 14 after starting tamoxifen administration, MZ B cell numbers were not significantly different among R26ERT2Cre^{+}Myh9$^{wt/wt}$, R26ERT2Cre^{+}Myh9$^{wt/fl}$, and R26ERT2Cre^{+}Myh9$^{fl/fl}$ chimeras (Figure 1I). However, R26ERT2Cre^{+}Myh9$^{fl/fl}$ MZ B cell numbers started to decline 18 days after the start of tamoxifen treatment (Figure 1J). In contrast, steady-state R26ERT2Cre^{+}Myh9$^{fl/fl}$ GC B cell numbers were already reduced at day 14 (Figure 1I), indicating that myosin IIa is required for acute maintenance of GC B cells, but not MZ B cells. However, myosin IIa is important for MZ B cell development and long-term maintenance.

Development and maintenance of MZ B cells requires correct localization to the MZ (Lu and Cyster, 2002). To investigate localization of myosin IIa-deficient MZ B cells, we intravenously (i.v.) injected R26ERT2Cre^{+}Myh9$^{fl/fl}$ BM chimeras with an anti-Cd19-phycoerythrin (PE) antibody to label cells exposed to blood 14 days after the start of tamoxifen treatment. At this time point, myosin IIa is deleted, but MZ B cell numbers have not yet declined. Five minutes after injection of anti-Cd19-PE antibody, mice were culled and binding of antibody was analyzed by flow cytometry. Labeling of MZ B cells was slightly increased in R26ERT2Cre^{+}Myh9$^{fl/fl}$ chimeras (Figure S1D), indicating that myosin IIa-deficient MZ B cells were localized to the MZ or red pulp before their disappearance.

Myosin IIa-Deficient Follicular B Cells Display Elevated Surface Activation Markers

Although follicular B cells in CD23Cre^{+}Myh9$^{fl/fl}$ mice developed in normal numbers, they expressed higher levels of surface Fcer2 (CD23) and MHC class II and reduced levels of surface immunoglobulin M (IgM) (Figure 2A), indicating an activated phenotype. A similar follicular B cell surface marker phenotype was induced by acute depletion of myosin IIa in R26ERT2Cre^{+}Myh9$^{fl/fl}$ mixed BM chimeras (Figure 2B), suggesting that myosin IIa is continuously required to maintain a resting surface marker phenotype of follicular B cells. The surface marker phenotype

was B cell intrinsic, because it was also observed in myosin IIa-deficient follicular B cells of 20% CD23Cre^{+}Myh9$^{fl/fl}$, 80% CD45.1 mixed BM chimeras (Figure S3A). However, we could not detect significant changes in expression of other activation markers, such as Cd44, Cd69, and Cd86 (Figure S3B). No differences were found in surface marker expression of B cells with wild-type and haplosufficient myosin IIa levels (data not shown), indicating that a partial reduction of myosin IIa levels has no effect on the surface marker phenotype.

To find clues of the signaling pathways that drive these phenotypic changes, we sorted follicular B cells of CD23Cre^{+}Myh9$^{wt/fl}$ and CD23Cre^{+}Myh9$^{fl/fl}$ mice by flow cytometry and analyzed gene expression by RNA sequencing. In myosin IIa-deficient B cells, 8 genes were significantly upregulated and 32 genes were significantly downregulated compared to haploinsufficient B cells (Table S1). The downregulated genes included the putative p53 target genes *Dusp1*, *Ets2*, *S100a9*, and *Zpf36l2*, in line with a report that myosin IIa post-transcriptionally stabilizes p53 (Schramek et al., 2014). However, the RNA sequencing (RNA-seq) data did not reveal clues as to what signaling pathways may be dysregulated in myosin IIa-deficient B cells.

MZ B cell development requires Adam10-mediated Notch2 cleavage and signaling (Gibb et al., 2010; Hammad et al., 2017), an event that might depend on myosin IIa-mediated forces or tension. To interrogate the role of myosin IIa in Adam10 translocation to the plasma membrane, we stimulated myosin IIa-deficient B cells with soluble anti-IgM and analyzed Adam10 surface expression by flow cytometry. A similar increase in Adam10 surface expression was detected in myosin IIa-deficient and myosin IIa-proficient B cells (Figures S4A and S4B). In agreement, myosin IIa-deficient B cells normally upregulated the Notch2 target genes *Deltex1*, *Hes1*, and *Hes5* when cultured on OP9 cells expressing the Notch ligand Dll1 (Figure S4C). We conclude that myosin IIa is not involved in Notch2 signaling.

BCR Signaling and Internalization of Soluble Antigen Are Normal in Myosin IIa-Deficient B Cells

A lack of MZ B cell development, upregulation of CD23 and MHC class II, and decreased surface IgM expression have been associated with increased BCR signaling (Goodnow et al., 1988; Pillai and Cariappa, 2009). Thus, we hypothesized that myosin IIa is a negative regulator of BCR signaling. To study the role of myosin IIa in the regulation of BCR signaling, we stimulated myosin IIa-proficient and myosin IIa-deficient B cells with soluble anti-IgM and found that phosphorylation of Syk, Blnk, and Akt and intracellular calcium fluxes were similar (Figures S5A and S5B), suggesting proximal BCR signaling is unaffected by myosin IIa-deletion. In addition, prolonged stimulation of myosin IIa-deficient cells with soluble BCR ligands resulted in normal upregulation of the activation markers Cd69, Cd86, and MHC class II, albeit to slightly lower levels than in CD23Cre^{+}Myh9$^{wt/fl}$ cells (Figure S5C), suggesting myosin IIa is also not involved in regulating more distal BCR signaling pathways. Next, we analyzed internalization and processing of soluble antigen using DNA-based antigen degradation sensors as described previously (Nowosad et al., 2016). Myosin IIa-deficient B cells were as efficient in internalizing soluble anti-immunoglobulin κ (Igκ) as myosin IIa-proficient cells (Figure S5D), in agreement with previous reports for B cells treated with blebbistatin (Natkanski et al., 2013). Blebbistatin has also been reported to reduce antigen presentation capability of primary B cells and B cell lymphoma cell lines (Vascotto et al., 2007). However, similar levels of MHC class II-bound Ea peptide were detected at the cell surface of CD23Cre^{+}Myh9$^{wt/fl}$ and CD23Cre^{+}Myh9$^{fl/fl}$ B cells after stimulation with anti-Igκ and Ea peptide-loaded microbeads (Figure S5E). Altogether, these findings indicate that myosin IIa is not required for the regulation of BCR signaling or for internalization, processing, and presentation of soluble antigen.

Myosin IIa-Deficient B Cells Are Less Efficient at Extracting Antigen from Membrane Substrates

To investigate the role of myosin IIa in BCR signaling and internalization in response to membrane-presented antigen, we made use of a large-scale imaging approach of primary B cells on antigen-presenting plasma membrane sheets (PMSs) as developed previously (Nowosad et al., 2016). PMSs are flexible membrane substrates that facilitate B cell synapse formation, BCR signaling, and antigen internalization when coated with antigen. After 40 min on anti-Igκ-coated PMSs, myosin IIa-deficient B cells showed significantly less, but not absent, antigen internalization compared to myosin IIa-proficient cells (Figure 3A), showing that myosin IIa is important for efficient acquisition of membrane-bound antigen. To investigate whether the reduced internalization of membrane-bound antigen affects termination of BCR signaling, phosphorylation of BCR signaling pathway components was analyzed in myosin IIa-deficient B cells engaging antigen on PMS. However, we did not observe significant changes in phosphorylation of Blnk, Syk, or Erk (Figures 3B and 3C). Thus, myosin IIa does not regulate BCR signaling under these conditions.

Myosin IIa Is Required for Efficient Migration and *In Vivo* Trafficking of B Cells

Adhesion to ICAM-1 lowers the threshold for B cell activation by promoting synapse formation (Carrasco et al., 2004). To study the adhesive properties of myosin IIa-deficient B cells, we stimulated cells with anti-IgM in the presence of soluble ICAM-1 and analyzed ICAM-1 binding to the cell surface by flow cytometry. In both CD23Cre^{+}Myh9$^{fl/fl}$ and CD23Cre^{+}Myh9$^{fl/wt}$ B cells, binding of ICAM-1 was induced to a similar extent (Figure S6A). Adhesion of anti-IgM or $MnCl_2$-stimulated B cells to immobilized ICAM-1 was also similar between myosin IIa-deficient and myosin IIa-proficient B cells (Figure S6B).

Using time-lapse imaging, we analyzed motility of myosin IIa-deficient B cells on ICAM-1-coated glass in the presence of Cxcl13 and observed reduced crawling speed compared to CD23Cre^{+}Myh9$^{fl/wt}$ B cells (Figure 4A; Video S1). A fraction of myosin IIa-deficient cells developed elongated uropods (Video S1). In Transwell assays, myosin IIa-deficient B cells displayed reduced migration toward Cxcl13, Cxcl12, and Ccl21 (Figure 4B). Coating of Transwell membranes with ICAM-1 facilitated robust migration of myosin IIa-proficient B cells, but not of myosin IIa-deficient B cells. In contrast, coating of Transwell membranes with anti-Igκ, a stronger adhesive, severely reduced

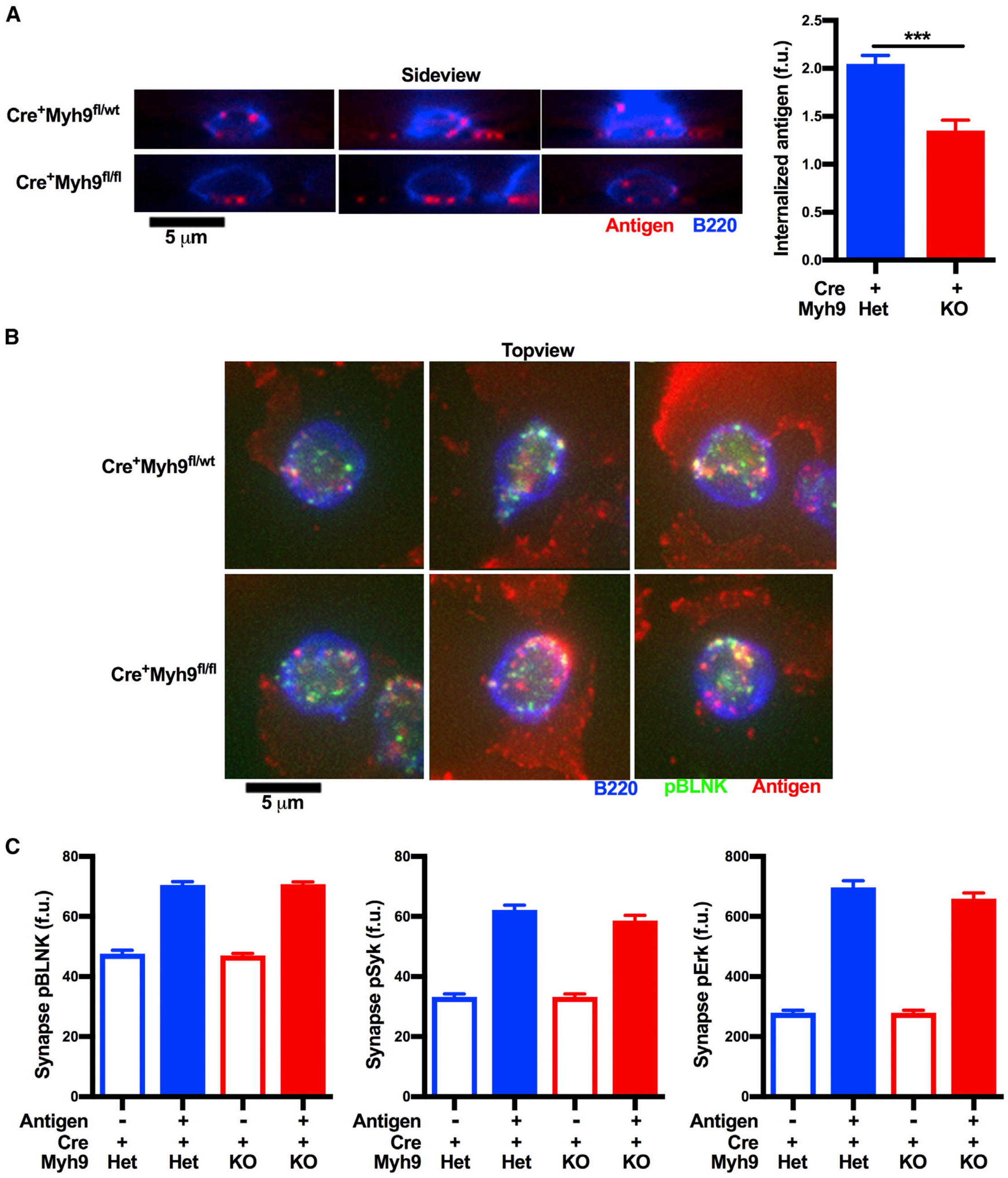

Figure 3. Myosin IIa-Deficient B Cells Are Less Efficient at Internalizing Membrane-Bound Antigen *In Vitro*

(A) Side view reconstructions and quantification of internalized antigen of follicular B cells (blue) from CD23Cre^{+}Myh9$^{wt/fl}$ and CD23Cre^{+}Myh9$^{fl/fl}$ mice after 40 min of interaction with plasma membrane sheet (PMS)-bound antigen (red).

(B) Top view of synapse plane showing pBlnk staining (green) in follicular B cells (blue) from CD23Cre^{+}Myh9$^{wt/fl}$ and CD23Cre^{+}Myh9$^{fl/fl}$ mice after 15 min of interaction with PMS-bound antigen (red).

(C) Quantification of synaptic pBlnk (left), pSyk (middle), and pErk (right) after 20, 20, and 15 min of interaction with PMS, respectively. Cells that landed outside of PMS were used as unstimulated controls.

Mean ± SEM (n > 192 cells). ***p < 0.001 (Mann-Whitney U test). Fu, fluorescence units. See also Figure S5.

migration of CD23Cre^{+}Myh9$^{fl/wt}$ and nearly abolished migration of CD23Cre^{+}Myh9$^{fl/fl}$ B cells. Altogether, these data indicate that B cells require myosin IIa for detachment from adhesive surfaces.

To analyze migration of myosin IIa-deficient B cells *in vivo*, we i.v. injected labeled CD23Cre^{+}Myh9$^{fl/fl}$ and CD23Cre^{+} Myh9$^{fl/wt}$ B cells into C57BL/6J mice and found an increased ratio of myosin IIa-deficient cells in the LNs after

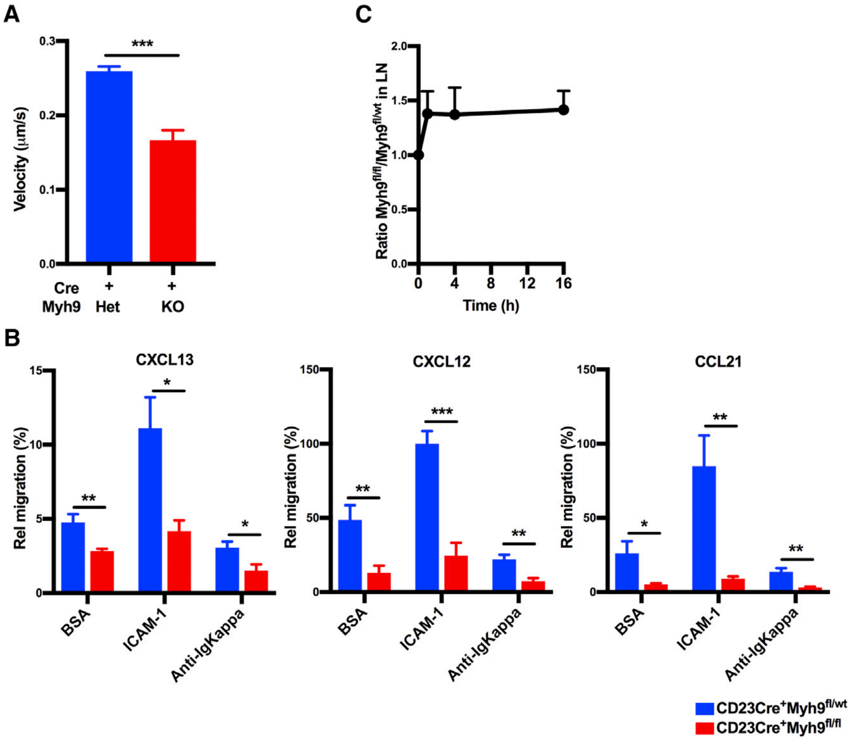

Figure 4. Disturbed Migration and *In Vivo* Trafficking of Myosin IIa-Deficient B Cells

(A) Velocity of CXCL13-activated CD23Cre$^+$ Myh9$^{wt/fl}$ and CD23Cre$^+$Myh9$^{fl/fl}$ B cells on an ICAM-1-coated glass surface ($n > 48$ cells). ***$p < 0.001$ (Mann-Whitney U test).

(B) Directional migration through BSA, ICAM-1, and anti-Igκ-coated Transwells of CD23Cre$^+$Myh9$^{wt/fl}$ and CD23Cre$^+$Myh9$^{fl/fl}$ B cells toward the indicated chemokines. Data are normalized on Cxcl12-mediated migration of CD23Cre$^+$Myh9$^{wt/fl}$ B cells. Mean ± SEM. *$p < 0.05$, **$p < 0.01$, ***$p < 0.001$ (unpaired t test).

(C) Ratio of B cells from CD23Cre$^+$Myh9$^{wt/fl}$ and CD23Cre$^+$Myh9$^{fl/fl}$ mice after i.v. injection into C57BL/6J mice normalized to input ratio ($n = 8$ mice). Mean ± SD. LN, lymph node.

See also Figure S6.

1, 4, and 16 hr (Figure 4C). This skewed ratio of B cells in the LN was not due to facilitated homing of myosin IIa-deficient cells caused by increased L-selectin (CD62L) expression, because we observed normal CD62L expression on myosin IIa-deficient B cells (Figure S6C). To analyze whether myosin IIa-deficient cells migrate normally within LNs, we assessed the ratio of myosin IIa-deficient and myosin IIa-proficient B cells by immunofluorescence staining of cryopreserved LNs 16 hr after transfer and found that the ratio of myosin IIa-proficient and myosin IIa-deficient cells in B cell follicles was similar to the ratio determined by flow cytometry (Figure S6D).

Myosin IIa Is Required for Efficient Acquisition of Antigen *In Vivo*

To investigate the role of myosin IIa in antigen acquisition *in vivo*, CD23Cre$^+$Myh9$^{fl/wt}$ and CD23Cre$^+$Myh9$^{fl/fl}$ mice were crossed with SW$_{HEL}$ mice. B cells of SW$_{HEL}$ mice express a high-affinity anti-hen egg lysozyme (HEL) antibody as a BCR on the cell surface (Phan et al., 2003). In the resulting mice, approximately half of the B cells expressed the HEL-specific BCR, as determined by binding of biotinylated HEL and PE-labeled streptavidin (Figure S7A). A similar percentage of cells could be stained with HEL3x, a modified version of HEL with 10,000-fold lower affinity for the SW$_{HEL}$ BCR (Paus et al., 2006), albeit with lower mean fluorescence intensity (MFI). No difference was found in HEL binding capacity between antigen-specific B cells of CD23Cre$^+$ Myh9$^{fl/wt}$SW$_{HEL}$ and CD23Cre$^+$Myh9$^{fl/fl}$SW$_{HEL}$ mice despite lower surface IgM levels in the latter (Figure S7B), suggesting total surface BCR levels, which also include immunoglobulin D (IgD), were not significantly changed.

To target HEL antigen to FDCs *in vivo*, we adapted a protocol to generate PE-labeled HEL immune complexes in CD45.1 mice by intraperitoneal injection of anti-PE antibody, followed 1 day later by subcutaneous (s.c.) injection of HEL or HEL3x bound to PE-labeled streptavidin near the axillary and inguinal LNs (Phan et al., 2007; Suzuki et al., 2009). Twenty-four hours after generation of immune complexes, when most of the antigen is presented on the surface of FDCs in LNs (Figure S7C), SW$_{HEL}$ B cells were transferred. Fourteen hours after transfer, we isolated B cells from axillary and inguinal LNs and could detect PE-containing immune complexes on approximately 60% of transferred B cells that express the SW$_{HEL}$ BCR. In contrast, only 3% of donor B cells that do not express a HEL-specific BCR were positive for PE (Figure S7D), possibly because of capture of anti-PE-HEL-streptavidin-PE complexes via complement receptor Cr2 (Phan et al., 2007). PE$^+$ B cells had upregulated the activation markers Cd69 and Cd86, demonstrating that the HEL uptake is BCR mediated (Figure S7E). Omitting passive immunization with anti-PE antibody resulted in few PE-positive cells, suggesting that the antigen has been taken up from immune complex binding APCs.

To quantify HEL uptake from APCs *in vivo* in the absence of myosin IIa, B cells from CD23Cre$^+$Myh9$^{fl/wt}$SW$_{HEL}$ and CD23Cre$^+$Myh9$^{fl/fl}$SW$_{HEL}$ were isolated, labeled, and transferred concomitantly into HEL-immunized mice. After 14 hr, we found a modest but significant reduction in HEL bound to myosin IIa-deficient SW$_{HEL}$ B cells (Figure 5A). A similar reduction in antigen uptake by myosin IIa-deficient SW$_{HEL}$ B cells was observed when the lower-affinity HEL3x was used (Figure 5B). HEL-positive CD23Cre$^+$Myh9$^{fl/wt}$SW$_{HEL}$ and CD23Cre$^+$Myh9$^{fl/fl}$SW$_{HEL}$ B cells equally upregulated Cd69 and Cd86 (Figure S7E), demonstrating that access to antigen is similar. We conclude that myosin IIa is a positive regulator of antigen acquisition of membrane-bound antigen *in vivo*.

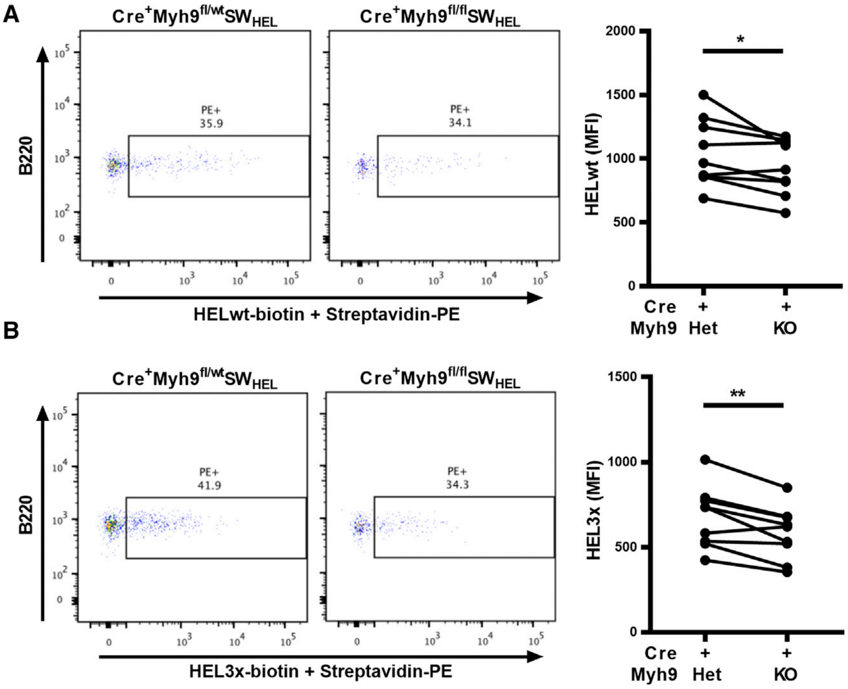

Figure 5. Myosin IIa Is Required for Efficient Acquisition of Low-Affinity Antigen *In Vivo*

(A) Flow cytometric analysis and quantification of HEL-streptavidin-PE complex capture by B220$^+$ cells from CD23Cre$^+$Myh9$^{fl/wt}$SW$_{HEL}$ or CD23Cre$^+$Myh9$^{fl/fl}$SW$_{HEL}$ mice 14 hr after transfer into HEL-streptavidin-PE-immunized CD45.1 mice. Plotted is the MFI of PE$^+$ cells, paired per mouse.

(B) Flow cytometric analysis and quantification of HEL3x-streptavidin-PE complex capture by B220$^+$ cells from CD23Cre$^+$Myh9$^{fl/wt}$SW$_{HEL}$ or CD23Cre$^+$Myh9$^{fl/fl}$SW$_{HEL}$ mice 14 hr after transfer into HEL3x-streptavidin-PE-immunized CD45.1 mice. Plotted is the MFI of PE$^+$ cells, paired per mouse.

Flow cytometry plots display data of a representative experiment. *$p < 0.05$, **$p < 0.01$ (paired t test). See also Figure S7.

Myosin IIa-Deficient B Cells Have a Defect in Cytokinesis

Past experiments with blebbistatin in HeLa and Cos-7 cells demonstrated that class II myosins cooperate to separate daughter cells during cytokinesis, the final step of the cell cycle (Ma et al., 2012; Straight et al., 2003). Because myosin IIa is the only class II myosin expressed in lymphocytes and both the pro-B cell stage and the GC are sites of extensive B cell proliferation, we hypothesized that myosin IIa-deficient B cells may have a defect in cytokinesis. To investigate proliferation of myosin IIa-deficient B cells, cells were labeled and cultured in the presence of various stimuli. After 48 hr, up to 40% of CD23Cre$^+$Myh9$^{fl/wt}$ follicular B cells stimulated with lipopolysaccharide (LPS), CpG, or Cd40lg and interleukin-4 (IL-4) had divided, as determined by dilution of dye (Figure 6A). In contrast, less than 10% of CD23Cre$^+$Myh9$^{fl/fl}$ B cells had proliferated regardless of the stimulus. CpG-stimulated myosin IIa-deficient B cells were markedly enlarged (Figure 6B), in agreement with a defect in cytokinesis. Pro-B cells of Mb1Cre$^+$Myh9$^{fl/fl}$ mice were larger than myosin IIa-proficient counterparts in Mb1Cre$^+$Myh9$^{fl/wt}$ mice (Figure 6C), suggesting these cells may have failed cytokinesis *in vivo*.

When stimulated with CpG for 48 hr *in vitro*, a higher proportion of myosin IIa-deficient B cells were in G2 or M phase, harboring 4N of DNA, as determined by propidium iodide incorporation, and indicating a block in a late phase of the cell cycle (Figures 6D and 6E). Moreover, CpG stimulation of myosin IIa-deficient B cells resulted in a large fraction of cells containing more than 4N of DNA, suggesting these cells failed to complete cytokinesis and entered the cell cycle again. Thus, myosin IIa is essential for B cell proliferation, explaining the block of B cell development and loss of GC cells *in vivo*.

Myosin IIa Is Essential for Antibody Responses

Collectively, the cytokinesis defect, the reduced levels of MZ and GC B cells, and the reduced capacity to acquire antigen could result in disrupted antibody production. CD23Cre$^+$Myh9$^{fl/fl}$ mice had diminished levels of steady-state serum IgM and immunoglobulin G (IgG) 1, IgG2b, and IgG3 (Figure 7A). To study the role of myosin IIa in antibody responses, we transferred BM from CD23Cre$^+$Myh9$^{fl/wt}$ and CD23Cre$^+$Myh9$^{fl/fl}$ mice into *Rag1*-KO mice. After reconstitution, mice were immunized with the T-dependent antigen 4-hydroxy-3-nitrophenylacetyl-chicken gamma globulin (NP-CGG), and NP-specific antibodies in the serum were analyzed after 7 and 10 days. NP-specific IgG1 antibodies were not detected in CD23Cre$^+$Myh9$^{fl/fl}$-reconstituted mice, whereas a clear induction of NP-specific IgG1 antibodies was found in CD23Cre$^+$Myh9$^{fl/wt}$-reconstituted mice (Figure 7B). Some NP-specific IgM could be detected in immunized CD23Cre$^+$Myh9$^{fl/fl}$-reconstituted mice, although at lower levels than in immunized CD23Cre$^+$Myh9$^{fl/wt}$-reconstituted mice. When immunized with the T-independent antigen NP-Ficoll, CD23Cre$^+$Myh9$^{fl/fl}$-reconstituted mice did not generate detectable levels of NP-specific IgG3 (Figure 7C). A small induction of NP-specific IgM could be detected but was significantly less than in CD23Cre$^+$Myh9$^{fl/wt}$-reconstituted mice. We conclude that myosin IIa is essential for mounting efficient B cell antibody responses.

DISCUSSION

B cell antigen acquisition from the surface of APCs is increasingly recognized as an important step in B cell responses (Cyster, 2010). Previous *in vitro* genetic and pharmacological perturbations identified myosin IIa contractility as an important factor in antigen extraction and delivery into MHC class II-containing processing compartments (Natkanski et al., 2013; Vascotto et al., 2007). Here, using mice in which myosin IIa was conditionally or inducibly deleted from mature B cells, we demonstrate that myosin IIa is important for antigen acquisition from FDCs *in vivo*.

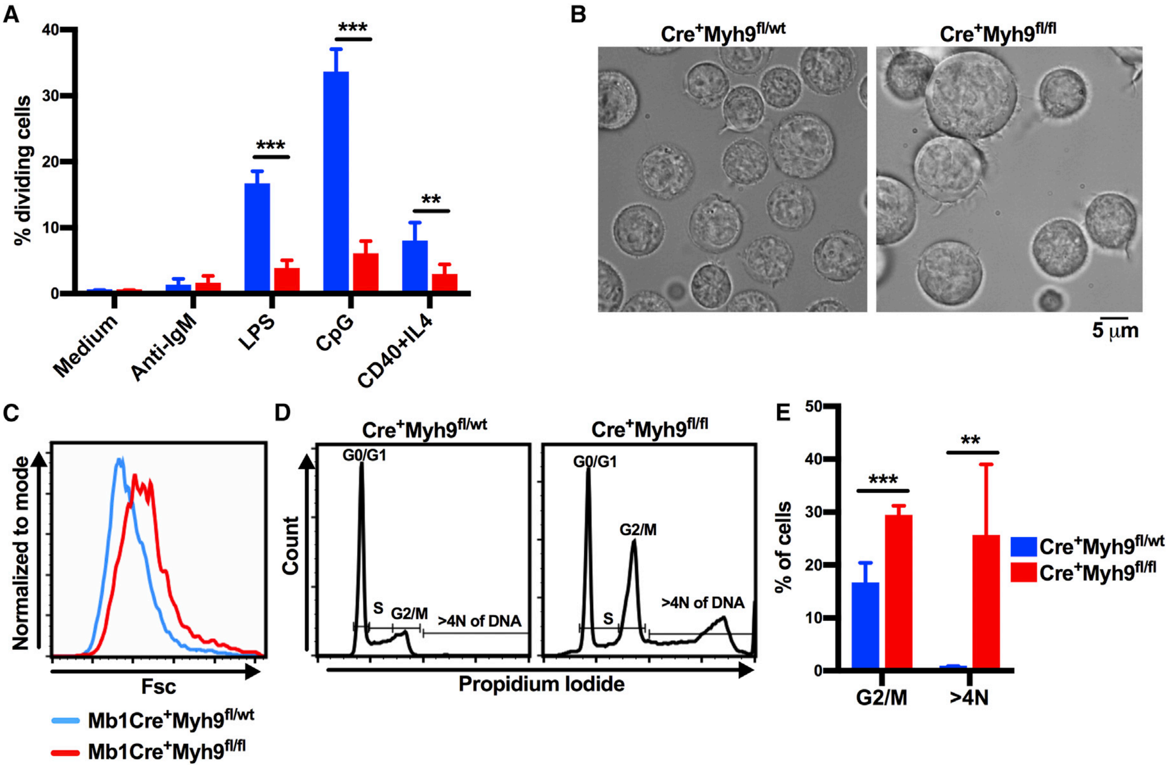

Figure 6. Myosin IIa-Deficient B Cells Have a Defect in Cytokinesis

(A) Percentage of CD23Cre^{+}Myh9$^{wt/fl}$ and CD23Cre^{+}Myh9$^{fl/fl}$ B cells that have divided after 48 hr of cell culture in the presence of indicated stimuli.
(B) Light microscopy images of CD23Cre^{+}Myh9$^{wt/fl}$ and CD23Cre^{+}Myh9$^{fl/fl}$ B cells stimulated with CpG for 48 hr.
(C) Size of BM B220^{+}CD19^{+}CD2^{-}IgM^{-} pro-B cells of Mb1Cre^{+}Myh9$^{wt/fl}$ and Mb1Cre^{+}Myh9$^{fl/fl}$ mice.
(D) Flow cytometric analysis of propidium iodide incorporation of CD23Cre^{+}Myh9$^{wt/fl}$ and CD23Cre^{+}Myh9$^{fl/fl}$ B cells stimulated with CpG for 48 hr.
(E) Quantification of cells in G2/M phase and cells with more than 4N of DNA after 48 hr of CpG stimulation.
Means ± SD. $^{**}p < 0.01$, $^{***}p < 0.001$ (unpaired t test).

This is consistent with our discovery that FDCs have stiff membranes compared to other APCs, requiring application of strong forces by antigen-extracting B cells (Spillane and Tolar, 2017). We have also found that GC B cells have increased force generation capability, supporting that strong forces are required for antigen extraction in the GC (Nowosad et al., 2016). However, the rapid and complete disappearance of myosin IIa-deficient GC B cells, most likely due to a cytokinesis defect, prevented us from analyzing these cells in this study.

The extraction of membrane-bound antigen was only reduced by approximately 50% after genetic deletion of myosin IIa, in contrast to the 80%–90% reported with blebbistatin inhibition (Natkanski et al., 2013; Nowosad et al., 2016). This milder effect of myosin IIa deletion on antigen uptake prevented us from assessing the potential effects of cellular mechanics on antigen affinity discrimination in this model. It is possible that genetic deletion of myosin IIa can be compensated by other molecular motors or that blebbistatin has off-target effects that further diminish force generation by B cells. Differences between genetic inactivation of myosin IIa and inhibition by blebbistatin have also been observed when studying cytokinesis (Ma et al., 2012). Along these lines, we found that myosin IIa was not required for processing and presentation of soluble antigens, as reported previously (Vascotto et al., 2007). Possibly, alternative compensatory mechanisms take over this process after long-term genetic deletion of myosin IIa. This suggests caution is required when interpreting inhibitor or genetic studies in isolation.

When we analyzed antigen acquisition by myosin IIa-deficient B cells from APCs *in vivo*, we found that it was only modestly reduced, suggesting that substantial antigen extraction can be achieved without specialized myosin IIa-dependent pulling activity in the immune synapse. Again, deletion of myosin IIa may have been compensated by other molecular motors. In addition, the disturbed migration of myosin IIa-deficient B cells may facilitate antigen uptake *in vivo* due to prolonged interaction with the APC. Further studies on the role of cellular motility in B cell antigen acquisition should resolve this question.

Our analysis of myosin IIa-deficient B cells also shows that myosin IIa is important for the development of MZ and B1b B cells and negatively regulates the activation of follicular B cells. A loss of MZ B cells, combined with an activated phenotype of follicular B cells, has previously been reported in mice lacking negative regulators of BCR signaling, such as *Cd22* and *Aiolos* (Cariappa et al., 2001; Nitschke et al., 1997; Sato et al., 1996), and in transgenic mice expressing a self-reactive BCR (Cooke et al., 1994; Goodnow et al., 1988) or overexpressing *Cd19*

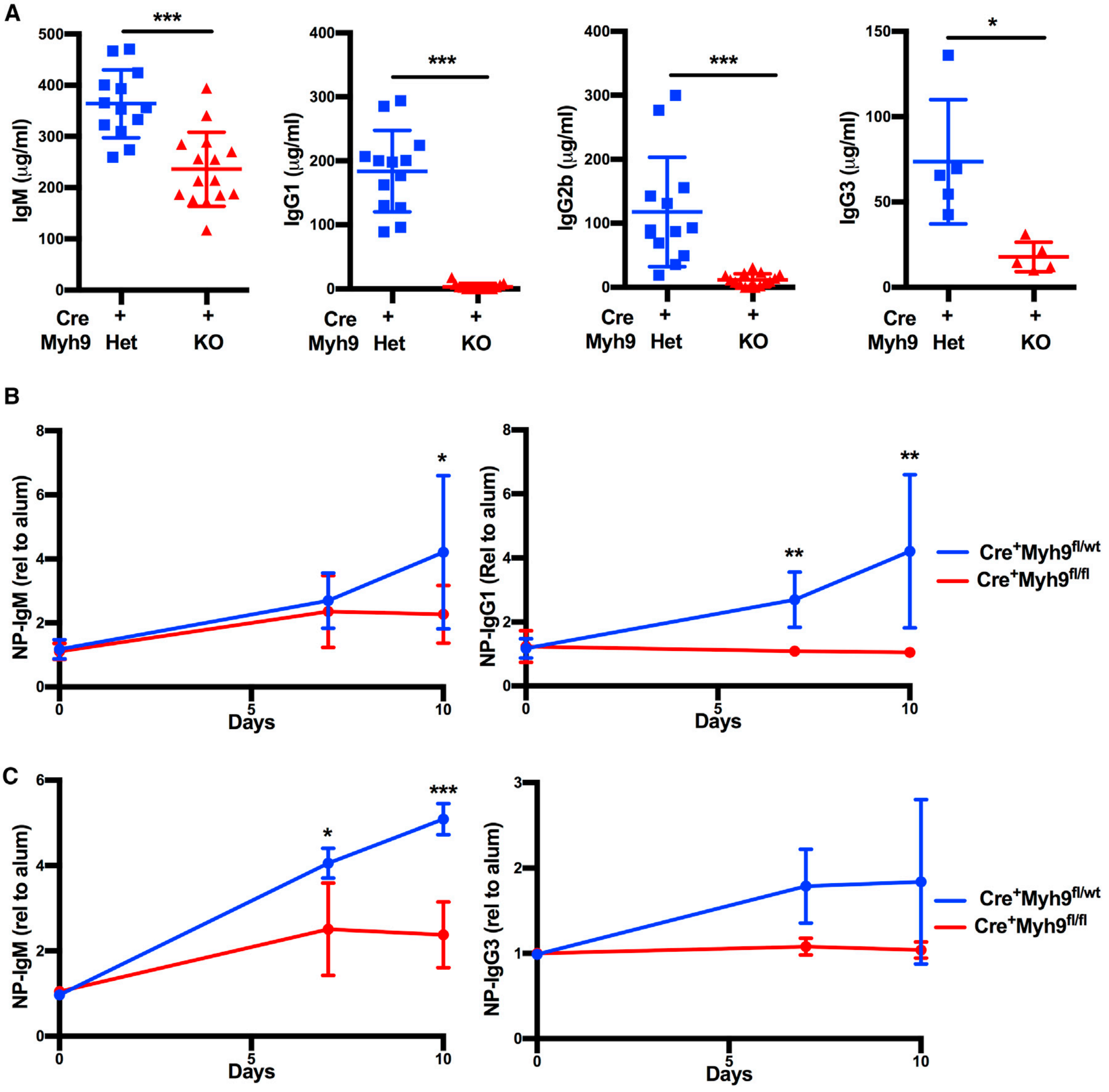

Figure 7. Myosin IIa Is Essential for Antibody Responses

(A) Serum immunoglobulin levels of CD23Cre^{+}Myh9$^{fl/wt}$ and CD23Cre^{+}Myh9$^{fl/fl}$ mice.

(B) NP-specific immunoglobulin levels of CD23Cre^{+}Myh9$^{fl/wt}$ and CD23Cre^{+}Myh9$^{fl/fl}$ BM-reconstituted mice after immunization with the T-dependent antigen NP-CGG (n = 10).

(C) NP-specific immunoglobulin levels of CD23Cre^{+}Myh9$^{fl/wt}$ and CD23Cre^{+}Myh9$^{fl/fl}$ BM-reconstituted mice after immunization with the T-independent antigen NP-Ficoll (n = 5).

Dots represent individual mice analyzed in two experiments. Horizontal bars reflect mean ± SD. *$p < 0.05$, **$p < 0.01$, ***$p < 0.001$ (unpaired t test).

(Tedder et al., 1997). This suggests that myosin IIa is a negative regulator of BCR signaling.

Sustained BCR signaling may render B cells anergic *in vivo* (Cooke et al., 1994; Goodnow et al., 1988). Although myosin IIa-deficient B cells responded normally to antigen stimulation, we observed a reduced capacity to upregulate Cd86, a feature previously attributed to anergic B cells (Benschop et al., 2001; Rathmell et al., 1998), suggesting myosin IIa-deficient B cells may have become partly anergic. Anergic B cells are short lived (Santulli-Marotto et al., 1998), which may explain the reduced numbers of follicular B cells in competitive BM chimeras. Hyper-responsiveness of follicular B cells has also been described in mice in which other components of the B cell cytoskeleton or BCR internalization or trafficking machinery were deleted, such as *Was*, *Dbnl*, or *Cbl*, often resulting in a mild autoimmune phenotype (Becker-Herman et al., 2011; Kitaura et al., 2007; Seeley-Fallen et al., 2014; Song et al., 2013). However, follicular B cells in these mice did not develop a hyperactivated surface phenotype, suggesting that myosin IIa may regulate B cell activation by a distinct mechanism. Possibly, myosin IIa may regulate BCR signaling under specific conditions, such as signaling induced by antigen presented on APCs or during a distinct developmental step. In support,

transgenic expression of antigen on FDCs resulted in reduced IgM and increased MHC class II on antigen-specific naive follicular B cells (Yau et al., 2013), suggesting that BCR signaling induced by FDC-presented ligands in the spleen could lead to an activated phenotype similar to that observed in mice with B cell-specific deletion of myosin IIa. However, the exact nature of the signaling pathway activated in the myosin IIa-deficient B cells remains to be identified, because we did not observe an obvious increase in canonical BCR signaling induced by either soluble or membrane-bound antigens.

Analysis of adhesion *in vitro* showed that myosin IIa-deficient B cells adhered normally to soluble or immobilized ICAM-1. Moreover, when co-stimulated with both membrane-bound antigen and immobilized ICAM-1, BCR signaling was similar in myosin IIa-deficient and myosin IIa-proficient B cells (data not shown), demonstrating that initial attachment to ICAM-1 is normal. However, time-lapse imaging and Transwell experiments demonstrated that B cells require myosin IIa for detachment from adhesive substrates, similar to what has been described for myosin IIa-deficient T cells (Jacobelli et al., 2010; Morin et al., 2008). We speculate that this defect in detachment from adhesive substrates may result in prolonged interaction with antigen on APCs and thus in sustained BCR signaling *in vivo*. *Lsc*-deficient mice, which also have a defect in ICAM-1 detachment, harbor reduced MZ B cell numbers and follicular B cells with a hyperactivated phenotype (Girkontaite et al., 2001; Rubtsov et al., 2005), partly recapitulating the phenotype of mice with myosin IIa-deleted B cells described here.

Although the loss of MZ B cells after myosin IIa deletion is consistent with sustained BCR signaling, at least three other signals that are required for MZ B cell development could be potentially involved in this phenotype (Pillai and Cariappa, 2009). First, MZ B cell development needs Notch2 signaling. Interaction of Notch2 with its ligand Dll1 initiates cleavage of Notch2 by Adam10 (Gibb et al., 2010), an event that could rely on myosin IIa-mediated Adam10 transport, force generation, or cell tension. However, we measured normal Adam10 surface translocation after BCR stimulation and normal upregulation of Notch target genes in myosin IIa-deficient B cells, suggesting myosin IIa does not influence Notch signaling. Second, MZ B cell development depends on canonical nuclear factor κB (NF-κB) signaling, most likely induced by Tnfsf13b (BAFF). However, genetic disruption of canonical NF-κB signaling does not result in upregulation of activation markers. Moreover, *in vitro* survival in the presence of BAFF was normal in myosin IIa-deficient B cells (data not shown), suggesting myosin IIa is also not required for overall regulation of BAFF signaling. Finally, MZ B cell development and maintenance require migration and subsequent retention in the MZ (Lu and Cyster, 2002), which could depend on myosin IIa. However, we found increased labeling of myosin IIa-deficient MZ B cells after injection of anti-Cd19 antibody, indicating that more, not fewer, myosin IIa-deficient MZ B cells are localized in the MZ and red pulp. Possibly, a decrease in myosin IIa-dependent cellular locomotion may result in less shuttling of MZ B cells between the MZ and the follicle.

MZ B cells could also suffer from the observed cytokinesis defect, although proliferation of MZ B cells during steady-state conditions is generally very low and has, to our knowledge, only been reported when BM B cell development is blocked (Hao and Rajewsky, 2001). It is conceivable that declining numbers of MZ B cells at some point induce proliferation of remaining MZ B cells, thereby accelerating loss of MZ B cells through the defect in proliferation. However, loss of myosin IIa-deficient MZ B cells was also observed in the presence of wild-type MZ B cells in competitive BM chimeras, demonstrating that the initial loss of MZ B cells is cell intrinsic.

Overall, our data show that myosin IIa is required for efficient antibody responses, which is likely a result of the combined effects on antigen acquisition and proliferation. It is probable that because of these effects, myosin IIa-deficient B cells were unable to contribute to autoimmune reactions despite their activated phenotype, in contrast to the reported autoimmune syndromes in other mice with disturbed BCR internalization and trafficking (Becker-Herman et al., 2011; Kitaura et al., 2007; Song et al., 2013). Myosin IIa has been identified as a tumor suppressor by cytokinesis regulation or by post-transcriptional regulation of p53 (Conti et al., 2015; Schramek et al., 2014). We found several p53 target genes downregulated in myosin IIa-deficient follicular B cells, suggesting p53 function may also be deregulated in myosin IIa-deficient follicular B cells. However, whereas p53 null mice develop spontaneous lymphomas (Harvey et al., 1995), it is likely that transformation of myosin IIa-deficient B cells is again limited by the proliferation defect described in this paper.

Our data thus establish a critical role of myosin contractility in multiple aspects of B cell biology and should open new avenues to study the role of the cytoskeleton and B cell intrinsic force generation in antibody responses.

EXPERIMENTAL PROCEDURES

Mice

Myh9$^{fl/fl}$ (Myh9^{tm5Rsad}) mice were crossed with Mb1Cre (Cd79a$^{tm1(cre)Reth}$), CD23Cre (Tg(Fcer2a-cre)5Mbu), and R26ERT2Cre (Gt(ROSA) 26Sor$^{tm1(cre/ERT2)Thl}$) mice as described previously (de Luca et al., 2005; Hobeika et al., 2006; Jacobelli et al., 2010; Kwon et al., 2008). CD23CreMyh9$^{fl/fl}$ mice were crossed with SW$_{HEL}$ (Igh^{tm1Rbr}-Tg(IgkHyHEL10)1Rbr) mice (Phan et al., 2003). 6–12 week old male and female mice were used for *ex vivo* analysis of cell populations. For *in vitro* experiments, cells isolated from 6–20 week old mice were used.

To generate BM transfer or chimera mice, *Rag1*-deficient mice (Rag1^{tm1Mom}) were irradiated with 5 Gy and reconstituted with BM cells by i.v. injection. R26ERT2Cre^{+}Myh9 mice received 2 mg/day of tamoxifen (Sigma) suspended in corn oil by intraperitoneal injection for 5 days. When indicated, BM was mixed with 80% muMT (Ighm^{tm1Cgn}) or CD45.1 (B6.SJL-Ptprc^{a}/Nimr). Mice were bred and kept in accordance with guidelines set by the UK Home Office and the Francis Crick Institute Ethical Review Panel.

Large-Scale Imaging

Generation of PMS and the large-scale imaging approach have been described previously (Natkanski et al., 2013; Nowosad et al., 2016). A brief description is provided in Supplemental Experimental Procedures.

Antigen Acquisition

To study *in vivo* antigen acquisition, we adapted a protocol to generate HEL-containing immune complexes as described previously (Phan et al., 2007). CD45.1 mice were passively immunized with a polyclonal anti-PE antibody (Rockland Immunochemicals). The next day, 10 μg of biotinylated HEL or HEL3x protein, produced in house as described (Paus et al., 2006), was complexed to PE-labeled streptavidin (BioLegend) and injected s.c. near the inguinal and axillary LNs under isoflurane anesthesia. The following day,

CD23Cre$^+$Myh9$^{fl/wt}$SW$_{HEL}$ and CD23Cre$^+$Myh9$^{fl/fl}$SW$_{HEL}$ B cells were isolated, fluorescently labeled, mixed, and transferred to immunized recipient mice by i.v. injection. After 14 hr, inguinal and axillary LNs were harvested and analyzed by flow cytometry.

In vitro internalization and presentation of soluble antigen are described in Supplemental Experimental Procedures.

Adhesion and Migration

To investigate *in vivo* homing and migration, B cells were labeled with 5-chloromethylfluorescein diacetate (CMFDA) and CellTrace Far Red (CTFR) and mixed in a 1:1 ratio, and 20 × 10^6 cells were i.v. injected into C57BL/6J recipient mice. At indicated times, LNs were collected and the ratio of donor cells was determined by flow cytometry or immunofluorescence microscopy of cryosections.

To determine localization of myosin IIa-deficient MZ B cells, R26ERT2Cre$^+$ Myh9 muMT BM chimeras were treated with tamoxifen as described earlier. 14 days after the first tamoxifen injection, mice received 1 μg anti-Cd19-PE antibody by i.v. injection. Mice were culled 5 min later, splenocytes were harvested on ice, and cells were analyzed by flow cytometry. *In vitro* migration and adhesion experiments are described in Supplemental Experimental Procedures.

Flow Cytometry, Signaling, Activation, and Proliferation

B cell populations, signaling, activation, proliferation, and gene expression were analyzed using standard techniques described in Supplemental Experimental Procedures.

Statistical Analysis

Statistical analyses were performed using GraphPad Prism 7 software. For each experiment, the statistical test used, the sample size, and the statistical significance are included in the figure legend.

DATA AND SOFTWARE AVAILABILITY

The accession number for the data from transcriptomic analysis reported in this paper is GEO: GSE113114.

SUPPLEMENTAL INFORMATION

Supplemental Information includes Supplemental Experimental Procedures, seven figures, one table, and one video and can be found with this article online at https://doi.org/10.1016/j.celrep.2018.04.087.

ACKNOWLEDGMENTS

We acknowledge the Francis Crick flow cytometry and advanced sequencing facilities for cell sorting and RNA-seq, respectively. The work was supported by the Netherlands Scientific Organization (Rubicon grant 825.13.013 to R.H.), the European Research Council (Consolidator Grant 648228 to P.T.), the EMBO Young Investigator Programme (P.T.), and the Francis Crick Institute, which receives its funding from Cancer Research UK, the UK Medical Research Council, and the Wellcome Trust.

AUTHOR CONTRIBUTIONS

R.H. and P.T. designed the research; R.H., E.M.N., C.R.N., and D.M. performed the research; R.P.M. and A.C. generated reagents; and R.H. and P.T. wrote the paper.

DECLARATION OF INTERESTS

The authors declare no competing interests.

Received: August 16, 2017
Revised: March 23, 2018
Accepted: April 19, 2018
Published: May 22, 2018

REFERENCES

Becker-Herman, S., Meyer-Bahlburg, A., Schwartz, M.A., Jackson, S.W., Hudkins, K.L., Liu, C., Sather, B.D., Khim, S., Liggitt, D., Song, W., et al. (2011). WASp-deficient B cells play a critical, cell-intrinsic role in triggering autoimmunity. J. Exp. Med. *208*, 2033–2042.

Benschop, R.J., Aviszus, K., Zhang, X., Manser, T., Cambier, J.C., and Wysocki, L.J. (2001). Activation and anergy in bone marrow B cells of a novel immunoglobulin transgenic mouse that is both hapten specific and autoreactive. Immunity *14*, 33–43.

Cariappa, A., Tang, M., Parng, C., Nebelitskiy, E., Carroll, M., Georgopoulos, K., and Pillai, S. (2001). The follicular versus marginal zone B lymphocyte cell fate decision is regulated by Aiolos, Btk, and CD21. Immunity *14*, 603–615.

Carrasco, Y.R., and Batista, F.D. (2007). B cells acquire particulate antigen in a macrophage-rich area at the boundary between the follicle and the subcapsular sinus of the lymph node. Immunity *27*, 160–171.

Carrasco, Y.R., Fleire, S.J., Cameron, T., Dustin, M.L., and Batista, F.D. (2004). LFA-1/ICAM-1 interaction lowers the threshold of B cell activation by facilitating B cell adhesion and synapse formation. Immunity *20*, 589–599.

Conti, M.A., Even-Ram, S., Liu, C., Yamada, K.M., and Adelstein, R.S. (2004). Defects in cell adhesion and the visceral endoderm following ablation of nonmuscle myosin heavy chain II-A in mice. J. Biol. Chem. *279*, 41263–41266.

Conti, M.A., Saleh, A.D., Brinster, L.R., Cheng, H., Chen, Z., Cornelius, S., Liu, C., Ma, X., Van Waes, C., and Adelstein, R.S. (2015). Conditional deletion of nonmuscle myosin II-A in mouse tongue epithelium results in squamous cell carcinoma. Sci. Rep. *5*, 14068–14069.

Cooke, M.P., Heath, A.W., Shokat, K.M., Zeng, Y., Finkelman, F.D., Linsley, P.S., Howard, M., and Goodnow, C.C. (1994). Immunoglobulin signal transduction guides the specificity of B cell-T cell interactions and is blocked in tolerant self-reactive B cells. J. Exp. Med. *179*, 425–438.

Cyster, J.G. (2010). B cell follicles and antigen encounters of the third kind. Nat. Immunol. *11*, 989–996.

de Luca, C., Kowalski, T.J., Zhang, Y., Elmquist, J.K., Lee, C., Kilimann, M.W., Ludwig, T., Liu, S.-M., and Chua, S.C., Jr. (2005). Complete rescue of obesity, diabetes, and infertility in db/db mice by neuron-specific LEPR-B transgenes. J. Clin. Invest. *115*, 3484–3493.

Gibb, D.R., El Shikh, M., Kang, D.J., Rowe, W.J., El Sayed, R., Cichy, J., Yagita, H., Tew, J.G., Dempsey, P.J., Crawford, H.C., and Conrad, D.H. (2010). ADAM10 is essential for Notch2-dependent marginal zone B cell development and CD23 cleavage *in vivo*. J. Exp. Med. *207*, 623–635.

Girkontaite, I., Missy, K., Sakk, V., Harenberg, A., Tedford, K., Pötzel, T., Pfeffer, K., and Fischer, K.D. (2001). Lsc is required for marginal zone B cells, regulation of lymphocyte motility and immune responses. Nat. Immunol. *2*, 855–862.

Gonzalez, S.F., Lukacs-Kornek, V., Kuligowski, M.P., Pitcher, L.A., Degn, S.E., Kim, Y.-A., Cloninger, M.J., Martinez-Pomares, L., Gordon, S., Turley, S.J., and Carroll, M.C. (2010). Capture of influenza by medullary dendritic cells via SIGN-R1 is essential for humoral immunity in draining lymph nodes. Nat. Immunol. *11*, 427–434.

Goodnow, C.C., Crosbie, J., Adelstein, S., Lavoie, T.B., Smith-Gill, S.J., Brink, R.A., Pritchard-Briscoe, H., Wotherspoon, J.S., Loblay, R.H., Raphael, K., et al. (1988). Altered immunoglobulin expression and functional silencing of self-reactive B lymphocytes in transgenic mice. Nature *334*, 676–682.

Hammad, H., Vanderkerken, M., Pouliot, P., Deswarte, K., Toussaint, W., Vergote, K., Vandersarren, L., Janssens, S., Ramou, I., Savvides, S.N., et al. (2017). Transitional B cells commit to marginal zone B cell fate by Taok3-mediated surface expression of ADAM10. Nat. Immunol. *18*, 313–320.

Hao, Z., and Rajewsky, K. (2001). Homeostasis of peripheral B cells in the absence of B cell influx from the bone marrow. J. Exp. Med. *194*, 1151–1164.

Harvey, M., Vogel, H., Morris, D., Bradley, A., Bernstein, A., and Donehower, L.A. (1995). A mutant p53 transgene accelerates tumour development in heterozygous but not nullizygous p53-deficient mice. Nat. Genet. *9*, 305–311.

Hobeika, E., Thiemann, S., Storch, B., Jumaa, H., Nielsen, P.J., Pelanda, R., and Reth, M. (2006). Testing gene function early in the B cell lineage in mb1-cre mice. Proc. Natl. Acad. Sci. USA *103*, 13789–13794.

Jacobelli, J., Friedman, R.S., Conti, M.A., Lennon-Duménil, A.-M., Piel, M., Sorensen, C.M., Adelstein, R.S., and Krummel, M.F. (2010). Confinement-optimized three-dimensional T cell amoeboid motility is modulated via myosin IIA-regulated adhesions. Nat. Immunol. *11*, 953–961.

Junt, T., Moseman, E.A., Iannacone, M., Massberg, S., Lang, P.A., Boes, M., Fink, K., Henrickson, S.E., Shayakhmetov, D.M., Di Paolo, N.C., et al. (2007). Subcapsular sinus macrophages in lymph nodes clear lymph-borne viruses and present them to antiviral B cells. Nature *450*, 110–114.

Kitaura, Y., Jang, I.K., Wang, Y., Han, Y.-C., Inazu, T., Cadera, E.J., Schlissel, M., Hardy, R.R., and Gu, H. (2007). Control of the B cell-intrinsic tolerance programs by ubiquitin ligases Cbl and Cbl-b. Immunity *26*, 567–578.

Kolega, J. (1998). Cytoplasmic dynamics of myosin IIA and IIB: spatial 'sorting' of isoforms in locomoting cells. J. Cell Sci. *111*, 2085–2095.

Kumari, S., Vardhana, S., Cammer, M., Curado, S., Santos, L., Sheetz, M.P., and Dustin, M.L. (2012). T lymphocyte myosin IIA is required for maturation of the immunological synapse. Front. Immunol. *3*, 230.

Kwon, K., Hutter, C., Sun, Q., Bilic, I., Cobaleda, C., Malin, S., and Busslinger, M. (2008). Instructive role of the transcription factor E2A in early B lymphopoiesis and germinal center B cell development. Immunity *28*, 751–762.

Lu, T.T., and Cyster, J.G. (2002). Integrin-mediated long-term B cell retention in the splenic marginal zone. Science *297*, 409–412.

Ma, X., Kovács, M., Conti, M.A., Wang, A., Zhang, Y., Sellers, J.R., and Adelstein, R.S. (2012). Nonmuscle myosin II exerts tension but does not translocate actin in vertebrate cytokinesis. Proc. Natl. Acad. Sci. USA *109*, 4509–4514.

Morin, N.A., Oakes, P.W., Hyun, Y.-M., Lee, D., Chin, Y.E., King, M.R., Springer, T.A., Shimaoka, M., Tang, J.X., Reichner, J.S., and Kim, M. (2008). Nonmuscle myosin heavy chain IIA mediates integrin LFA-1 de-adhesion during T lymphocyte migration. J. Exp. Med. *205*, 195–205.

Murrell, M., Oakes, P.W., Lenz, M., and Gardel, M.L. (2015). Forcing cells into shape: the mechanics of actomyosin contractility. Nat. Rev. Mol. Cell Biol. *16*, 486–498.

Natkanski, E., Lee, W.-Y., Mistry, B., Casal, A., Molloy, J.E., and Tolar, P. (2013). B cells use mechanical energy to discriminate antigen affinities. Science *340*, 1587–1590.

Niiro, H., and Clark, E.A. (2002). Regulation of B-cell fate by antigen-receptor signals. Nat. Rev. Immunol. *2*, 945–956.

Nitschke, L., Carsetti, R., Ocker, B., Köhler, G., and Lamers, M.C. (1997). CD22 is a negative regulator of B-cell receptor signalling. Curr. Biol. *7*, 133–143.

Nowosad, C.R., Spillane, K.M., and Tolar, P. (2016). Germinal center B cells recognize antigen through a specialized immune synapse architecture. Nat. Immunol. *17*, 870–877.

Paus, D., Phan, T.G., Chan, T.D., Gardam, S., Basten, A., and Brink, R. (2006). Antigen recognition strength regulates the choice between extrafollicular plasma cell and germinal center B cell differentiation. J. Exp. Med. *203*, 1081–1091.

Phan, T.G., Amesbury, M., Gardam, S., Crosbie, J., Hasbold, J., Hodgkin, P.D., Basten, A., and Brink, R. (2003). B cell receptor-independent stimuli trigger immunoglobulin (Ig) class switch recombination and production of IgG autoantibodies by anergic self-reactive B cells. J. Exp. Med. *197*, 845–860.

Phan, T.G., Grigorova, I., Okada, T., and Cyster, J.G. (2007). Subcapsular encounter and complement-dependent transport of immune complexes by lymph node B cells. Nat. Immunol. *8*, 992–1000.

Pillai, S., and Cariappa, A. (2009). The follicular versus marginal zone B lymphocyte cell fate decision. Nat. Rev. Immunol. *9*, 767–777.

Qi, H., Egen, J.G., Huang, A.Y.C., and Germain, R.N. (2006). Extrafollicular activation of lymph node B cells by antigen-bearing dendritic cells. Science *312*, 1672–1676.

Rathmell, J.C., Fournier, S., Weintraub, B.C., Allison, J.P., and Goodnow, C.C. (1998). Repression of B7.2 on self-reactive B cells is essential to prevent proliferation and allow Fas-mediated deletion by CD4(+) T cells. J. Exp. Med. *188*, 651–659.

Rosenblatt, J., Cramer, L.P., Baum, B., and McGee, K.M. (2004). Myosin II-dependent cortical movement is required for centrosome separation and positioning during mitotic spindle assembly. Cell *117*, 361–372.

Rubtsov, A., Strauch, P., Digiacomo, A., Hu, J., Pelanda, R., and Torres, R.M. (2005). Lsc regulates marginal-zone B cell migration and adhesion and is required for the IgM T-dependent antibody response. Immunity *23*, 527–538.

Santulli-Marotto, S., Retter, M.W., Gee, R., Mamula, M.J., and Clarke, S.H. (1998). Autoreactive B cell regulation: peripheral induction of developmental arrest by lupus-associated autoantigens. Immunity *8*, 209–219.

Sato, S., Miller, A.S., Inaoki, M., Bock, C.B., Jansen, P.J., Tang, M.L.K., and Tedder, T.F. (1996). CD22 is both a positive and negative regulator of B lymphocyte antigen receptor signal transduction: altered signaling in CD22-deficient mice. Immunity *5*, 551–562.

Schramek, D., Sendoel, A., Segal, J.P., Beronja, S., Heller, E., Oristian, D., Reva, B., and Fuchs, E. (2014). Direct *in vivo* RNAi screen unveils myosin IIa as a tumor suppressor of squamous cell carcinomas. Science *343*, 309–313.

Seeley-Fallen, M.K., Liu, L.J., Shapiro, M.R., Onabajo, O.O., Palaniyandi, S., Zhu, X., Tan, T.H., Upadhyaya, A., and Song, W. (2014). Actin-binding protein 1 links B-cell antigen receptors to negative signaling pathways. Proc. Natl. Acad. Sci. USA *111*, 9881–9886.

Song, W., Liu, C., Seeley-Fallen, M.K., Miller, H., Ketchum, C., and Upadhyaya, A. (2013). Actin-mediated feedback loops in B-cell receptor signaling. Immunol. Rev. *256*, 177–189.

Spillane, K.M., and Tolar, P. (2017). B cell antigen extraction is regulated by physical properties of antigen-presenting cells. J. Cell Biol. *216*, 217–230.

Straight, A.F., Cheung, A., Limouze, J., Chen, I., Westwood, N.J., Sellers, J.R., and Mitchison, T.J. (2003). Dissecting temporal and spatial control of cytokinesis with a myosin II Inhibitor. Science *299*, 1743–1747.

Suzuki, K., Grigorova, I., Phan, T.G., Kelly, L.M., and Cyster, J.G. (2009). Visualizing B cell capture of cognate antigen from follicular dendritic cells. J. Exp. Med. *206*, 1485–1493.

Tedder, T.F., Inaoki, M., and Sato, S. (1997). The CD19-CD21 complex regulates signal transduction thresholds governing humoral immunity and autoimmunity. Immunity *6*, 107–118.

Vascotto, F., Lankar, D., Faure-André, G., Vargas, P., Diaz, J., Le Roux, D., Yuseff, M.I., Sibarita, J.B., Boes, M., Raposo, G., et al. (2007). The actin-based motor protein myosin II regulates MHC class II trafficking and BCR-driven antigen presentation. J. Cell Biol. *176*, 1007–1019.

Yau, I.W., Cato, M.H., Jellusova, J., Hurtado de Mendoza, T., Brink, R., and Rickert, R.C. (2013). Censoring of self-reactive B cells by follicular dendritic cell-displayed self-antigen. J. Immunol. *191*, 1082–1090.

Cell Reports, Volume *23*

Supplemental Information

Myosin IIa Promotes Antibody Responses by Regulating B Cell Activation, Acquisition of Antigen, and Proliferation

Robbert Hoogeboom, Elizabeth M. Natkanski, Carla R. Nowosad, Dessislava Malinova, Rajesh P. Menon, Antonio Casal, and Pavel Tolar

Supplemental Figures

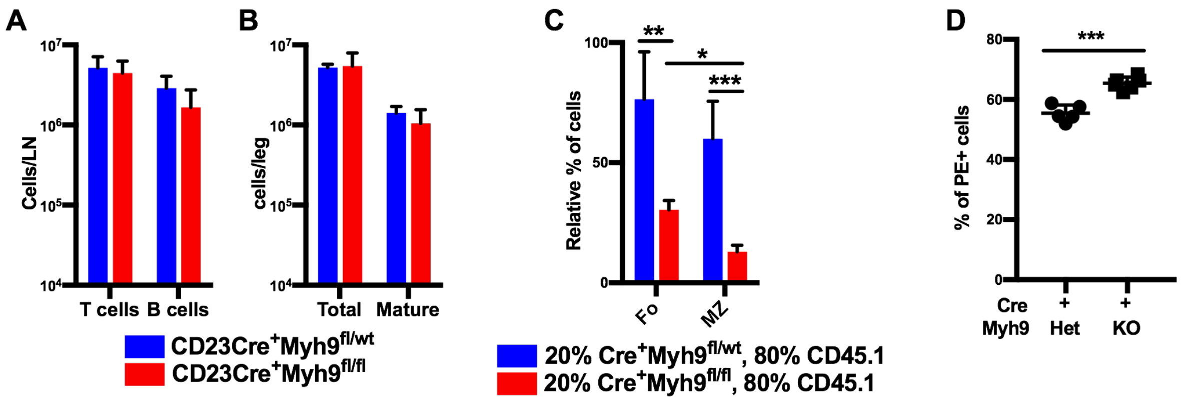

Figure S1. B cell numbers in secondary lymphoid organs of CD23Cre^{+}Myh9$^{fl/fl}$ mice, related to Figure 1.
(A) Quantification of lymph node (LN) T and B cells in CD23Cre^{+}Myh9$^{wt/fl}$ (n=6) and CD23Cre^{+}Myh9$^{fl/fl}$ (n=4) mice.
(B) Quantification of total and mature B cells in the bone marrow (BM) of CD23Cre^{+}Myh9$^{wt/fl}$ (n=6) and CD23Cre^{+}Myh9$^{fl/fl}$ (n=4) mice.
(C) Percentage of CD23Cre^{+}Myh9$^{wt/fl}$ and CD23Cre^{+}Myh9$^{fl/fl}$ Follicular (Fo) and Marginal Zone (MZ) B cells in competitive BM chimeras reconstituted in Rag1-KO mice (n=5 mice). Frequency normalized to percentage of CD23Cre^{+}Myh9$^{wt/fl}$ and CD23Cre^{+}Myh9$^{fl/fl}$-derived T cells.
(D) Percentage of labelled MZ B cells in tamoxifen treated R26ERT2Cre^{+}Myh9$^{wt/fl}$ and R26ERT2Cre^{+}Myh9$^{fl/fl}$ BM chimeras 5 min after injection with anti-CD19-PE antibody.
Mean ± SD. * $P<0.05$, ** $P<0.01$, *** $P<0.001$ (unpaired t-test)

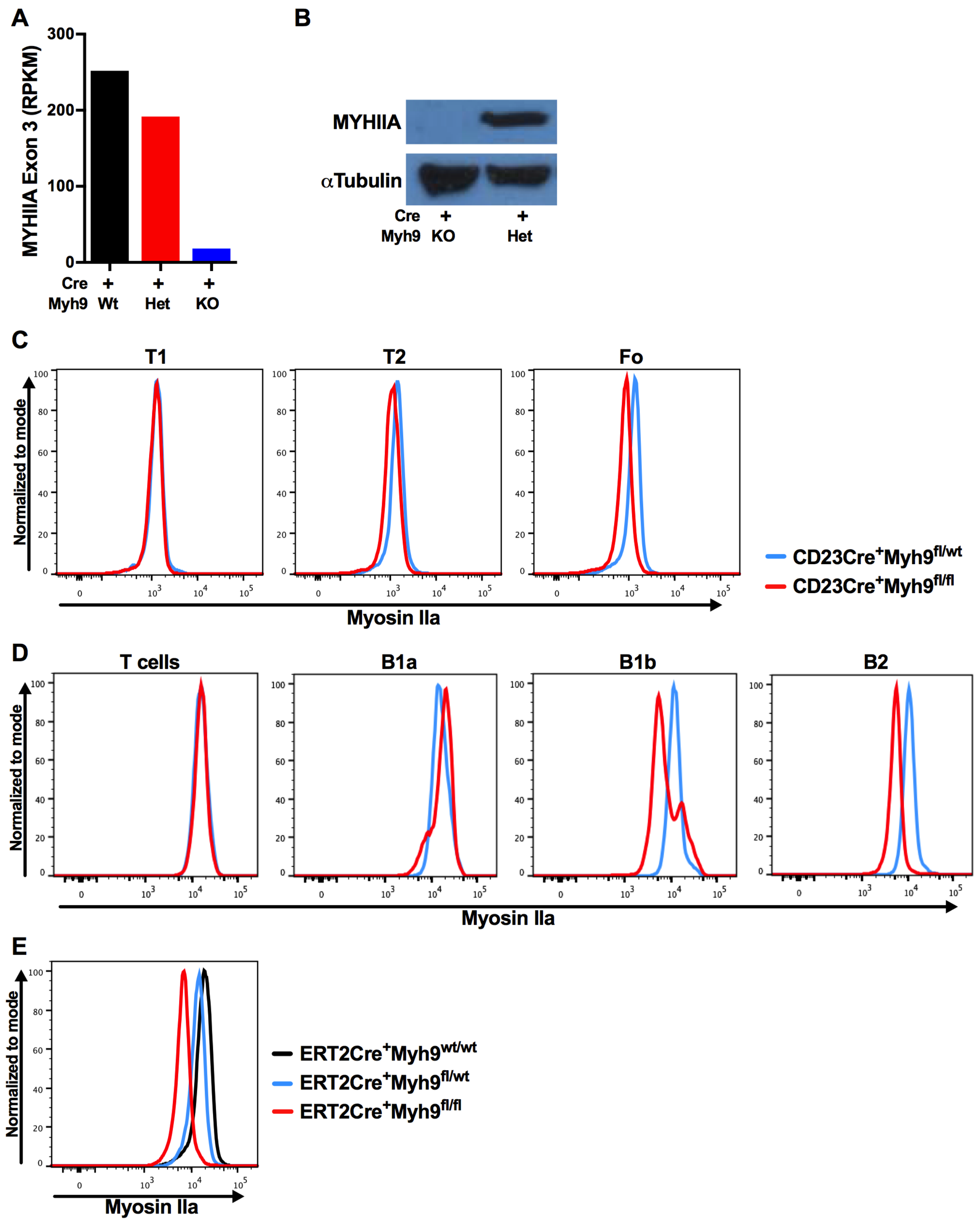

Figure S2. Myosin deletion in B cells of Mb1Cre^{+}Myh9$^{fl/fl}$, CD23Cre^{+}Myh9$^{fl/fl}$ and tamoxifen-treated R26ERT2Cre^{+}Myh9$^{fl/fl}$ mice, related to Figure 1.

(A) Myosin IIa mRNA expression levels of FACS-sorted follicular B cells from CD23Cre^{+}Myh9$^{wt/wt}$, CD23Cre^{+}Myh9$^{wt/fl}$ and CD23Cre^{+}Myh9$^{fl/fl}$ mice (n=3 mice).

(B) Myosin IIa protein expression level of FACS-sorted follicular B cells from CD23Cre^{+}Myh9$^{wt/fl}$ and CD23Cre^{+}Myh9$^{fl/fl}$ mice.

(C) Flow cytometric analysis of myosin IIa protein expression of T1, T2 and follicular (Fo) B cells of CD23Cre^{+}Myh9$^{wt/fl}$ and CD23Cre^{+}Myh9$^{fl/fl}$ mice.

(D) Flow cytometric analysis of myosin IIa protein expression of peritoneal cavity T, B1a, B1b and B2 cells of CD23Cre^{+}Myh9$^{wt/fl}$ and CD23Cre^{+}Myh9$^{fl/fl}$ mice.

(E) Flow cytometric analysis of myosin IIa protein expression of follicular B cells of R26ERT2Cre^{+}Myh9$^{wt/wt}$, R26ERT2Cre^{+}Myh9$^{wt/fl}$ and R26ERT2Cre^{+}Myh9$^{fl/fl}$ mice 14 days after the start of tamoxifen administration.

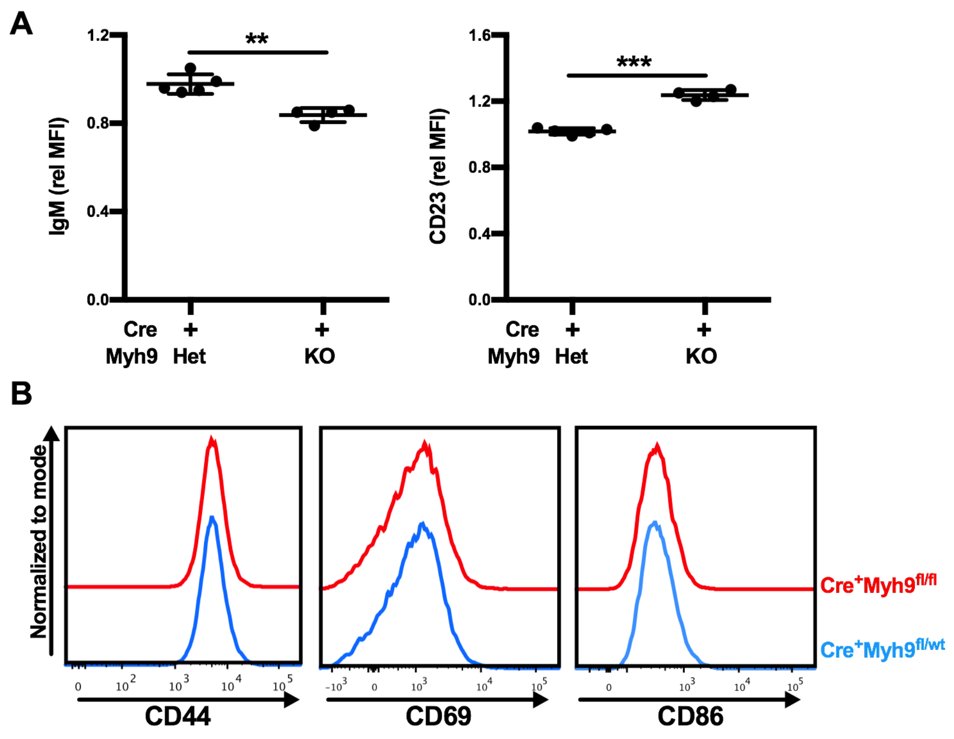

Figure S3. Follicular B cell surface marker expression of CD23Cre^{+}Myh9$^{fl/fl}$ mice, related to Figure 2.
(A) Surface expression of IgM and CD23 on follicular B cells from 20% CD23Cre^{+}Myh9$^{wt/fl}$, 80% CD45.1 and 20% CD23Cre^{+}Myh9$^{fl/fl}$, 80% CD45.1 competitive bone marrow chimeras. Normalized on expression levels of CD45.1-derived follicular B cells.
(B) Surface expression of CD44, CD69 and CD86 on follicular B cells of CD23Cre^{+}Myh9$^{wt/fl}$ and CD23Cre^{+}Myh9$^{fl/fl}$ mice.
Dots represent follicular cells from individual mice. Horizontal bars reflect mean ± SD. ** P<0.01, *** P<0.001 (Unpaired t-test).

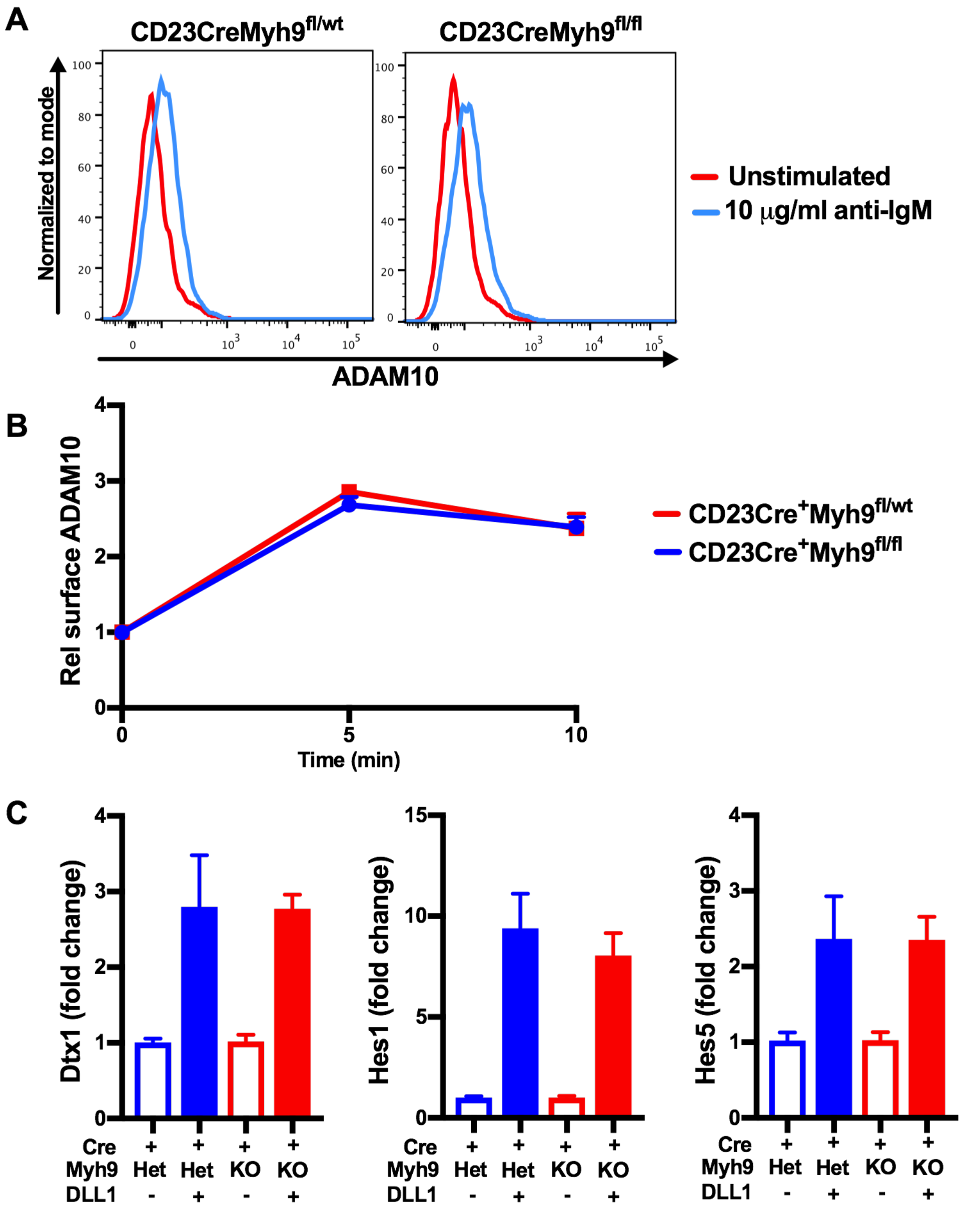

Figure S4. ADAM10 surface translocation and Notch2 signaling in CD23Cre^{+}Myh9$^{fl/fl}$ mice, related to Figure 2.

(A) Flow cytometric analysis of surface ADAM10 expression by B cells of CD23Cre^{+}Myh9$^{wt/fl}$ and CD23Cre^{+}Myh9$^{fl/fl}$ mice stimulated with soluble anti-IgM for 5 minutes.

(B) Quantification of surface ADAM10 expression by B cells of CD23Cre^{+}Myh9$^{wt/fl}$ and CD23Cre^{+}Myh9$^{fl/fl}$ mice stimulated with soluble anti-IgM.

(C) Gene expression level of Notch2 target genes *Dtx1*, *Hes1* and *Hes5* by B cells of CD23Cre^{+}Myh9$^{wt/fl}$ and CD23Cre^{+}Myh9$^{fl/fl}$ mice after 21h co-culture with OP9 that express or do not express Delta-like ligand1 (DLL1). Data normalized on expression levels on OP9 cells not expressing DLL1.

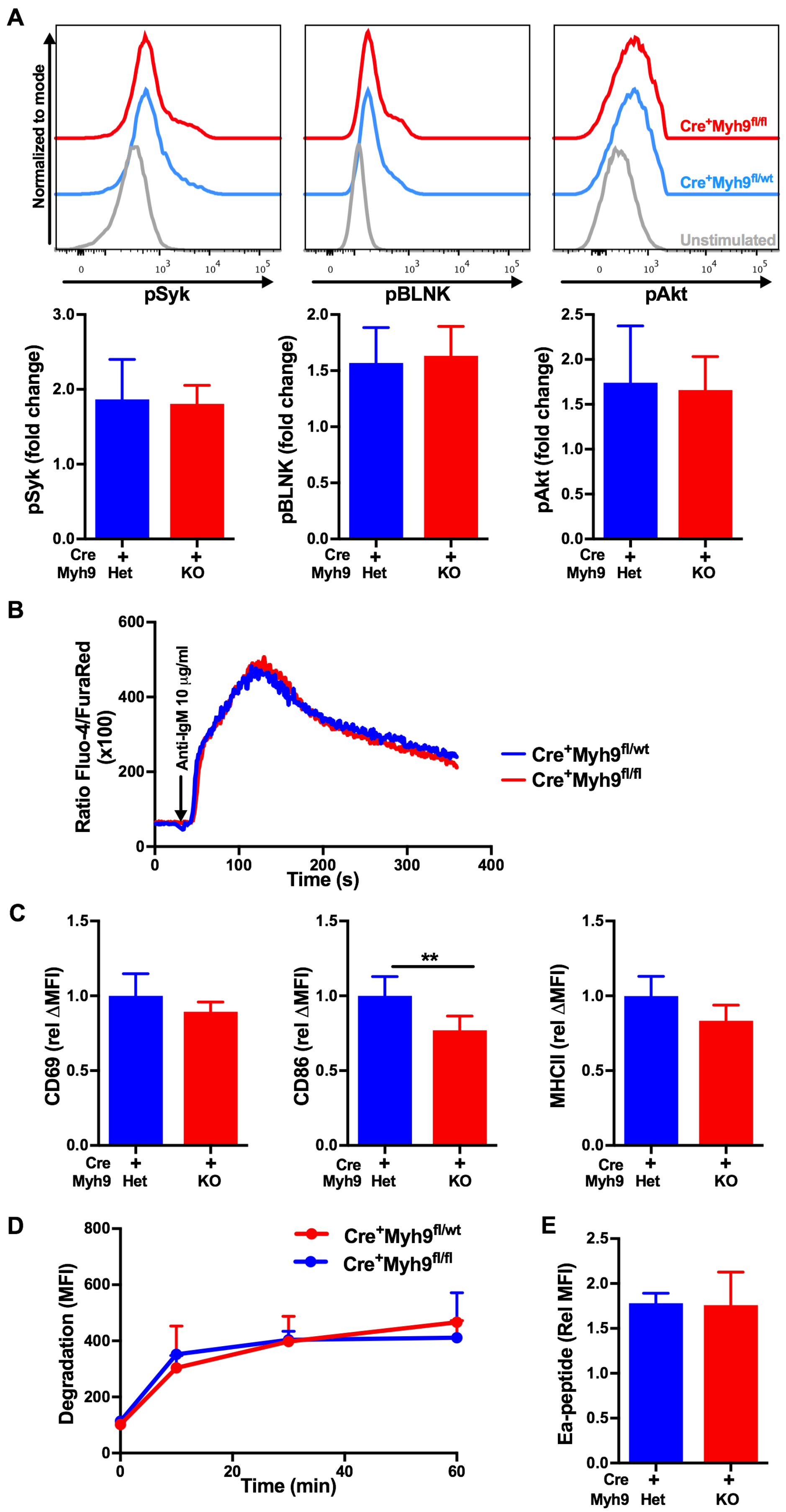
A
Normalized to mode
pSyk
pBLNK
pAkt
Cre+Myh9fl/fl
Cre+Myh9fl/wt
Unstimulated
pSyk (fold change)
pBLNK (fold change)
pAkt (fold change)
Cre + +
Myh9 Het KO
B
Ratio Fluo-4/FuraRed (x100)
Anti-IgM 10 μg/ml
Time (s)
Cre+Myh9fl/wt
Cre+Myh9fl/fl
C
CD69 (rel ΔMFI)
CD86 (rel ΔMFI)
MHCII (rel ΔMFI)
**
D
Degradation (MFI)
Time (min)
Cre+Myh9fl/wt
Cre+Myh9fl/fl
E
Ea-peptide (Rel MFI)

Figure S5. Normal levels of BCR signaling and antigen internalization of myosin IIa-deficient B cells in response to soluble antigen, related to Figure 3.

(A) Phosphorylation of Syk, BLNK and Akt in B cells of $CD23Cre^{+}Myh9^{wt/fl}$ and $CD23Cre^{+}Myh9^{fl/fl}$ mice stimulated with soluble anti-IgM for 5 (Syk, BLNK) or 15 min (Akt) and quantification (n=5 mice). Phosphorylation levels normalized on unstimulated cells.

(B) Intracellular Ca^{2+} fluxes of B cells of $CD23Cre^{+}Myh9^{wt/fl}$ and $CD23Cre^{+}Myh9^{fl/fl}$ mice after the addition of soluble anti-IgM (arrow).

(C) Upregulation of CD69, CD86 and MHCII in B cells of $CD23Cre^{+}Myh9^{wt/fl}$ and $CD23Cre^{+}Myh9^{fl/fl}$ mice after stimulation with soluble anti-IgM (n=5 mice). Data normalized on follicular B cells of $Cre^{+}Myh9^{wt/fl}$ littermates.

(D) Degradation of a DNA sensor attached to soluble anti-Igκ by B cells from $CD23Cre^{+}Myh9^{wt/fl}$ and $CD23Cre^{+}Myh9^{fl/fl}$ mice (representative for 2 experiments).

(E) Antigen presentation of $CD23Cre^{+}Myh9^{wt/fl}$ and $CD23Cre^{+}Myh9^{fl/fl}$ B cells as measured by MHCII-bound Ea peptide 5 hours after incubation with Ea peptide and anti-Igκ loaded microbeads (representative for 2 experiments). Data normalized on cells incubated with anti-Igκ loaded microbeads without Ea peptide.

Mean ± SD. ** $P<0.01$ (unpaired t-test).

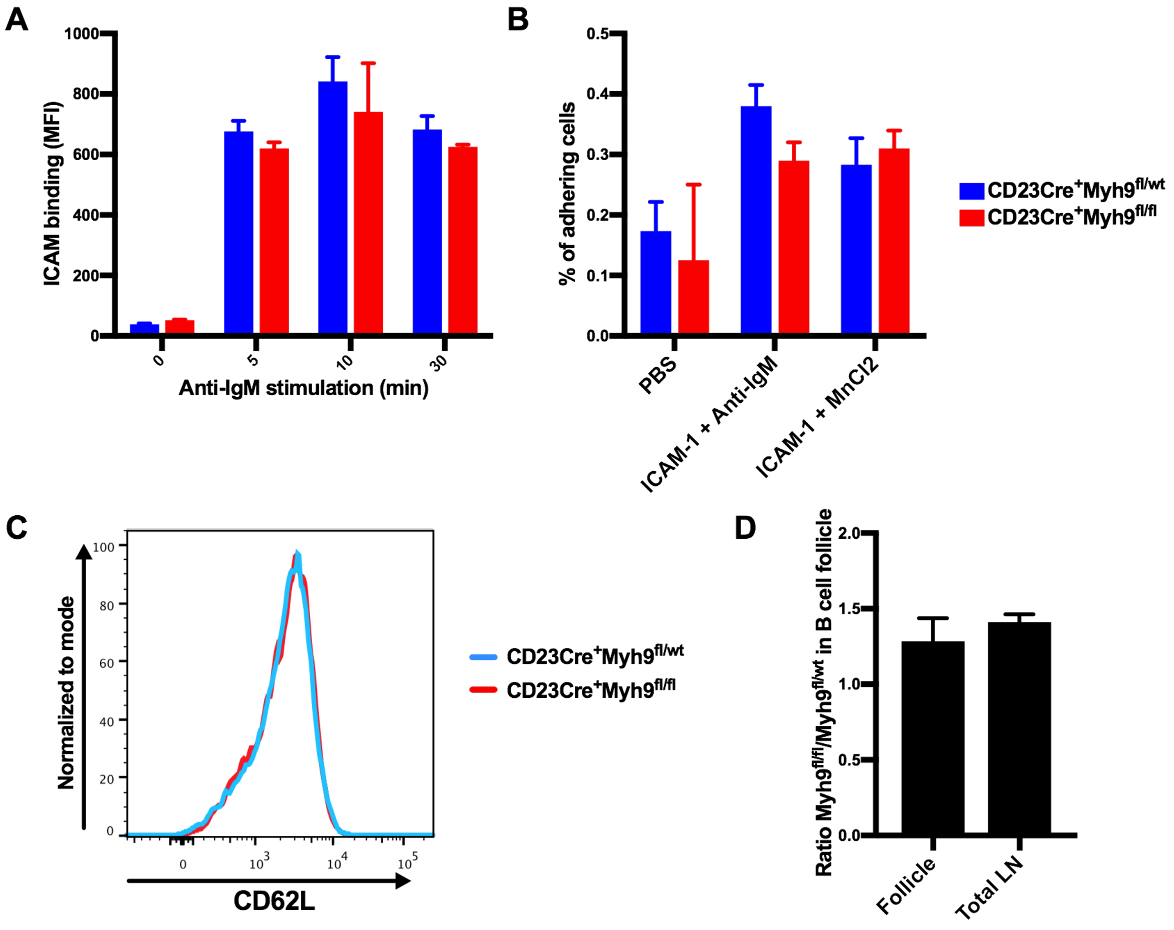

Figure S6: Initial adhesion to ICAM-1 is normal in myosin IIa-deficient B cells, related to Figure 4.

(A) Binding of soluble ICAM-1 to anti-IgM-stimulated B cells from CD23Cre^{+}Myh9$^{wt/fl}$ and CD23Cre^{+}Myh9$^{fl/fl}$ mice. Mean ± SD.

(B) Adhesion of anti-IgM and $MnCL_2$-stimulated B cells from CD23Cre^{+}Myh9$^{wt/fl}$ and CD23Cre^{+}Myh9$^{fl/fl}$ mice to immobilized ICAM-1. Mean ± SD.

(C) Surface expression of CD62L in CD23Cre^{+}Myh9$^{wt/fl}$ and CD23Cre^{+}Myh9$^{fl/fl}$ B cells.

(D) Ratio of B cells from CD23Cre^{+}Myh9$^{fl/fl}$ and CD23Cre^{+}Myh9$^{wt/fl}$ in B cell follicles 16h after i.v. injection in to C57BL/6J mice determined by immunofluorescence staining of LN cryosections and in the entire LN as assessed by flow cytometry. Ratios normalized to input ratio.

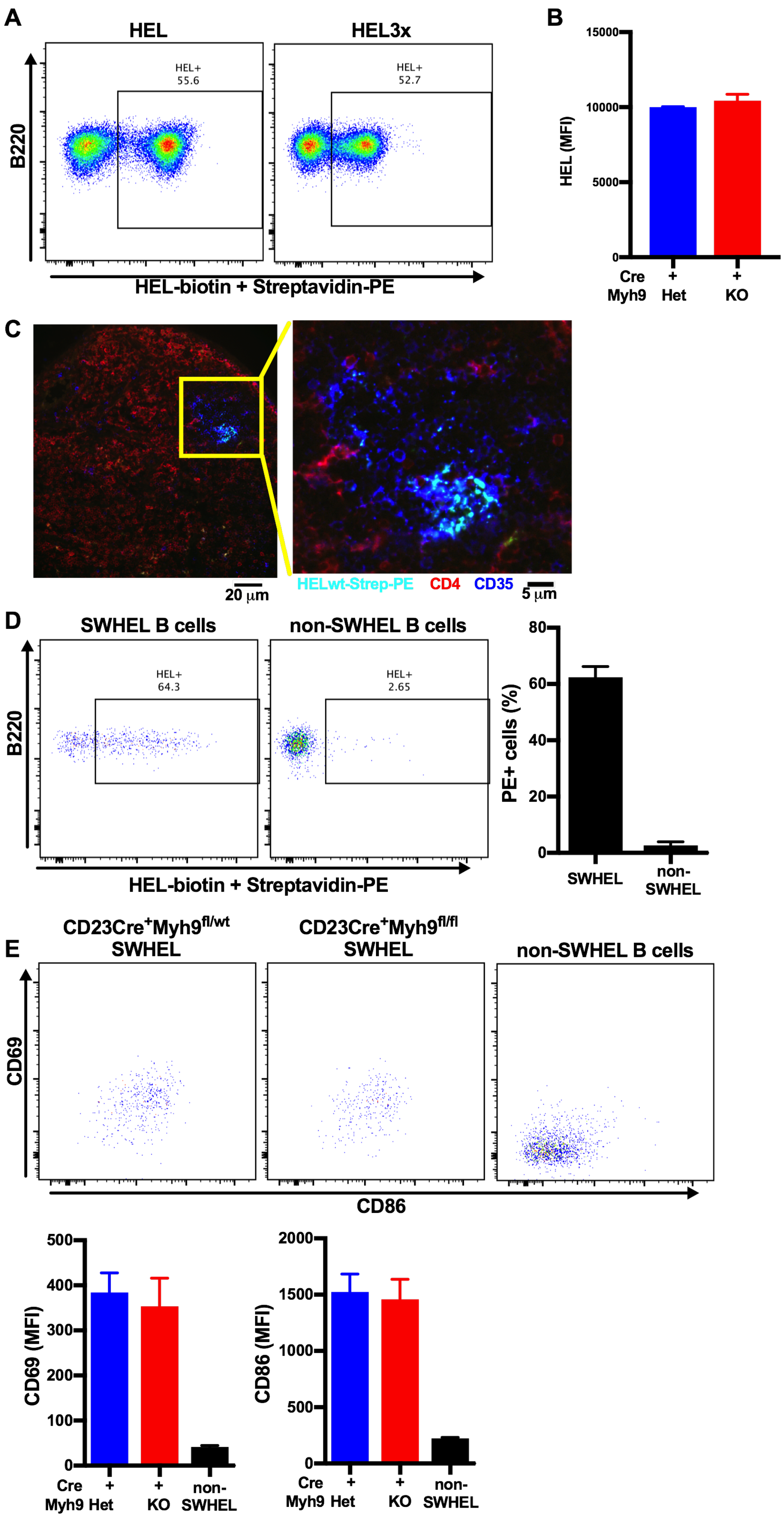

A
HEL
HEL3x
HEL+
55.6
HEL+
52.7
B220
HEL-biotin + Streptavidin-PE
B
15000
10000
5000
0
HEL (MFI)
Cre + +
Myh9 Het KO
C
20 µm
HELwt-Strep-PE CD4 CD35
5 µm
D
SWHEL B cells
non-SWHEL B cells
HEL+
64.3
HEL+
2.65
B220
HEL-biotin + Streptavidin-PE
80
60
40
20
0
PE+ cells (%)
SWHEL
non-SWHEL
E
CD23Cre+Myh9fl/wt SWHEL
CD23Cre+Myh9fl/fl SWHEL
non-SWHEL B cells
CD69
CD86
500
400
300
200
100
0
CD69 (MFI)
Cre + +
Myh9 Het KO non-SWHEL
2000
1500
1000
500
0
CD86 (MFI)
Cre + +
Myh9 Het KO non-SWHEL

Figure S7. *In vivo* antigen acquisition, related to Figure 5.
(A) Binding of HEL and HEL3x by $CD23Cre^{+}Myh9^{fl/wt}SW_{HEL}$ and $CD23Cre^{+}Myh9^{fl/fl}SW_{HEL}$ B cells *in vitro*.
(B) Quantification of HEL binding to $CD23Cre^{+}Myh9^{fl/wt}SW_{HEL}$ and $CD23Cre^{+}Myh9^{fl/fl}SW_{HEL}$ follicular B cells in vitro.
(C) HEL-Streptavidin-PE complexes in sections of lymph nodes of immunized mice.
(D) Flow cytometric analysis and quantification of HEL-Streptavidin-PE complex capture by B cells that express the SW_{HEL} BCR and non-SW_{HEL} expressing B cells.
(E) Flow cytometric analysis and quantification of CD69 and CD86 expression by PE-positive $CD23Cre^{+}Myh9^{fl/wt}SW_{HEL}$ and $CD23Cre^{+}Myh9^{fl/fl}SW_{HEL}$ or non-SW_{HEL} expressing B cells 14 hours after transfer into HEL-Streptavidin-PE immunized CD45.1 mice.
Mean ± SD.

Table S1. Gene expression analysis of FACS-sorted follicular B cells from CD23Cre^{+}Myh9$^{wt/fl}$ and CD23Cre^{+}Myh9$^{fl/fl}$ mice (n=3), related to Figure 2

Significant: $p<0.05$, $q<0.05$.

**Downregulated**				
**Gene name**	**CD23Cre^{+}Myh9$^{wt/fl}$ (RPKM)**	**CD23Cre^{+}Myh9$^{fl/fl}$ (RPKM)**	**log2(fold_change)**	**significant**
Lcn2	12.5893	1.20524	-3.38481	yes
Ngp	13.4001	1.29461	-3.37165	yes
Igj	5.39508	0.617053	-3.12818	yes
Beta-s	51.9653	7.08096	-2.87553	yes
Hbb-b1, Hbb-b2	19.2241	3.22646	-2.57489	yes
S100a8	145.112	25.7663	-2.49361	yes
S100a9	174.91	34.5411	-2.34023	yes
Hsph1	128.76	40.3277	-1.67484	yes
Cxcr4	113.087	39.7103	-1.50985	yes
Spns2	12.775	4.99355	-1.35518	yes
Ddit4	22.5883	8.9473	-1.33605	yes
Hspa4l	13.2209	5.34704	-1.30601	yes
Myh9	50.6481	21.1252	-1.26154	yes
P4ha1	8.67193	3.66499	-1.24254	yes
Wfdc17	52.5784	22.504	-1.22429	yes
Pde3b	20.0566	9.11876	-1.13717	yes
Sik1	19.2062	8.89217	-1.11097	yes
Slc5a3	5.66703	2.7072	-1.06579	yes
Fcrl5	12.7514	6.16063	-1.0495	yes
Chordc1	81.7667	40.4015	-1.01711	yes
Zfp36l2	172.465	86.872	-0.989344	yes
Gpr171	163.275	84.7899	-0.945336	yes
Hepacam2	9.81395	5.09649	-0.945329	yes
A530032D15Rik	27.1144	14.1881	-0.934384	yes
Dusp1	15.2936	8.1218	-0.913059	yes
Stip1	54.3244	29.034	-0.903856	yes
Apoe	81.8546	44.7237	-0.872025	yes
Zfp53	12.4342	7.01072	-0.826674	yes
Mavs	25.3436	14.3302	-0.822559	yes
Ets2	16.6178	9.42273	-0.818515	yes
Plac8	137.563	82.0615	-0.745315	yes
Ahsa1	95.2149	57.763	-0.721043	yes
**Upregulated**				
**Gene name**	**CD23Cre^{+}Myh9$^{wt/fl}$ (RPKM)**	**CD23Cre^{+}Myh9$^{fl/fl}$ (RPKM)**	**log2(fold_change)**	**significant**
C2cd5	3.88898	12.1419	1.64253	yes
Crisp3	6.58979	14.0753	1.09486	yes
Cirbp	30.4702	60.7195	0.99476	yes
Il12a	18.6664	36.8476	0.98113	yes
Hrsp12	16.7785	32.1925	0.940112	yes
Slamf1	23.8185	40.2892	0.758312	yes
Slc14a1	17.166	28.51	0.731918	yes
Tnf	12.3634	22.3179	0.852123	yes

Supplemental experimental procedures

Antibodies and flow cytometry

For flow cytometric analysis, erythrocyte-lysed single cell suspensions were stained with appropriate antibodies for 20 min on ice, followed by fixation with 2% paraformaldehyde (PFA). Antibodies against the following proteins were used to identify mouse B cell subsets and stain for activation markers: B220 (RA3-6B2), CD2 (RM2-5), CD69 (H1.3F2), CD86 (GL1) and CD95/Fas (Jo2) from BD Biosciences; CD5 (53-7.3), CD19 (1D3), CD23 (B3B4), CD38 (90), CD93 (AA4.1), IgM (II/41) and MHCII (M5/114.15.2) from eBioscience; CD21 (7E9), CD45.1 (A20) and CD45.2 (104) from BioLegend; ADAM10 (139712) from Novus Biologicals. Live/Dead fixable near-IR Dead Cell stain (ThermoFisher) was used to exclude dead cells. Cells were analyzed on BD FACSCanto II or Fortessa analyzers and sorted on Beckman Coulter MoFlo XDP cell sorters. Data was analyzed using FlowJo 10.0.7 (TreeStar).

Signaling, activation and proliferation

For signaling, activation, imaging and cell culture experiments, cell suspensions were enriched for B cells by negative selection using anti-CD43 beads (Miltenyi) on an autoMACS cell separator (Miltenyi).

To analyze phosphorylation of BCR signaling pathway components, B cells were stimulated with 10 µg/ml anti-IgM (Jackson ImmunoResearch), fixed with 2% PFA, permeabilized with the Foxp3 fixation/permeabilization kit (eBioscience) and stained with phosphorylation-specific antibodies for Syk (C87C1), Erk (D13.14.4E), Akt (193H12) and BLNK (J117-1278), all from BD Biosciences. To investigate intracellular Ca^{2+} fluxes, B cells were labeled with 1 µM Fluo-4 and 1 µM FuraRed (both from ThermoFisher), B220 antibody (RA3-6B2) and live/dead fixable near-IR Dead Cell stain (ThermoFisher). Baseline fluorescence of the dyes was analyzed for 25 seconds by flow cytometry. Then, 10 µg/ml of anti-IgM was added (Jackson ImmunoResearch) and fluorescence of the dyes was acquired for a further 300 seconds. To analyze activation, B cells were stimulated overnight with 10 µg/ml anti-IgM, fixed in 2% PFA and stained for surface activation markers.

To study proliferation, B cells were labeled with 1 µM 5-chloromethylfluorescein diacetate (CMFDA, ThermoFisher) and cultured for 48 hours in DMEM supplemented with 10% fetal calf serum (BioSera), 100 µM non-essential amino acids, 20 mM HEPES, 2 mM glutamine, 50 µM beta-mercaptoethanol, 100 U/ml penicillin and 100 µg/ml streptomycin. When indicated cells were stimulated with 10 µg/ml anti-IgM (Jackson ImmunoResearch), 10 µg/ml LPS (Sigma), 100 µM CpG (Sigma), 1 µg/ml CD40L (R&D Systems) or 100 ng/ml IL-4 (Peprotech). Dilution of CMFDA was analyzed by flow cytometry. To analyze cell cycle progression, cells were stimulated with 100 µM CpG for 48 hours, fixed in PFA, stained for surface markers and permeabilized with the FoxP3 permeabilization kit. Then, DNA was stained with propidium iodide (Sigma) and analyzed by flow cytometry.

***In vitro* antigen internalization and presentation**

Internalization of soluble antigen was analyzed using DNA-based degradation sensors as described (Nowosad et al., 2016). In short, 100 nM of complementary DNA oligomers, one strain labeled with Atto647N and one with quencher Iowa Black RQ, were annealed and attached to anti-Igκ (Southern Biotech) and incubated with purified B cells on ice. After washing, cells were incubated at 37ºC and unquenching of Atto647N fluorescence was analyzed by flow cytometry.

To detect antigen presentation, B cells were incubated with Ea-peptide (Biotin-GSGFAKFASFEAQGALANIAVDKA-COOH) and anti-Igκ-loaded microbeads (Bangs laboratories) for 5 hours at 37ºC. Cells were fixed with 2% PFA and stained with anti-MHCII-Ea antibody (M5/114.15.2, eBioscience) followed by a fluorescently labeled anti-rat IgG2b antibody (ThermoFisher).

Large scale imaging

PMS were prepared by shearing off HEK293 cells seeded in poly-L-lysine coated Lab-Tek imaging chambers (ThermoFisher) by sonication, as described (Natkanski et al., 2013). Subsequently, remaining membrane patches were blocked with 1% bovine serum albumin (BSA) and decorated with an in-house biotinylated and Cy3-labeled goat anti-mouse Igκ $F(ab')_2$ antibody using Annexin V-biotin (BioVision) and streptavidin (Sigma). B cells were labeled with either Cell Trace Violet (CTV) or Cell Trace Far Red (CTFR) (both from ThermoFisher) and mixed with unlabeled control cells or vice versa. Cells were resuspended in warm 0.1% BSA in Hanks-buffered salt solution (HBSS) and incubated in pre-warmed imaging chambers for indicated times. Incubation was stopped by adding PFA. After blocking with normal mouse serum (Jackson ImmunoResearch) cells were stained for B220, followed by permeabilization with the Foxp3 fixation/permeabilization kit and staining for phosphorylated BCR signaling pathway components.

Automated image acquisition was performed by a motorized IX81 microscope with a 100x objective (Olympus) and motorized stage with integrated piezo Z-drive, both controlled by Metamorph software (Molecular Devices). Illumination was supplied by 405 nm (Changchun New Industries), 488 nm, 514 nm (both from LS300, Dynamic

Lasers) and 640 nm lasers (Blue Sky Research). Up to 900 fields of view were acquired per imaging chamber. Images were processed and analyzed using Matlab (Mathworks)-based algorithms developed as described (Nowosad et al., 2016). In short, interactive modules were programmed for (1) determination and correction of background, flatfield and spectral bleedtrough; (2) processing and analysis of three-dimensional multichannel images and identification of cells; (3) display and gating of data. Algorithms are available on request.

Migration and adhesion

To analyze binding of soluble ICAM-1, B cells were stimulated with 10 µg/ml anti-IgM or 10 mM $MnCl_2$ in the presence of soluble ICAM-1-Fc chimera protein (R&D systems) complexed to fluorescently-labeled anti-IgG. Cells were fixed with PFA after 5, 10 and 30 minutes and binding of ICAM-1 was analyzed by flow cytometry. To determine adhesion to immobilized ICAM-1, 96-well plates were coated with 5 µg/ml ICAM-1-Fc and blocked with 2% BSA in PBS. Purified B cells were labeled with CTV, resuspended in 0.1% BSA in HBSS and allowed to adhere in the presence of 10 µg/ml anti-IgM or 0.5 mM $MnCl_2$ for 30 minutes at 37ºC. Adherence of cells was analyzed by measuring CTV fluorescence.

To study migration by timelapse imaging, Lab-Tek imaging chambers (ThermoFisher) were coated with ICAM-1-Fc chimera protein and blocked with 2% BSA in PBS. B cells were activated with 1 µg/ml CXCL13 (R&D systems) for 15 minutes and added to warmed ICAM-1-coated chambers containing 1 µg/ml CXCL13 in 0.1% BSA in HBSS. Cells were allowed to adhere for 10 minutes and then brightfield images were acquired every 5 seconds for 40 minutes. TrackMate was used to identify and track cells (Tinevez et al., 2017).

Transwell migration assays were performed using 96 well Transwell inserts with 5 µm polycarbonate membranes (Sigma-Aldrich). Top compartments were coated with 0.5 µg/ml ICAM-1 or 1 µg/ml anti-Igκ $F(ab')_2$ in PBS, followed by blocking with 2% BSA in PBS. 80.000 cells were added to top compartments and allowed to migrate towards 1 µg/ml CXCL13, 0.5 µg/ml CXCL12 or 1 µg/ml CCL21 (all from R&D systems) in 0.1% FBS in RPMI in the bottom compartment for 3 hours. Migrated B cells were quantified using flow cytometry.

Gene expression analysis

RNA was isolated from sorted follicular B cells using TriZol (ThermoFisher) and further purified using a RNEasy mini kit (Qiagen). After RNA amplification (Ovation RNA-Seq system V2, NuGen) stranded polyA-enriched libraries were prepared using a TruSeq Nano DNA kit (Illumina), followed by sequencing on a HiSeq 2500 (Illumina). Reads were aligned to C57BL/6J mouse reference genome mm10, GRCm38 and quantified using Tophat and Cufflinks, as described elsewhere (Roberts et al., 2012).

To analyze expression of Notch target genes, RNA was extracted and reverse transcribed using the RNEasy mini and Quantitect Reverse Transcription kits, respectively. Gene expression was analyzed using Taqman technology, probes Mm00492297_m1 (*Deltex1*), Mm01342805_m1 (*Hes1*) and Mm00439311_g1 (*Hes5)* on a Quantstudio 3 Real-Time PCR machine (Applied Biosystems). Data was normalized on expression of *HPRT* (probe Mm00446968).

Immunizations and ELISA

Mice were immunized by i.p. injection of 50 µg NP-Ficoll or NP-CGG (BioSearch) suspended in alum (ThermoFisher). Serum blood samples were withdrawn before and 7 and 10 days after immunization. To detect NP-specific antibodies by ELISA, nunc polysorb plates were coated with 5 µg/ml NP_{13}-BSA (BioSearch), blocked with 3% BSA in PBS and incubated with serial dilutions of serum, followed by incubation with HRP-labeled anti-IgM, anti-IgG1 or anti-IgG3 antibodies (Southern Biotech). Bound HRP was analyzed using 3,3',5,5'-tetra-methylbenzidine (TMB). Steady-state serum immunoglobulin levels were determined using the SBA Clonotyping System-C57BL/6-HRP kit (Southern Biotech).

References

Natkanski, E., Lee, W.-Y., Mistry, B., Casal, A., Molloy, J.E., Tolar, P., 2013. B Cells Use Mechanical Energy to Discriminate Antigen Affinities. Science 340, 1587–1590.

Nowosad, C.R., Spillane, K.M., Tolar, P., 2016. Germinal center B cells recognize antigen through a specialized immune synapse architecture. Nat Immunol 17, 870–877.

Roberts, A., Goff, L., Pertea, G., Kim, D., Kelley, D.R., Pimentel, H., Salzberg, S.L., Rinn, J.L., Pachter, L., Trapnell, C., 2012. Differential gene and transcript expression analysis of RNA-seq experiments with TopHat and Cufflinks. Nature Protocols 7, 562–578.

Tinevez, J.-Y., Perry, N., Schindelin, J., Hoopes, G.M., Reynolds, G.D., Laplantine, E., Bednarek, S.Y., Shorte, S.L., Eliceiri, K.W., 2017. TrackMate: An open and extensible platform for single-particle tracking. Methods 115, 80–90.
